# Photodegradable Antimicrobial Agents: Synthesis and Mechanism of Degradation

**DOI:** 10.1021/acs.joc.2c00681

**Published:** 2022-06-02

**Authors:** Vebjørn Eikemo, Bjarte Holmelid, Leiv K. Sydnes, Magne O. Sydnes

**Affiliations:** † Department of Chemistry, Bioscience and Environmental Engineering, Faculty of Science and Technology, University of Stavanger, Stavanger NO-4036, Norway; ‡ Department of Chemistry, University of Bergen, Bergen NO-5007, Norway

## Abstract

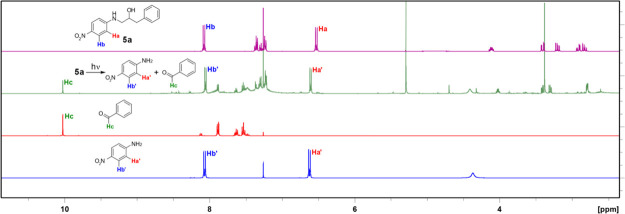

As a strategy to inactivate antimicrobial agents after use, we designed a range of ethanolamine derivatives where four of them possessed interesting activity. The ethanolamine moiety facilitates photodecomposition, which in a potential drug will take place after use. Herein, the synthetic preparation of these compounds and the mechanism of photoinactivation are described.

## Introduction

Since Alexander Fleming discovered penicillin in 1928,^1^ the application of this antibiotic has saved millions of lives,^2,3^ but excessive use of this and other similar drugs in agriculture and human medicine has resulted in the development of antibiotic-resistant bacteria strains.^4^ As a result, many infections that used to be easy to cure, such as pneumonia and postoperative infections, have gradually become a threat.^5,6^ Multidrug resistant (MDR) bacteria are already a global problem, and several health organizations describe the situation as critical,^2,7,8^ whereas Mah calls antimicrobial resistance (AMR) a silent pandemic in a recent commentary.^9^ Data from 2019 show that more than 1.2 million people died as a direct result of infections with MDR bacteria,^10^ and the future situation looks a lot worse with resistance levels projected to rise.^11−13^ Despite this prediction, pharmaceutical companies seem uninterested in developing new antibiotics because these drugs are not regarded as profitable because they will probably not be used unless existing treatments fail totally.^14^

**Figure 1:**
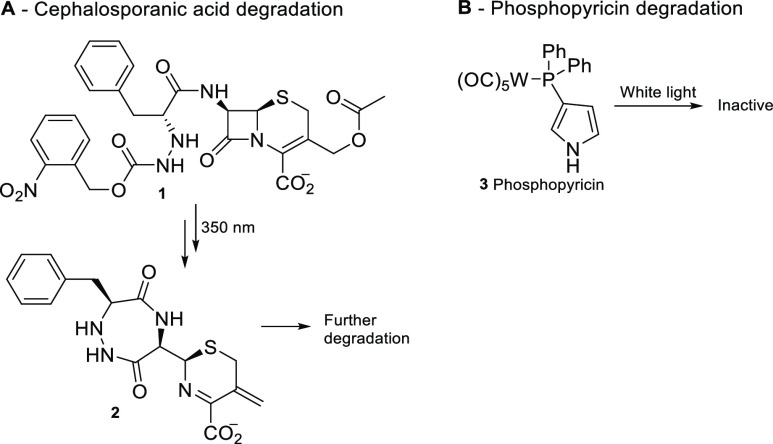
Photodegradable antibiotics: (A) Cephalosporanic acid; (B) phosphopyricin.

A consequence of the enormous consumption of antibiotics is high levels of pharmaceuticals and their metabolites in drinking water, waste water, ground water, and coastal waters as well as marine organisms.^15−19^ This has led to environmental exposure to antibacterial agents such as ciprofloxacin, sulfamethoxazole, chloramphenicol, and amoxicillin,^20−26^ which contribute to the development of AMR. In recent years, technologies have been developed to address this issue. A promising tool is photopharmacology, which applies light to activate and deactivate drugs.^27−29^ Another approach is light-induced degradation of drugs of which the photodecomposition of a cephalosporanic acid (**1**) (Figure 1A) is an example;^30^ excitation of the nitrobenzyl carbamate moiety leads to ring opening of the β-lactam and formation of hydrazide **2**. Another example is the phosphine-tungsten complex phosphopyricin (**3**), which is degraded and deactivated when exposed to white light (Figure 1B).^31^

We recently communicated the synthesis and biological evaluation of four antimicrobial agents based on a new scaffold, which facilitated light-induced fragmentation leading to the formation of inactive and nontoxic compounds.^32^ This chemistry opens up the possibility to prepare new antimicrobial agents that decompose in the environment after release. Here, a full account of the synthetic work and studies of the photodecomposition mechanisms is presented.

## Results and Discussion

### Synthesis of Model Compounds

**Scheme 1:**
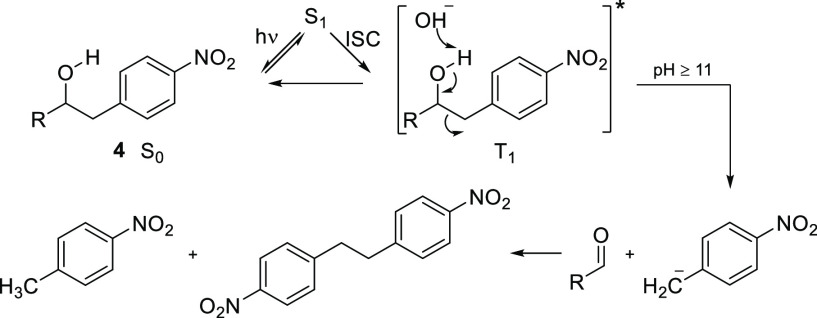
Photo-Retro-Aldol-Type Reaction of Nitrophenethyl Alcohols R = Ph, CH_3_. Irradiation with a 254, 300, or 350 nm lamp yields the first excited singlet state (S_1_) of the molecules, which undergoes intersystem crossing to the reactive triplet state (T_1_).

Our investigation was inspired by the work of Wan and Muralidharan who studied the so-called photo-retro-aldol reaction, summarized in Scheme 1.^33,34^ The idea was to incorporate similar benzylic-alcohol motifs into biologically active molecules that would undergo the same photofragmentation and furnish biologically inactive fragments after use.

**Scheme 2:**
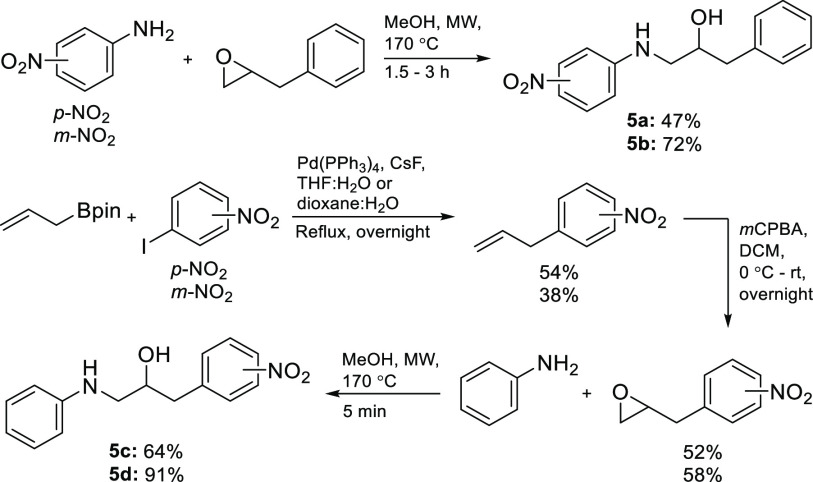
Preparation of Model Compounds **5a**–**5d**

As a starting point, we decided to study model compounds closely related to those investigated by Wan and Muralidharan. Candidates were arrived at by attaching an *N*-arylaminomethyl group to C1 in ethanol derivative **4** (Scheme 2), which corresponds to the incorporation of an ethanolamine scaffold. Four such compounds were prepared by treating benzyloxiranes with three anilines under microwave irradiation. The aryl groups and the anilines contained either a phenyl or a nitrophenyl moiety, which were those utilized by Wan and Muralidharan.^33^ When benzyloxirane was reacted with *p*- and *m*-nitroaniline, the corresponding aminols, 1-(*p*-nitrophenylamino)-3-phenylpropan-2-ol (**5a**) and 1-(*m*-nitrophenylamino)-3-phenylpropan-2-ol (**5b**), were obtained in moderate yields (Scheme 2). The outcome was better when *p*- and *m*-nitrobenzylepoxide, prepared from allyl boronate by a Suzuki–Miyaura cross-coupling with nitroiodobenzenes according to Kotha and co-workers^35^ followed by treatment with *m*CPBA, were treated with aniline and gave 3-(*p*-nitrophenyl)-1-phenylaminopropan-2-ol (**5c**) and 3-(*m*-nitrophenyl)-1-phenylaminopropan-2-ol (**5d**) in moderate overall yield (Scheme 2). The reason for this is probably the better nucleophilicity of aniline compared to the nitroanilines. Attempts were made to improve the yield of the Suzuki–Miyaura allylation by running the reaction under anhydrous conditions, but they all failed.

**Figure 2:**
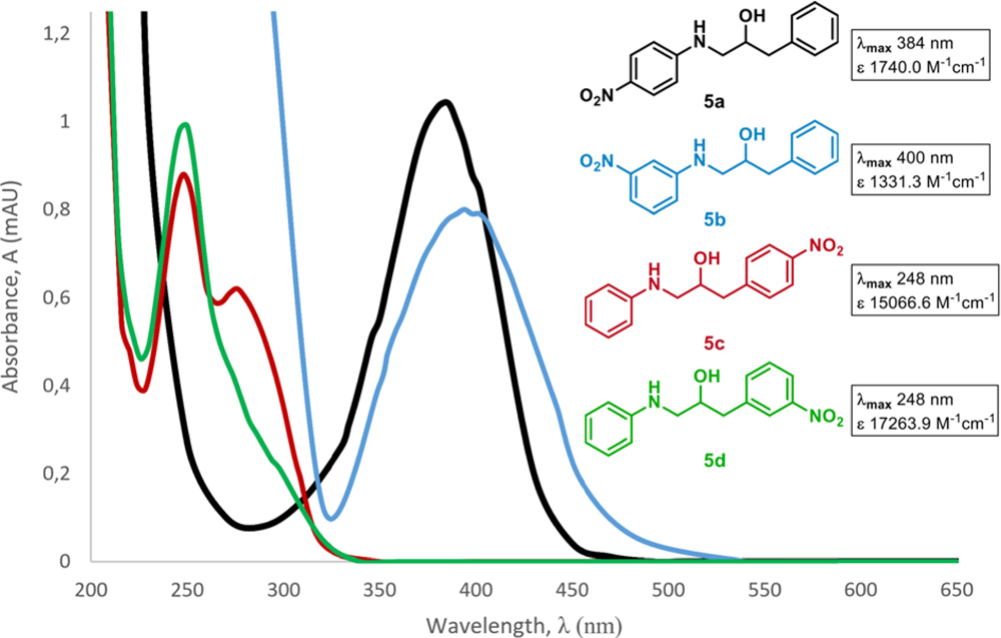
UV/vis spectroscopic data for compounds **5a**–**5d**.

A requirement for photodecomposition of the aminols in the environment is absorption of light with wavelengths above approximately 300 nm. The UV spectra of **5a**–**5d** were therefore recorded, and this revealed that whereas **5a** and **5b**, both with a nitrophenylamino moiety attached to C1, had a strong absorption with λ_max_ around 400 nm, **5c** and **5d** barely absorbed light above 330 nm (Figure 2). Under natural conditions, therefore, aminols **5a** and **5b** should absorb light the best and be more prone than **5c** and **5d** to suffer light-induced decomposition.

#### Photodegradation Studies of **5a**–**5d**

Following the procedure by Wan and Muralidharan,^33^ aminoalcohols **5a**–**5d** in a mixture of acetonitrile (ACN) and water (7/3, v/v) were irradiated for 2 h through a Pyrex filter over a wide pH range. Following liquid–liquid extraction, all reaction mixtures were analyzed by ^1^H NMR spectroscopy, and this revealed dramatic differences in decomposition for the different compounds (Table 1). When the nitro group is *para* to the amino moiety (**5a**), a 100% conversion is achieved at pH ≥ 11, whereas the *meta* analogue (**5b**) undergoes only 11% degradation at pH 11 and 17% at pH 13. At pH lower than 7, only trace amounts of decomposition products were detected by ^1^H-NMR analysis for compound **5b**. For compounds **5c** and **5d**, a pH ≥ 11 is required for degradation to occur.

**Table 1:** Percent (%) Conversion of Compounds **5a**–**5d** at Various pH Values as Determined by ^1^H-NMR Analysis^a^^,^^b^

compound	**5a** (%)	**5b** (%)	**5c** (%)	**5d** (%)
pH 1	9	trace	ND	ND
pH 3	9	trace	ND	ND
pH 5	9	trace	ND	ND
pH 7	32	4	ND	ND
pH 9	75	5	ND	ND
pH 11	100	11	38	20
pH 13	100	17	40	56

a Conversions are obtained from normalized integral values for the *o*-proton on the aniline.

b ND = no consumption detected.

**Figure 3:**
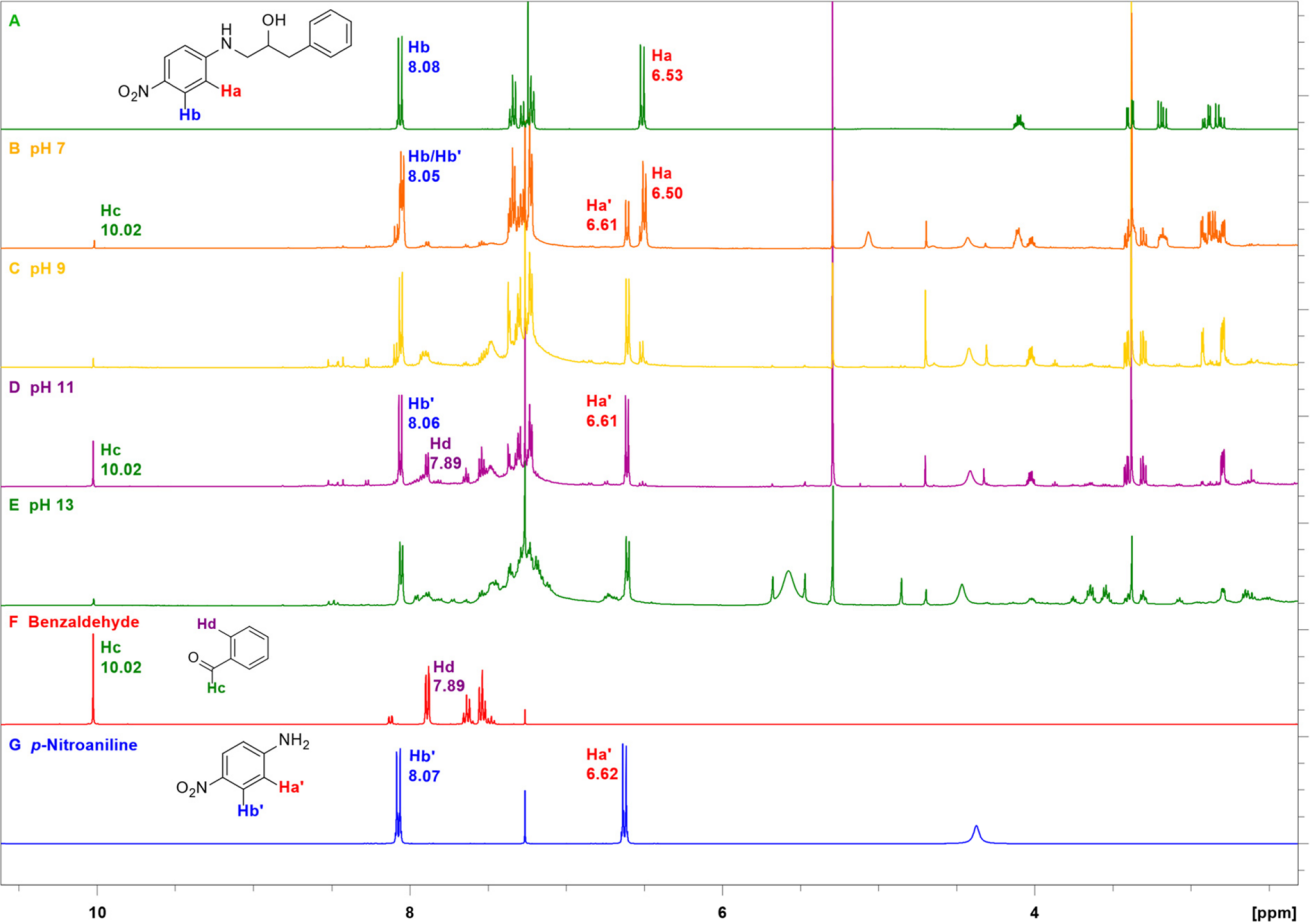
^1^H-NMR spectra of the photolysate from compound **5a** irradiated at pH from 7 to 13 for 2 h and reference spectra of benzaldehyde and *p*-nitroaniline.

Qualitative photodecomposition studies were then performed with ethanolamine **5a** at pH 7 and above using ^1^H NMR to monitor the reaction (Figure 3A–E). The development of an aldehyde singlet at δ 10.02 ppm and a doublet at 7.89 ppm (Figure 3D) is consistent with benzaldehyde formation (Figure 3F), and doublets at δ 6.61 and 8.06 ppm (Figure 3D) are in accordance with formation of *p*-nitroaniline (Figure 3G). At pH 13 (Figure 3E), the ratio between benzaldehyde and *p*-nitroaniline is much lower than that at pH 11 (Figure 3D), suggesting that further degradation took place. This is substantiated by a number of additional signals in the 5.8–5.5, 5.0–3.5, and 3.7–3.4 ppm regions. A conceivable product from benzaldehyde is benzoic acid, the formation of which is supported by a signal at 171.1 ppm in the ^13^C-NMR spectrum due to a carboxylate group. The rate of decomposition is clearly pH-dependent; it is peaking around pH 11 and gradually decreasing as the pH drops.

**Figure 4:**
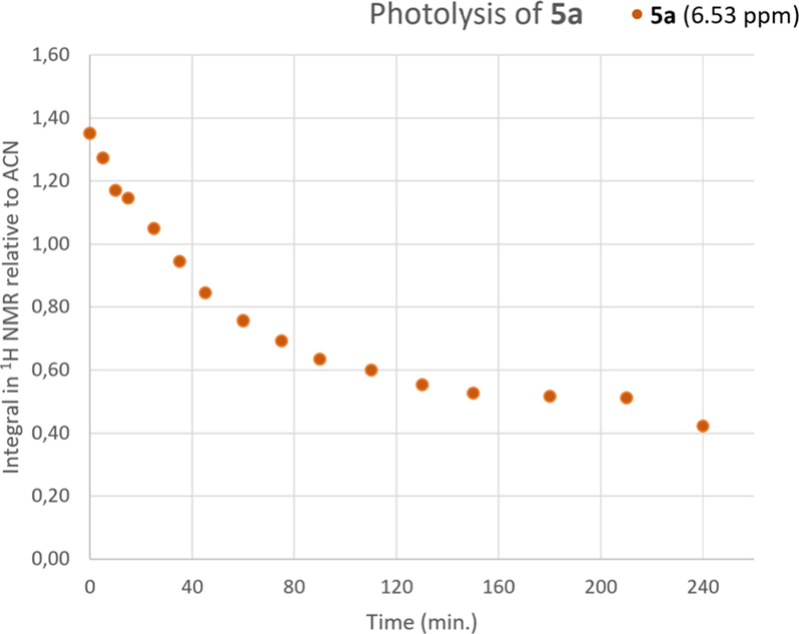
Rate of decomposition for compound **5a** upon irradiation in an NMR tube. The ^1^H-NMR signals due to residual proton(s) in the solvent (acetonitrile) were used as an internal standard.

To obtain quantitative data for the decomposition, compound **5a** dissolved in ACN-*d*_3_/water-*d*_2_ (7/3 v/v) was subjected to photolysis at pH 11 in a nuclear magnetic resonance (NMR) tube. ^1^H NMR spectra were recorded regularly and the residual proton signals from ACN were used as an internal standard. The integral of these signals was assumed to be constant throughout the experiment, and on this basis the smooth curve in Figure 4 was obtained for the decomposition of **5a** (supporting data can be found in Table S1). The curve clearly shows that the rate of decomposition is much slower in the NMR tube, and the substrate was not fully consumed even after 24 h of irradiation due to the formation of colored products that acted as a filter for the light. Nevertheless, after ca. 80 min half of the starting material was consumed. At 75 min, the ratio between the integral for the two *ortho* protons in the aniline moiety of **5a** and the integral for the corresponding protons in *p*-nitroaniline was ca. 7:4, which indicates that *p*-nitroaniline was the major product formed in addition to other compounds as can be seen in Figure 3D,E. The structures of these compounds are not known.

**Scheme 3:**
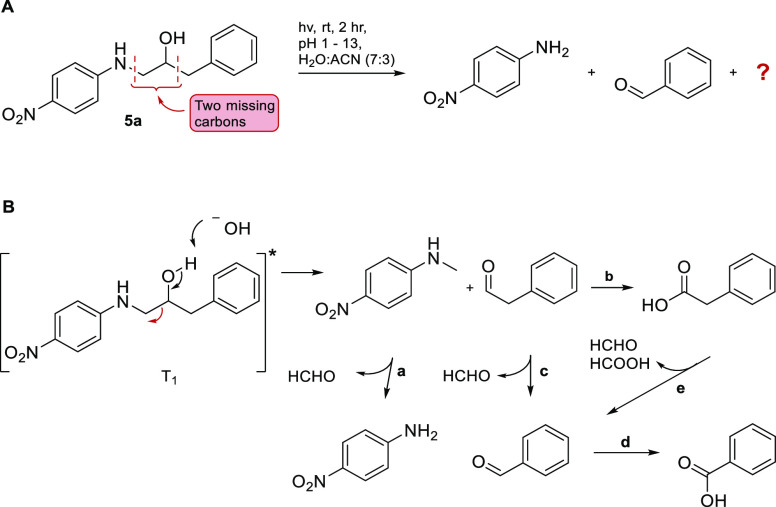
Suggested Reaction Pathways for Photodegradation of Compound **5a**

As two of the products are undoubtedly *p*-nitroaniline and benzaldehyde, two carbons are unaccounted for in the analysis of the reaction mixture (Scheme 3A). A reasonable assumption is that the two missing carbons form products that either diffuse into the aqueous phase or disappear as volatiles during workup. Conceivably, the suggested products originate from a photo-retro-aldol-type reaction, initially forming *N*-methyl-4-nitroaniline and phenylacetaldehyde (Scheme 3B). The former product, known to undergo photochemical *N*-demethylation,^36,37^ subsequently reacts and furnishes formaldehyde and *p*-nitroaniline (Scheme 3B, reaction a). Phenylacetaldehyde then undergoes oxidation to phenylacetic acid (Scheme 3B, reaction b) (which can suffer cleavage and form benzaldehyde and formaldehyde/formic acid (Scheme 3B, reaction e)^38^) or reacts in a Norrish type 1 process and forms formaldehyde and benzaldehyde (Scheme 3B, reaction c). Indeed, phenylacetaldehyde was converted to benzaldehyde when irradiated under the same conditions as those for compounds **5a**–**5d**.

**Scheme 4:**
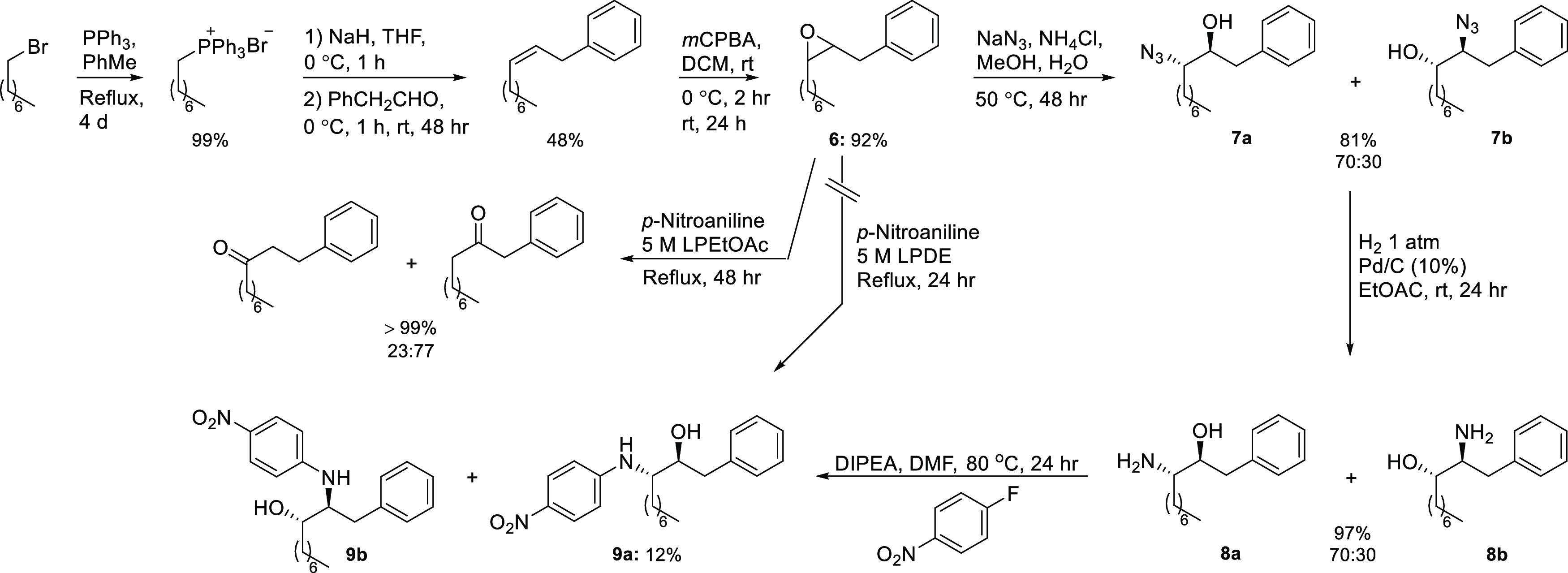
Synthesis of 3-Aminodecan-2-ol Derivative **9a** The relative stereochemistry for compounds **7**–**9** is indicated.

To shed more light on the decomposition process, we designed an analogue to **5a** with a long alkyl chain at C1 to prevent that one of the two carbon atoms not accounted for in the ethanolamine scaffold does not disappear during workup of the photolysate. The synthesis (Scheme 4) commenced with a Wittig reaction involving octyltriphenylphosphonium bromide and phenylacetaldehyde resulting in the formation of the desired compound as a 94:6 mixture of the *Z*/*E* isomers as evident from the integration of the doublets at 3.41 and 3.34 ppm in the ^1^H NMR spectra, respectively, a method used by Krasovskaya et al. to establish the *Z*/*E* ratio for similar products.^39^ When the isomeric mixture of the decene was subjected to *m*CPBA, the corresponding oxiranes (**6**) (assumed to be formed in a 94:6 *cis*/*trans* ratio) were obtained in 92% yield. Attempts were made to react **6** with *p*-nitroaniline, but due to the poor nucleophilicity of the latter combined with steric hindrance no reaction occurred in a 5 M solution of lithium perchlorate in diethyl ether (LPDE) at 40 °C. When compound **6** was treated with LPEtOAc and the temperature increased to 80 °C, a reaction took place, but not the right one; instead, a lithium-promoted epoxide-carbonyl rearrangement occurred, giving two decanones as the only products. However, treatment of **6** with sodium azide gave an inseparable mixture of the two azidodecanols **7a** and **7b** in a 7:3 ratio as evident from ^1^H NMR analysis, which upon hydrogenation and subsequent nucleophilic aromatic substitution with 1-fluoro-4-nitrobenzene afforded a mixture of the target compound 3-(4-nitrophenyl)amino-1-phenyldecan-2-ol (**9a**) and its regioisomer **9b**. The low isolated yield of the desired product, just 12%, was due to overlapping fractions.

If aminoalcohol **9a** undergoes degradation just like **5a** when irradiated, octanal should be present in the reaction mixture as should *p*-nitroaniline and benzaldehyde. This was indeed the case (see Figure S1 in the Supporting Information); in addition to the signals from the aromatic compounds, the ^1^H-NMR spectrum of the hydrolysate showed an aldehyde triplet at 9.76 ppm with a coupling constant of 1.9 Hz and COSY correlations to the alkyl chain. After 3 days at room temperature, the aldehyde was completely converted to the corresponding carboxylic acid, as evident from a HMBC correlation from the alpha methylene protons at 2.32 ppm to a carbonyl signal at 173.7 ppm and further confirmed to be octanoic acid by low-resolution mass spectrometry (LRMS).

### Synthesis of Additional Ethanolamine Derivatives

**Scheme 5:**
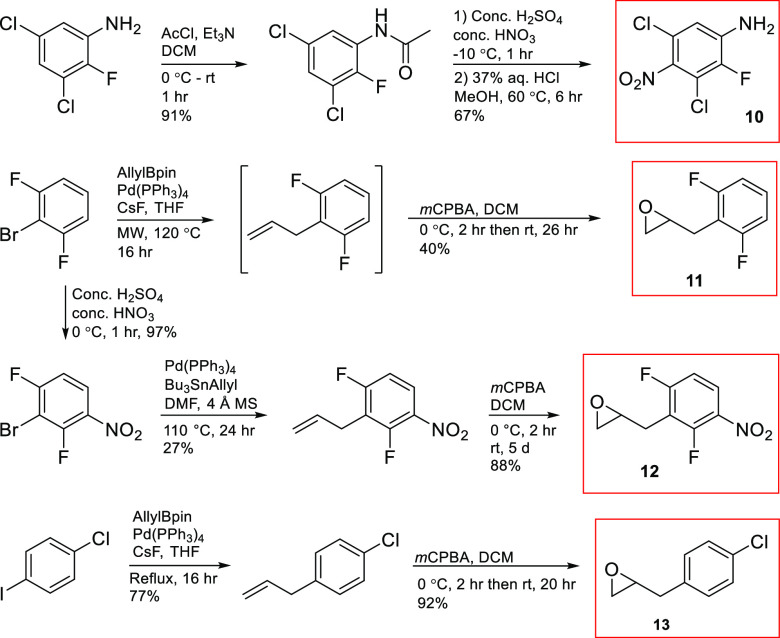
Synthesis of Some Key Building Blocks for the Preparation of Additional Ethanolamine Derivatives

With the photodecomposition process essentially proved, we started the search for biologically active analogues to **5a** by reacting benzyloxirane derivatives with anilines containing aryl groups that have been found in other molecules showing biological activity. To make a range of compounds, some key building blocks, one aniline and three epoxides, were prepared as summarized in Scheme 5.^32^ Aniline **10** was synthesized from the corresponding trihaloaniline by performing acetylation followed by nitration and acidic hydrolysis, which furnished **10** in 61% overall yield. Two of the epoxides were prepared from 1-bromo-2,6-difluorobenzene. A standard Suzuki–Miyaura cross-coupling with allylboronic acid pinacol ester furnished 2-allyl-1,3-difluorobenzene, which was treated with *m*CPBA to afford 2,6-difluorobenzyloxirane (**11**) in 40% yield over two steps. The other was 2,6-difluoro-3-nitrobenzyloxirane (**12**), which was obtained in 23% overall yield by nitration followed by a Stille cross-coupling with allyltributylstannane and then epoxidation with *m*CPBA. Finally, the conversion of *p*-chloroiodobenzene to epoxide 4-chlorobenzyloxirane (**13**) was achieved in 71% overall yield by the same synthetic route that was used to prepare **11**.

**Scheme 6:**
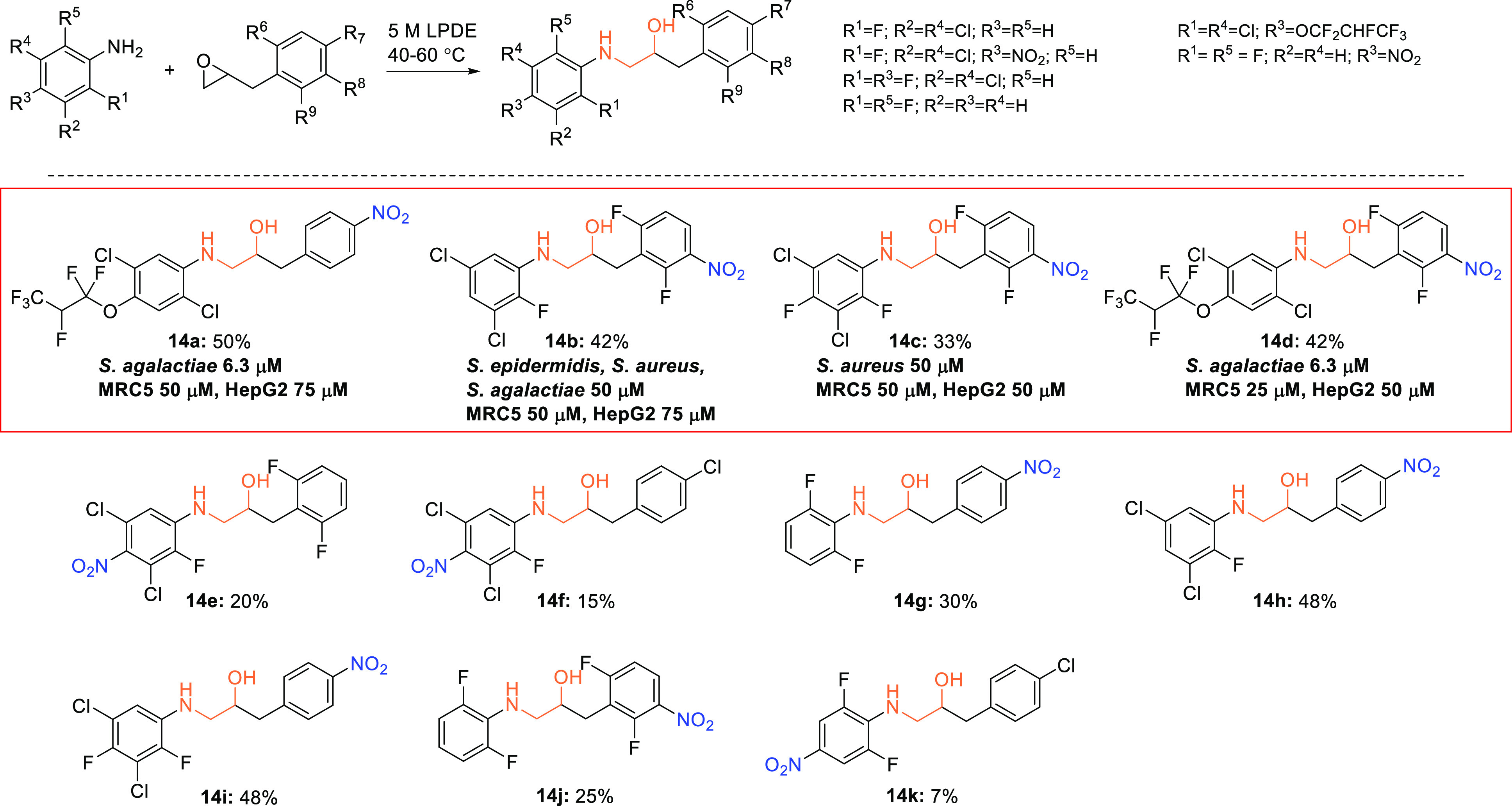
Preparation of Aminols with R^1^ = F, Cl; R^2^ = Cl, H; R^3^ = NO_2_, F, H, OCF_2_CHFCF_3_; R^4^ = Cl, H; R^5^ = F, H; R^6^ = F, H; R^7^ = NO_2_, Cl, H; R^8^ = NO_2_, H, and R^9^ = F, H

**Scheme 7:**
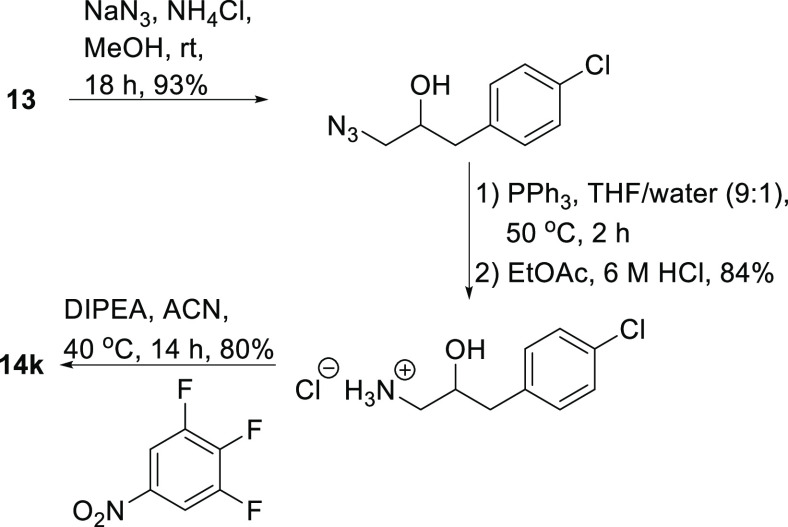
Formation of **14k** using Method B

With these building blocks at hand, a number of target molecules could be synthesized using a Lewis acid-promoted epoxide ring-opening reaction in a 5 M LPDE solution, to which the anilines and epoxides were added (Scheme 6). No product was obtained in better than 50% yield, conceivably because the anilines react sluggishly because they are rather poor nucleophiles due to the electron-withdrawing substituents. The worst case was observed when 2,6-difluoro-4-nitroaniline reacted with oxirane **13** and gave the expected ethanolamine, 3-(4-chlorophenyl)-1-(2,6-difluoro-4-nitrophenyl)aminopropan-2-ol (**14k**) in 7% yield only. Another synthesis of **14k** was therefore attempted, based on a reversal of the roles of the nucleophile and electrophile (Scheme 7). Thus, ring-opening of epoxide **13** with sodium azide followed by a Staudinger reduction gave the desired 1,2-amino alcohol, which reacted readily with 1,2,3-trifluoro-5-nitrobenzene to yield the target compound in 80% yield.

#### Screening of Biological Activity

As already reported, ethanolamine derivatives **14a**–**14d** have been screened against selected Gram-positive and Gram-negative bacteria, including *Escherichia faecalis*, *E. coli*, *Pseudomonas aeruginosa*, *Staphylococcus aureus*, *S. agalactiae*, and *S. epidermidis*, and showed promising antibacterial activity with a minimum inhibitory concentration (MIC) as low as 6.3 μM in the best cases (**14a** and **14d**) (Scheme 6).^32^ It was therefore very disappointing when all of the remaining seven analogues were totally inactive (MIC > 100 μM). More compounds have to be prepared and tested before the reason for this difference can be elucidated, but four noteworthy trends have been observed. First, all the active compounds have a nitro group attached to the aryl group closer to the hydroxyl group. Then, none of the compounds with a nitroaryl group attached to the amino substituent (**14e**, **14f**, and **14k**) exhibit any activity. Furthermore, only the most active compounds have an alkoxyaryl group attached to the amino substituent. Finally, the biological activity (and lack of activity) is the result of impact from both aryl moieties because even though **14g**–**14j** have a nitroaryl motif closer to the OH group, they are totally inactive even at a 100 μM level. On the basis of these observations, additional compounds will be synthesized and tested.

#### Photochemical Decomposition of Aminols **14a**–**14d**

The four active compounds, viz. aminols **14a**-**14d**, were subjected to photodecomposition at pH 8 and 13. All four compounds degraded completely at pH 13, with the exception of compound **14d**, which displayed 56% conversion. Amino alcohol **14a** decomposed following the same reaction pathway as compound **5a**, yielding 2,5-dichloro-4-(1,1,2,3,3,3-hexafluoropropoxy)aniline and 4-nitrobenzoic acid (Figure S2 in the Supporting Information). Stability studies performed in the dark for compounds **14a**–**14d** at pH 13 revealed that **14b**, **14c**, and **14d** are somewhat unstable in the basic environment. An S_N_Ar reaction with hydroxide occurs, substituting one of the fluorine atoms, which was confirmed by LRMS, ^1^H NMR, and ^19^F NMR. At pH 13, around half of the starting material underwent this unwanted reaction and the rest photodecomposed. However, at pH 8 this was not a problem because all compounds were stable for at least 24 h at room temperature. To our satisfaction, we found that compounds **14b** and **14d** were completely consumed in the photochemical reaction at pH 8 (Table 2), but they were not following the photo-retro-aldol pathway described for aminol **5a** and confirmed by compound **9a**.

**Table 2:** Photodecomposition Conversions (%) at pH 8 and 13 for Compounds **14a**–**14d**^a^

compound	photolysis^a^		stability^b^	
	pH 8 (%)	pH 13 (%)	pH 13	pH 8
14a	25	100	yes	yes
14b	100	100	no	yes
14c	19	100	no	yes
14d	100	56	no	yes

a Conversions were estimated by ^1^H NMR spectra of the crude reaction mixtures after aqueous workup.

b Performed for 24 h in the dark at rt.

**Figure 5:**
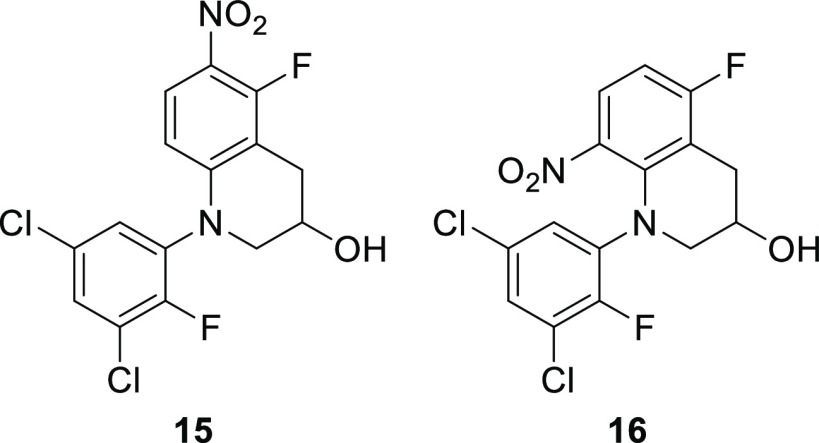
Structure of compounds **15** and **16**.

For aminol **14b**, an intramolecular S_N_Ar reaction occurs, yielding compound **15** (Figure 5), as evident from four sharp doublets of doublets appearing in the ^1^H NMR spectrum of the crude degradation mixture at 7.76, 7.58, 7.41, and 6.23 ppm (Figure S3 in the Supporting Information). There are only two fluorine atoms present according to ^19^F NMR (Figure S4B), and the ^3^*J*_HH_, ^4^*J*_HF_, and ^5^*J*_HF_ couplings of 9.3, 8.9, and 1.6 Hz, respectively, confirm this structure. The other possible isomer from an S_N_Ar reaction, compound **16**, would have displayed a larger ^3^*J*_HF_,^40^ but this is not observed (Figure S4B in the Supporting Information). A similar reaction occurred in the photodegradation of compound **14c**, as illustrated by the loss of one fluorine atom in the ^19^F NMR spectrum of the crude degradation mixture (Figure S4D in the Supporting Information), but the conversion was only 19% at pH 8. The same characteristic signals did not appear in the ^1^H NMR spectrum of the degradation of compound **14d**, suggesting that a different mechanism is involved in the degradation of **14d**. However, it was not possible to elucidate any degradation products due to a complicated NMR spectrum with many unresolved multiplets and broad signals indicating that the compound has fully decomposed. Research into this is currently ongoing.

The crude degradation mixtures derived from **14a**–**14d** displayed no activity against the same bacteria strains, as well as no toxicity against MRC5 and HepG2 cell lines.

#### Mass Spectrometric Analyses

The ease with which the compounds reported here could be studied by mass spectrometry was sensitive to the chemical structure. The key building blocks and intermediates were straightforward to analyze using classical electrospray ionization (ESI) methods, which delivered molecular cations or adducts (ESI positive mode) and deprotonated molecular anions (ESI negative mode) of high intensities when standard instrumental settings were applied. To compounds, viz. 1-phenyldec-2-ene, **11** and **12** deviated from this pattern and were ionized using classical electron ionization (EI) for detection and analysis of radical cations. However, based on experience from previous work, analysis of amino alcohols **5a**–**5d** and **14a**–**14k** was expected to be more challenging. Such nitro-substituted aromatics are prone to ionize both under positive and negative ion formation, and the choice of instrumental parameters plays a greater role. Not surprisingly, well established electrospray and atmospheric pressure chemical ionization (APCI) methods failed and forced us to investigate alternative ionization conditions. Increasing the ionization potential of both spray methods was fruitless, and a delicate control of in-source parameters was essential to avoid collisional induced dissociations. Enhanced potential of the preanalyzer ion-guide extensively produced mass spectra occupied by severe dissociations. In particular, in the case of amino alcohols **14a**–**14k**, mass spectra are composed of parent product ions missing the nitro group or one or more of the halogens. In all spray-ionization experiments (both ESI and APCI), methanol or ACN was initially used as a spray reagent. However, the solvent in ESI/APCI may strongly influence the ionization efficiency; we chose to explore different solvents and solvent pH. When optimizing for signal intensities in mass chromatograms, a solvent mixture consisting of 50% MeCN containing 0.1% HCOOH and 50% H_2_O containing 0.1% HCOOH gave excellent results. Mild ionization in negative ESI mode under such spraying conditions exclusively produced the deprotonated molecular anion of amino alcohols **5a**–**5d** and **14a**–**14k**.

## Conclusions

The synthesis of 12 compounds possessing our aminol scaffold that facilitates photodecomposition or photoinitiated S_N_Ar is described. Four of the compounds possessed antimicrobial activity, and their corresponding decomposition products had no antimicrobial activity or toxicity. Through NMR analysis we have elucidated the products derived from the photochemical reactions enabling the establishment of mechanisms for the formation of the photochemical products. Compound **14d**, which was one of the two most active compounds prepared, decomposes totally in the course of 24 h at pH 8 making this a very interesting lead for further optimization of biological activity. Work toward this end is ongoing in these laboratories.

## Experimental Section

### General Information

All chemicals were obtained from Sigma-Aldrich/Merck or VWR and used as supplied. When specified, solvents were dried by storing over 4 Å molecular sieves. For petroleum ether (pet. ether), the 40–60 °C fraction was used. All reactions were carried out under a nitrogen or argon atmosphere, unless otherwise specified. Microwave-assisted experiments were performed in a CEM Focused Microwave Synthesis System, model type Discover, operating at 0–300 W and pressure range of 0–290 psi.

Thin-layer chromatography (TLC) was carried out with silica gel (60 F_254_) on aluminium sheets with solvent systems consisting of various mixtures of pet. ether, ethyl acetate, and dichloromethane (DCM). Visualization was achieved with either UV light (254 and/or 365 nm) or a potassium permanganate stain. Flash chromatography was performed with a hand pump and 230–400 mesh silica gel or an Interchim Puriflash 215 autoflash chromatography system with Biotage Snap Ultra HP-Sphere 25 μm silica-gel columns.

IR spectra were recorded on an Agilent Cary 630 FT-IR spectrophotometer equipped with an attenuated total reflectance attachment. Samples were analyzed neat on a ZnSe crystal, and the absorption frequencies are given in wave numbers (cm^–1^).

UV–vis spectra were obtained on an Agilent 8453 single-beam UV–vis spectrophotometer with a deuterium-discharge lamp for the UV range and a tungsten lamp for the visible wavelength range. Samples were analyzed in an Agilent open-top UV quartz cell (10 mm, 3.0 mL) with ethanol as the solvent. The wavelengths are reported in nm and molar attenuation coefficients in M^–1^ cm^–1^.

NMR spectra were recorded on a Bruker Ascend 400 spectrometer (400.13 MHz for ^1^H, 100.61 MHz for ^13^C, 376.46 MHz for ^19^F, and 161.98 MHz for ^31^P). Coupling constants (*J*) are given in Hz, and the multiplicity is reported as singlet (s), doublet (d), triplet (t), quartet (q), sextet (s), multiplet (m), and broad singlet (bs). Chemical shifts are reported in ppm in the order downfield to upfield, and calibration is performed using the residual solvent signals for chloroform-*d* (^1^H 7.26 ppm; ^13^C 77.16 ppm), ACN-*d*_3_ (^1^H 1.94 ppm; ^13^C 1.32 ppm), or water-*d*_2_ (^1^H 4.79 ppm).^41^ Calibration for ^19^F NMR is performed using α,α,α-trifluorotoluene as the internal standard in chloroform-*d* (−62.61 ppm) and ACN-*d*_3_ (−63.10 ppm).^42^

High-resolution mass spectra were obtained on a JEOL AccuTOF T100GC mass spectrometer. The instrument was operated with an orthogonal ESI source, an orthogonal accelerated time-of-flight (TOF) single-stage reflectron mass analyzer, and a dual microchannel plate. Mass calibration was performed using the internal standard method, and mass drift compensation was performed in each acquisition. Low-resolution mass spectra were recorded on an Advion expression^S^ compact mass spectrometer operated in ESI mode equipped with a Plate Express TLC plate reader for sample injection. A solution of ammonium acetate (5.0 mM) and formic acid (0.05%) in ACN and water (95:5) was used as the mobile phase for both positive and negative ESI modes.

#### Synthesis of 1-(4-Nitrophenylamino)-3-phenylpropan-2-ol (**5a**)

A sealed reactor tube was charged with 4-nitroaniline (0.28 g, 2.0 mmol), (2,3-epoxypropyl)benzene (0.29 mL, 296 mg, 2.2 mmol), and methanol (0.50 mL). The reaction mixture was irradiated in a microwave reactor at 160–170 °C for 3 h. The mixture was evaporated onto celite, and the crude product was isolated by silica-gel flash chromatography (DCM/MeOH 98:2). Concentration of the relevant fractions (*R*_f_ = 0.43 in DCM/MeOH 99:1) yielded **5a** as a yellow solid (0.26 g, 47%, mp. 111–112 °C). IR (neat): ν_max_ 3392, 3029, 2921, 1600, 1307 cm^–1^; UV–vis: λ_max_ (EtOH) 384 nm (ε 1740 cm^–1^ M^–1^); ^1^H NMR (400.13 MHz, CDCl_3_): δ 8.08 (d, *J* = 9.2 Hz, 2H), 7.38–7.33 (m, 2H), 7.31–7.27 (m, 1H), 7.24–7.22 (m, 2H), 6.53 (d, *J* = 9.2 Hz, 2H), 4.15–4.09 (m, 1H), 3.41 (dd, *J* = 13.0 Hz, 3.4 Hz, 1H), 3.20 (dd, *J* = 13.0 Hz, 7.8 Hz, 1H), 2.92 (dd, *J* = 13.6 Hz, 5.1 Hz, 1H), 2.83 (dd, *J* = 13.6 Hz, 8.1 Hz, 1H); ^13^C{^1^H} NMR (100.61 MHz, CDCl_3_): δ 153.4, 138.3, 137.1, 129.4, 129.0, 127.2, 126.5, 111.5, 71.1, 48.4, 41.9; HRMS (ESI/TOF): calcd for C_15_H_15_N_2_O_3_^–^ [M – H]^−^ 271.10882, found 271.10866.

#### Synthesis of 1-(3-Nitrophenylamino)-3-phenylpropan-2-ol (**5b**)

A sealed tube was charged with 3-nitroaniline (0.28 g, 2.0 mmol), (2,3-epoxypropyl)benzene (0.27 mL, 275 mg, 2.0 mmol), and methanol (0.50 mL). The reaction mixture was irradiated in a microwave reactor at 170–180 °C for 90 min. The mixture was evaporated onto celite, and the crude product was isolated by flash column chromatography (DCM/pet. ether 7:3). Concentration of the relevant fractions (*R*_f_ = 0.45 in DCM/MeOH 99:1) yielded **5b** as a yellow solid (0.39 g, 72%, mp. 74–77 °C). IR (neat): ν_max_ 3545, 3302, 3081, 2915, 1618 cm^–1^; UV–vis: λ_max_ (EtOH) 400 nm (ε 1331 M^–1^ cm^–1^); ^1^H NMR (400.13 MHz, CDCl_3_): δ 7.53 (dd, *J* = 8.0 Hz, 1.9 Hz, 1H), 7.40–7.33 (m, 3H), 7.30–7.23 (m, 4H), 6.88 (dd, *J* = 8.0 Hz, 1.5 Hz, 1H), 4.15–4.09 (m, 1H), 3.36 (dd, *J* = 12.6 Hz, 2.7 Hz, 1H), 3.15 (dd, *J* = 12.6 Hz, 7.8 Hz, 1H), 2.92 (dd, *J* = 13.6 Hz, 4.9 Hz, 1H), 2.87 (dd, *J* = 13.6 Hz, 8.0 Hz, 1H); ^13^C{^1^H} NMR (100.61 MHz, CDCl_3_): δ 149.5, 149.0, 137.3, 129.9, 129.5, 129.0, 127.1, 119.4, 112.5, 106.8, 71.1, 49.1, 41.8; HRMS (ESI/TOF): Calcd for C_15_H_15_N_2_O_3_^–^ [M – H]^−^ 271.10882, found 271.10875.

#### 1-Allyl-4-nitrobenzene

A dry round-bottom flask fitted with a condenser was charged with 1-iodo-4-nitrobenzene (1.00 g, 4.00 mmol), CsF (1.52 g, 10.0 mmol), Pd(PPh_3_)_4_ (0.70 g, 15 mol %), THF (20 mL), and water (2 mL). The mixture was stirred at rt. under Ar for 30 min followed by addition of allylboronic acid pinacol ester (1.36 mL, 7.20 mmol) into THF (8 mL). The reaction mixture was refluxed (oil bath, 95 °C) for 22 h. After cooling to rt., the product mixture was evaporated onto celite and purified by silica-gel column chromatography (pet. ether). Concentration of the relevant fractions (*R*_f_ = 0.47 in pet. ether/EtOAc 8:2) gave the title compound (0.35 g, 54%) as a slightly yellow liquid. Spectroscopic data are in accordance with data reported in the literature.^43^

#### Attempt To Prepare 2-(4-Nitrobenzyl)oxirane under Anhydrous Conditions: Formation of 3-Methyl-2-(4-nitrophenyl)oxirane

An oven-dried round-bottom flask fitted with a condenser was charged with 1-iodo-4-nitrobenzene (996 mg, 4.00 mmol), CsF (2.127 g, 14.0 mmol), Pd(PPh_3_)_4_ (231 mg, 5 mol %), allylboronic acid pinacol ester (1.34 g, 8.00 mmol), and THF (50 mL). The reaction mixture was refluxed (oil bath, 80 °C) for 18 h. After cooling to rt., the product mixture was evaporated onto celite and purified by silica-gel flash chromatography (pet. ether). Concentration of the relevant fractions yielded a slightly yellow residue, which was dissolved in DCM (15 mL) and cooled (ice/water bath). *m*CPBA (632 mg, 2.82 mmol) was added, and the reaction mixture was stirred at 0 °C for 2 h and rt. for 22 h before quenching with 1:1 sat. NaHCO_3_:10% Na_2_S_2_O_3_ (20 mL). The phases were separated, and the aq. layer was extracted with DCM (3 × 15 mL). The combined organic phases were washed with 1:1 sat. NaHCO_3_:10% Na_2_S_2_O_3_ (20 mL), sat. aq. NaHCO_3_ (2 × 20 mL), water (20 mL), brine (20 mL), dried (MgSO_4_), filtered, and concentrated in vacuo to yield 3-methyl-2-(4-nitrophenyl)oxirane^44−46^ as a white solid (348 mg, 49% over two steps, mp. 79–81 °C; lit.^44^ mp 87–88 °C, lit.^45^ mp 90–92 °C). *R*_f_ = 0.60 in pet. Ether/EtOAc 6:4; IR (neat): ν_max_ 3109, 3073, 2977, 2933, 2855, 1601, 1513 cm^–1^; ^1^H NMR (400.13 MHz, CDCl_3_): δ 8.20 (d, *J* = 8.8 Hz, 2H), 7.42 (d, *J* = 8.8 Hz, 2H), 3.67 (d, *J* = 1.9 Hz, 1H), 3.02 (qd, *J* = 5.1 Hz, 1.9 Hz, 1H), 1.50 (d, *J* = 5.1 Hz, 3H); ^13^C{^1^H} NMR (100.61 MHz, CDCl_3_): δ 147.9, 145.5, 126.4, 123.9, 60.0, 58.5, 18.0.

#### Synthesis of 2-(4-Nitrobenzyl)oxirane

An oven-dried round-bottom flask charged with 1-allyl-4-nitrobenzene (0.35 g, 2.16 mmol) in anhydrous DCM (12 mL) was cooled (ice/water bath) and stirred for 5 min under Ar followed by addition of *m*CPBA (0.59 g, 2.63 mmol). The reaction mixture was stirred at 0 °C for 2 h, then at rt. for 15 h. More *m*CPBA (0.12 g, 0.54 mmol) was added and stirring continued for another 31 h before quenching the reaction with aq. NaOH solution (1 M, 10 mL). The phases were separated, and the aq. phase was extracted with DCM (3 × 15 mL). The combined organic phases were washed with water (20 mL), brine (20 mL), dried (MgSO_4_), filtered, and concentrated in vacuo. The product was isolated by silica-gel autoflash chromatography (pet. ether/EtOAc/DCM 93:2:5 → 40:55:5), and concentration of the relevant fractions (*R*_f_ = 0.26 (pet. ether/DCM 1:1)) yielded 2-(4-nitrobenzyl) oxirane as a yellow oily liquid (0.20 g, 52%). The spectroscopical data were in full accordance with the previously reported data.^47^

#### Synthesis of 3-(4-Nitrophenyl)-1-(phenylamino)propan-2-ol (**5c**)

A sealed tube was charged with aniline (0.15 mL, 1.67 mmol), 2-(4-nitrobenzyl)oxirane (0.30 g, 1.67 mmol), and methanol (0.5 mL). The reaction mixture was irradiated in a microwave reactor (170–180 °C, 9.5 bar, 300 W, 5 min ramping) for 5 min. The mixture was evaporated onto celite, and the crude product was isolated by silica-gel autoflash column chromatography (pet. Ether/EtOAc/DCM 90:5:5 *→* 45:50:5). Concentration of the relevant fractions (*R*_f_ = 0.20 in DCM/MeOH 99:1) yielded **5c** as a yellow solid (0.29 g, 64%, mp. 90–93 °C). IR (neat): ν_max_ 3326, 3054, 2919, 1598, 1506 cm^–1^; UV–vis: λ_max_ (EtOH) 248 nm (ε 15,067 M^–1^ cm^–1^); ^1^H NMR (400.13 MHz, CDCl_3_): δ 8.17 (d, *J* = 8.6 Hz, 2H), 7.42 (d, *J* = 8.6 Hz, 2H), 7.19 (dd, *J* = 8.5 Hz, 7.4 Hz, 2H), 6.77 (t, *J* = 7.4 Hz, 1H), 6.66 (d, *J* = 8.5 Hz, 2H), 4.16–4.10 (m, 1H), 3.32 (dd, *J* = 13.1 Hz, 3.4 Hz, 1H), 3.12 (dd, *J* = 13.1 Hz, 8.1 Hz, 1H), 2.99 (dd, *J* = 13.8 Hz, 4.7 Hz, 1H), 2.92 (dd, *J* = 13.8 Hz, 8.0 Hz, 1H); ^13^C{^1^H} NMR (100.61 MHz, CDCl_3_): δ 147.9, 146.9, 146.1, 130.3, 129.5, 123.8, 118.5, 113.5, 70.7, 49.9, 41.2; HRMS (ESI/TOF): calcd for C_15_H_15_N_2_O_3_^–^ [M – H]^−^ 271.10882, found 271.10881.

#### Synthesis of 1-Allyl-3-nitrobenzene

A round-bottom flask fitted with a condenser was charged with 1-iodo-3-nitrobenzene (1.99 g, 8.00 mmol), CsF (3.65 g, 24.0 mmol), Pd(PPh_3_)_4_ (1.38 g, 15 mol %), THF (55 mL), and water (15 mL). The mixture was stirred at rt. under Ar for 30 min, followed by addition of allylboronic acid pinacol ester (2.72 mL, 14.4 mmol). The reaction mixture was refluxed (oil bath, 95 °C) for 23 h. Pd(PPh_3_)_4_ (0.46 g, 5 mol %), CsF (1.22 g, 8.00 mmol), and allylboronic acid pinacol ester (0.75 mL, 4.00 mmol) were added. THF was removed under reduced pressure and replaced with dioxane (55 mL) followed by reflux (oil bath, 135 °C) for 28 h. After cooling to rt., the product mixture was evaporated onto celite and purified by silica-gel autoflash chromatography (pet. ether/DCM 95:5). Concentration of the relevant fractions (*R*_f_ = 0.53 in pet. Ether/EtOAc 8:2) gave the title compound (0.50 g, 38%) as a slightly yellow liquid. Spectroscopic data are in accordance with data reported in the literature.^43^

#### Synthesis of 2-(3-Nitrobenzyl)oxirane

A dry 25 mL round-bottom flask charged with 1-allyl-3-nitrobenzene (0.49 g, 3.00 mmol) in dry DCM (15 mL) was cooled (ice/water bath) and stirred for 5 min under Ar followed by addition of *m*CPBA (1.35 g, 6.02 mmol). The mixture was stirred at ambient temperature for 2 h, then at rt. for 26 h before being quenched with aq NaOH solution (1 M, 20 mL). The phases were separated, and the aq phase was extracted with DCM (3 × 15 mL). The combined organic phases were washed with water (20 mL) and brine (20 mL), dried (MgSO_4_), filtered, and concentrated in vacuo. The product was isolated by silica-gel autoflash chromatography (pet. ether/EtOAc/DCM 90:5:5 *→* 35:60:5) and concentration of the relevant fractions (*R*_f_ = 0.28 in pet. Ether/EtOAc/DCM 80:15:5) furnished the title compound^48,49^ as a yellowish oil (0.31 g, 58%). IR (neat): ν_max_ 3060, 2989, 2924, 1522, 1348 cm^–1^; ^1^H NMR (400.13 MHz, CDCl_3_): δ 8.13–8.11 (m, 2H), 7.62–7.60 (m, 1H), 7.51–7.48 (m, 1H), 3.21–3.17 (m, 1H), 3.06 (dd, *J* = 14.8 Hz, 4.4 Hz, 1H), 2.92 (dd, *J* = 14.8 Hz, 6.3 Hz, 1H), 2.85–2.83 (m, 1H), 2,55 (dd, *J* = 4.8 Hz, 2.6 Hz, 1H); ^13^C{^1^H} NMR (100.61 MHz, CDCl_3_): δ 148.5, 139.3, 135.4, 129.6, 124.0, 122.0, 51.8, 46.8, 38.3.

#### Synthesis of 3-(3-Nitrophenyl)-1-(phenylamino)propan-2-ol (**5d**)

A sealed reactor tube was charged with aniline (0.09 mL, 92 mg, 1.00 mmol), 2-(3-nitrobenzyl)oxirane (0.18 g, 1.00 mmol), and MeOH (0.5 mL). The reaction mixture was irradiated in a microwave reactor (170–180 °C, 9.5 bar, 300 W, 5 min ramping) for 5 min. The mixture was evaporated onto celite, and the product was isolated by silica-gel autoflash column chromatography (pet. Ether/EtOAc/DCM 90:5:5 *→* 45:50:5). Concentration of the relevant fractions (*R*_f_ = 0.52 in DCM/MeOH 95:5) gave **5d** as a brown oily wax (0.25 g, 91%). IR (neat): ν_max_ 3393, 3352, 3053, 3024, 2920, 1602 cm^–1^; UV–vis: λ_max_ (EtOH) 248 nm (ε 17,019 M^–1^ cm^–1^). ^1^H NMR (400.13 MHz, CDCl_3_): δ 8.14–8.13 (m, 1H), 8.10 (ddd, *J* = 8.2 Hz, 2.2 Hz, 1.0 Hz, 1H), 7.60–7.58 (m, 1H), 7.50–7.46 (m, 1H), 7.19 (dd, *J* = 8.5 Hz, 7.4 Hz, 2H), 6.76 (tt, *J* = 7.4 Hz, 1.0 Hz, 1H), 6.64 (dd, *J* = 8.5 Hz, 1.0 Hz, 2H), 4.13–4.07 (m, 1H), 3.32 (dd, *J* = 13.1 Hz, 3.4 Hz, 1H), 3.11 (dd, *J* = 13.1 Hz, 8.2 Hz, 1H), 2.98 (dd, *J* = 14.0 Hz, 4.6 Hz, 1H), 2.90 (dd, *J* = 14.0 Hz, 8.2 Hz, 1H); ^13^C{^1^H} NMR (100.61 MHz, CDCl_3_): δ 148.5, 148.0, 140.3, 135.8, 129.49, 129.48, 124.3, 121.8, 118.4, 113.5, 70.7, 49.9, 40.9; HRMS (ESI/TOF): calcd for C_15_H_15_N_2_O_3_^–^ [M – H]^−^ 271.10882, found 271.10869.

#### General Procedure for the Photodecomposition of **5a**–**5d**

A solution of the appropriate compound (≈0.10 mmol) in ACN was added to a photochemical Pyrex reactor containing distilled water at the appropriate pH (basic solutions were adjusted to correct pH with 1 M NaOH and acidic solutions were adjusted to correct pH with 1 M HCl) to a concentration of ≈0.7 mM and a total volume of either 75 or 150 mL (ACN/water 7:3), depending on the reactor size. The reaction vessel was either purged with N_2_ during the reaction or left open to air. The reaction mixture was photolyzed with a 125 W medium-pressure mercury lamp. After completion, the reaction mixture was transferred to a separatory funnel, saturated with NaCl, and extracted with EtOAc (3 × 50 mL). The aqueous phase was then adjusted to pH ≈ 2 with HCl (1 M), and the aqueous layer was extracted again with EtOAc (3 × 50 mL). The combined organic fractions were dried (MgSO_4_), filtered, and concentrated in vacuo on a rotary evaporator to yield a residue which was analyzed by ^1^H NMR.

#### Photolysis of Phenylacetaldehyde

A solution of phenylacetaldehyde (110 μL, 11.3 mg, 0.094 mmol) in ACN (45 mL) was added to a photochemical Pyrex reactor containing distilled water at pH 13. The reaction mixture was photolyzed with a 125 W medium-pressure mercury-vapor lamp for 2 h open to air. The resulting reaction mixture was extracted with DCM (3 × 20 mL). The aqueous phase was then adjusted to pH ≈ 2 with HCl (1 M), and the aqueous layer was extracted again with DCM (3 × 20 mL). The combined organic fractions were dried (MgSO_4_), filtered, and concentrated in vacuo on a rotary evaporator to yield a residue which was analyzed by ^1^H NMR.

#### Synthesis of Octyltriphenylphosphonium Bromide

A solution of triphenylphosphine (1.570 g, 6.00 mmol) and octyl bromide (1.14 mL, 1.275 g, 6.60 mmol) in toluene (20 mL) was refluxed (oil bath, 135 °C) for 4 d. An oily fraction was formed, and when the reaction mixture had reached rt., toluene was decanted off. The residue was rinsed with toluene (3 × 10 mL) to remove excess octyl bromide, and this gave the title compound as a colorless syrup (2.70 g, 99%). IR (neat): ν_max_ 3390, 3051, 2923, 2853, 1586, 1436 cm^–1^; ^1^H NMR (400.13 MHz, CDCl_3_): δ 7.90–7.84 (m, 6H), 7.81–7.76 (m, 3H), 7.72–7.67 (m, 6H), 3.90–3.83 (m, 2H), 1.64–1.61 (m, 4H), 1.25–1.19 (m, 10H), 0.83 (t, *J* = 6.9 Hz, 3H); ^13^C{^1^H} NMR (100.61 MHz, CDCl_3_): δ 135.0 (d, *J* = 3.0 Hz), 133.9 (d, *J* = 10.0 Hz), 130.6 (d, *J* = 12.5 Hz), 118.7 (d, *J* = 85.7 Hz), 31.8, 30.5 (d, *J* = 15.4 Hz), 29.4, 29.0, 23.2, 22.8 (d, *J* = 4.5 Hz), 22.7, 14.2; ^31^P NMR (161.98 MHz, CDCl_3_): δ 24.6; HRMS (ESI/TOF): calcd for C_26_H_32_P^+^ [M – Br]^+^ 375.22306, found 375.22380.

#### Synthesis of 1-Phenyldec-2-ene

To a stirred solution of octyltriphenylphosphonium bromide (2.70 g, 5.92 mmol) in dry THF (30 mL) was added sodium hydride as a 60% suspension in mineral oil (237 mg, 5.92 mmol). After 2 h of stirring at rt., the solution was cooled (ice/water bath), and phenylacetaldehyde (0.69 mL, 745 mg, 5.92 mmol) was added. The reaction mixture was stirred for 1 h at bath temperature and then for 48 h at rt. THF was removed under reduced pressure, water (20 mL) was added, and the hydrolysate was extracted with DCM (3 × 15 mL). The combined organic layers were concentrated onto celite, and the title compound was isolated by silica-gel flash chromatography (pet. ether). Concentration of the relevant fractions (*R*_f_ = 0.56 in pet. ether) yielded a mixture of *Z*:*E* isomers of the title compounds as a colorless liquid (617 mg, 48%). IR (neat): ν_max_ 3011, 2923, 2853, 1602, 1453 cm^–1^; ^1^H NMR (400.13 MHz, CDCl_3_): δ 7.31–7.27 (m, 2H), 7.21–7.17 (m, 3H), 5.59–5.49 (m, 2H), 3.41 (d, *J* = 6.0 Hz, 2H), 2.18–2.13 (m, 2H), 1.42–1.28 (m, 10H), 0.89 (t, *J* = 6.9 Hz, 3H); ^13^C{^1^H} NMR (100.61 MHz, CDCl_3_): δ 141.4, 131.2, 128.52, 128.49, 128.1, 125.9, 33.6, 32.0, 29.9, 29.5, 29.4, 27.4, 22.8, 14.3; HRMS (EI/TOF); 216, 117, 104 (100), 91 calcd for C_16_H_24_^+•^ [M]^+•^ 216.18725, found 216.18699. The proton spectrum showed a weak doublet at 3.34 ppm. The ratio between this signal and the doublet at 3.41 ppm was 6:94.

#### Synthesis of *cis*-2-Benzyl-3-heptyloxirane (**6**)

A stirred solution of (*Z*)-dec-2-enylbenzene/(*E*)-dec-2-enylbenzene 94:6 (616 mg, 2.85 mmol) in DCM (10 mL) under Ar was cooled (ice/water bath) followed by addition of *m*CPBA (766 mg, 3.42 mmol). The reaction mixture was stirred at 0 °C for 2 h and rt. for 22 h, before quenching with 1:1 sat. NaHCO_3_:10% Na_2_S_2_O_3_ (20 mL). The phases were separated, and the aq. layer was extracted with DCM (3 × 15 mL). The combined organic phases were washed with 1:1 sat. NaHCO_3_:10% Na_2_S_2_O_3_ (20 mL), sat. aq. NaHCO_3_ (2 × 20 mL), water (20 mL), and brine (20 mL), dried (MgSO_4_), filtered, and concentrated in vacuo to afford **6** (456 mg, 92%) as a colorless liquid. *R*_f_ = 0.33 in pet. ether/EtOAc 95:5; IR (neat): ν_max_ 3028, 2955, 2923, 2854, 1604 cm^–1^; ^1^H NMR (400.13 MHz, CDCl_3_): δ 7.35–7.30 (m, 2H), 7.28–7.22 (m, 3H), 3.17 (td, *J* = 6.2 Hz, 4.2 Hz, 1H), 3.00 (ddd, *J* = 6.7 Hz, 5.5 Hz, 4.2 Hz, 1H), 2.92 (dd, *J* = 14.7 Hz, 6.4 Hz, 1H), 2.81 (dd, *J* = 14.7 Hz, 6.2 Hz, 1H), 1.70–1.60 (m, 2H), 1.58–1.23 (m, 10H), 0.89 (t, *J* = 7.0 Hz, 3H); ^13^C{^1^H} NMR (100.61 MHz, CDCl_3_): δ 138.2, 128.9, 128.7, 126.7, 57.6, 57.5, 34.5, 31.9, 29.7, 29.4, 28.2, 26.8, 22.8, 14.2; HRMS (ESI/TOF): calcd for C_16_H_24_ONa^+^ [M + Na]^+^ 255.17139, found 255.17201.

#### Synthesis of 3-Azido-1-phenyldecan-2-ol (**7a**)

To a stirred solution of *cis*-2-benzyl-3-heptyloxirane (**6**) (654 mg, 2.81 mmol) in MeOH (6.3 mL) and water (0.7 mL) were added NaN_3_ (548 mg, 8.43 mmol) and NH_4_Cl (301 mg, 5.62 mmol) at rt. The reaction mixture was stirred at 50 °C for 48 h. MeOH and water were removed under reduced pressure and the residue was purified by silica-gel flash chromatography (pet. Ether/EtOAc 95:5 *→* 90:10) to yield a 7:3 mixture of regioisomers of the title compound as a colorless oily liquid (624 mg, 81%). *R*_f_ = 0.34 in pet. ether/EtOAc 9:1; IR (neat): ν_max_ 3438, 2039, 2925, 2856, 2101, 1604, 1455 cm^–1^; ^1^H NMR (400.13 MHz, CDCl_3_): δ 7.35–7.31 (m, in a ratio 7:3, 2H, **7a**/**7b**), 7.28–7.22 (m, in a ratio 7:3, 3H, **7a**/**7b**), 3.84–3.79 and 3.54–3.50 (2 × m, in a ratio of 7:3, 1H, **7a**/**7b**), 3.49–3.45 (m, 1H, b), 3.23–3.19 (m, 1H, a), 3.03 (dd, *J* = 13.8 Hz, 6.1 Hz, 1H, b), 2.94 (dd, *J* = 13.8 Hz, 8.3 Hz, 1H, b), 2.90–2.81 (m, 2H, a), 1.77–1.61 (m, 2H, a/b), 1.60–1.23 (m, in a ratio 7:3, 10H, **7a**/**7b**), 0.90–0.86 (m, in a ratio 7:3, 3H, **7a**/**7b**); ^13^C{^1^H} NMR (100.61 MHz, CDCl_3_): δ 137.75 (**7a**), 137.67 (**7b**), 129.49 (**7a**), 129.47 (**7b**), 128.9 (**7a**), 128.8 (**7b**), 127.0 (**7b**), 126.9 (**7a**), 74.6 (**7a**), 72.7 (**7b**), 68.1 (**7b**), 65.6 (**7a**), 41.0 (**7a**), 37.5 (**7b**), 34.7 (**7b**), 31.90 (**7b**), 31.88 (**7a**), 31.0 (**7a**), 29.6 (**7b**), 29.5 (**7a**), 29.31 (**7b**), 29.26 (**7a**), 26.4 (**7a**), 25.8 (**7b**), 22.8 (**7a**/**7b**), 14.2 (**7a**/**7b**); HRMS (ESI/TOF): calcd for C_16_H_25_N_3_Ona^+^ [M + Na]^+^ 298.18843, found 298.18898.

#### Synthesis of 3-Amino-1-phenyldecan-2-ol (**8a**)

A mixture of regioisomers **7a**:**7b** (7:3) (620 mg, 2.25 mmol) dissolved in EtOAc (7 mL) was added 10% Pd/C (38 mg, 10 mol %). The reaction mixture was purged with hydrogen gas (1 atm, balloon) for 10 min before the flask was sealed and left stirring under a hydrogen atmosphere for 24 h. Pd/C was removed by filtering through a 0.45 μm PP syringe filter, and concentration of the filtrate yielded a 7:3 mixture of **8a** and **8b** (543 mg, 97%). *R*_f_ = 0.09 in DCM/MeOH 99:1; IR (neat): ν_max_ 3287, 3112, 3029, 2919 2852, 1602 cm^–1^; ^1^H NMR (400.13 MHz, CDCl_3_): δ 7.35–7.29 (m, in a ratio 7:3, 2H, **8a**/**8b**), 7.25–7.18 (m, in a ratio 7:3, 3H, **8a**/**8b**), 3.58–3.53 (m, 0.7H, **8a**), 3.37–3.33 (m, 0.3H, **8b**), 2.95–2.86 (m, 0.6H, **8b**), 2.84 (dd, *J* = 13.7 Hz, 4.2 Hz, 0.7H, **8a**), 2.72 (dd, *J* = 13.7 Hz, 8.1 Hz, 0.7H, **8a**), 2.64–2.60 (m, 0.7H, **8a**), 2.48 (dd, *J* = 13.0 Hz, 9.1 Hz, 0.3H, **8b**), 1.60–1.46 (m, in a ratio 7:3, 2H, **8a**/**8b**), 1.43–1.27 (m, in a ratio 7:3, 10H, **8a**/**8b**), 0.88 (t, *J* = 6.8 Hz, in a ratio 7:3, 3H, **8a**/**8b**); ^13^C{^1^H} NMR (100.61 MHz, CDCl_3_): δ 139.3 (**8b**), 139.0 (**8a**), 129.5 (**8a**), 129.4 (**8b**), 128.7 (**8b**), 128.6 (**8a**), 126.5 (**8b**), 126.4 (**8a**), 74.8 (**8a**), 73.6 (**8b**), 56.8 (**8b**), 54.7 (**8a**), 41.3 (**8b**), 41.1 (**8a**), 34.8(3) (**8b**), 34.8(1) (**8a**), 31.9(9) (**8b**), 31.9(6) (**8a**), 29.9 (**8b**), 29.8 (**8a**), 29.4(4) (**8b**), 29.4(0) (**8a**), 26.4 (**8a**), 26.0 (**8b**), 22.8(1) (**8b**), 22.79 (**8a**), 14.25 (**8b**), 14.24 (**8a**); HRMS (ESI/TOF): calcd for C_16_H_28_NO^+^ [M + H]^+^ 250.21599, found 250.21677.

#### Synthesis of 3-((4-Nitrophenyl)amino)-1-phenyldecan-2-ol (**9a**)

A solution of amine **8** (249 mg, 1.00 mmol), 1-fluoro-4-nitrobenzene (155 mg, 1.10 mmol), and DIPEA (0.52 mL, 3.00 mmol) in DMF (2 mL) was stirred at 80 °C under Ar for 24 h. The product was isolated from a 7:3 mixture of regioisomers **9a** and **9b** by two consecutive purifications by silica-gel column chromatography (pet. ether/DCM 1:1 *→* 0:1 and pet. Ether/DCM 3:7) and concentration of the relevant fractions (*R*_f_ = 0.11 in pet. ether/DCM 3:7) yielded **9a** as a yellow waxy solid (44 mg, 12%). The undesired amino alcohol **9b** was not isolated. IR (neat): ν_max_ 3500, 3398, 3061, 2925, 2855, 1597 cm^–1^; ^1^H NMR (400.13 MHz, CDCl_3_): δ 8.08 (d, *J* = 9.2 Hz, 2H), 7.34–7.30 (m, 2H), 7.28–7.24 (m, 1H), 7.16–7.14 (m, 2H), 6.51 (d, *J* = 9.2 Hz, 2H), 4.91 (d, *J* = 9.6 Hz, NH), 4.02 (t, *J* = 6.6 Hz, 1H), 3.49–3.43 (m, 1H), 2.82 (d, *J* = 6.9 Hz, 2H), 1.78 (bs, OH), 1.72–1.57 (m, 2H), 1.35–1.24 (m, 10H), 0.86 (t, *J* = 6.9 Hz, 3H); ^13^C{^1^H} NMR (100.61 MHz, CDCl_3_): δ 153.7, 137.8, 137.5, 129.5, 129.0, 127.1, 126.8, 111.3, 73.5, 55.6, 41.2, 32.8, 31.9, 29.7, 29.3, 26.4, 22.7, 14.2; HRMS (ESI/TOF): calcd for C_22_H_30_N_2_O_3_Na^+^ [M + Na]^+^ 393.21431, found 393.21499.

#### Photolysis of 3-((4-Nitrophenyl)amino)-1-phenyl-decan-2-ol (**9a**)

A solution of compound **9a** (23.6 mg, 0.064 mmol) in ACN/water 7:3 (150 mL) was photolysed in a 150 mL (0.42 mM) photochemical Pyrex reactor according to the general procedure with a 125 W medium-pressure mercury lamp for 4 h at pH 11. The aqueous phase was extracted with DCM (3 × 50 mL). pH of the aqueous phase was adjusted to ≈2 with HCl (1 M) and extracted with DCM (3 × 50 mL). The combined organic fractions were dried (MgSO_4_), filtered, and concentrated. The resulting residue was used for further analysis.

#### Synthesis of *N*-(3,5-Dichloro-2-fluorophenyl)acetamide

A solution of 3,5-dichloro-2-fluoroaniline (216 mg, 1.20 mmol) in anhydr. DCM (4 mL) was cooled (ice/water bath) followed by dropwise addition of acetyl chloride (140 μL, 1.92 mmol) and Et_3_N (270 μL, 1.92 mmol) over a period of 5 min. The reaction mixture was stirred at ambient temperature for 30 min and then at rt. for another 30 min, before quenching with water (10 mL) and sat. aq. NaHCO_3_ solution (10 mL). The phases were separated, the aq. layer was extracted with DCM (3 × 10 mL), the combined organic layers were concentrated, and the product was isolated by silica-gel column chromatography (pet. Ether/EtOAc 8:2). Concentration of the relevant fractions (*R*_f_ = 0.24 in pet. ether/EtOAc 8:2) furnished the title compound as a white solid (242 mg, 91%, mp. 168–169 °C). IR (neat): ν_max_ 3293, 3252, 3181, 3116, 3083, 3046, 2990, 2923, 1678, 1606 cm^–1^; ^1^H NMR (400.13 MHz, CDCl_3_): δ 8.35 (dd, *J* = 5.9 Hz, 2.0 Hz, 1H), 7.33 (bs, NH), 7.11 (dd, *J* = 6.2 Hz, 2.6 Hz, 1H), 2.24 (s, 3H); ^13^C{^1^H} NMR (100.61 MHz, CDCl_3_): δ 168.3, 145.7, 130.1 (d, *J* = 4.6 Hz), 128.4 (d, *J* = 11.0 Hz), 124.5, 121.3 (d, *J* = 17.7 Hz), 119.9, 24.9; ^19^F NMR (376.46 MHz, CDCl_3_): δ −135.2; HRMS (ESI/TOF): calcd for C_8_H_5_NO^35^Cl_2_F^–^ [M-H]^−^ 219.97432, found 219.97361.

#### Synthesis of *N*-(3,5-Dichloro-2-fluoronitrophenyl)acetamide

A stirred solution of *N*-(3,5-dichloro-2-fluorophenyl)acetamide (520 mg, 2.34 mmol)in conc. sulfuric acid (6.5 mL) was cooled to −10 °C (ice/salt bath) followed by dropwise addition of an ice cold mixture of conc. sulfuric acid (6.5 mL) and 65% nitric acid (8.4 mL) over a period of 15 min. The reaction mixture was stirred at ambient temperature for 1 h and then poured into a beaker with ice. DCM (20 mL) was added, the phases were separated, and the aq. layer was extracted with DCM (3 × 15 mL). The combined organic layers were washed with water (20 mL), dried (MgSO_4_), filtered, and concentrated under reduced pressure to yield a 62:38 mixture of *p*- and *o*-nitrated products as an off-white solid (500 mg, 80%). *R*_f_ = 0.47 (*p*-NO_2_) and 0.51 (*o*-NO_2_) in pet. Ether/EtOAc 6:4; IR (neat): ν_max_ 3263, 3194, 3112, 3073, 1706, 1682 cm^–1^; ^1^H NMR (400.13 MHz, CDCl_3_): δ 8.63 (d, *J* = 6.7 Hz, 1H, *p*-NO_2_), 7.52 (d, *J* = 6.3 Hz, 1H, *o*-NO_2_), 7.60 (bs, NH, *p*-NO_2_), 7.42 (bs, NH, *o*-NO_2_), 2.28 (s, 3H, *p*-NO_2_), 2.21 (s, 3H, *o*-NO_2_); ^13^C{^1^H} NMR (100.61 MHz, CDCl_3_): δ 168.9 (*p*-NO_2_), 168.7 (*o*-NO_2_), 152.4 (d, *J* = 256.7 Hz, *o*-NO_2_), 146.4 (d, *J* = 248.3 Hz, *p*-NO_2_), 144.5 (*o*-NO_2_), 143.5 (*p*-NO_2_), 129.8 (*o*-NO_2_), 129.5 (d, *J* = 10.8 Hz, *p*-NO_2_), 125.9 (d, *J* = 18.5 Hz, *o*-NO_2_), 122.4 (d, *J* = 4.6 Hz, *p*-NO_2_), 122.1 (d, *J* = 5.1 Hz, *o*-NO_2_), 121.1 (d, *J* = 17.8 Hz, *o*-NO_2_), 120.2 (*p*-NO_2_), 115.1 (d, *J* = 21.8 Hz, *p*-NO_2_), 24.9 (*p*-NO_2_), 23.2 (*o*-NO_2_); ^19^F NMR (376.46 MHz, CDCl_3_): δ −129.7 (*p*-NO_2_), −114.7 (*o*-NO_2_); HRMS (ESI/TOF): calcd for C_8_H_4_N_2_O_3_^35^Cl_2_F^–^ [M – H]^−^ 264.958940, found 264.95792.

#### Synthesis of 3,5-Dichloro-2-fluoro-4-nitroaniline (**10**)

A mixture of *o*- and *p*-nitro isomers of acetamide (500 mg, 1.87 mmol) from the previous synthesis was dissolved in MeOH (25 mL) and 37% hydrochloric acid (3 mL) followed by stirring at 60 °C for 6 h. The volatiles were removed under reduced pressure and the product was isolated by silica-gel autoflash chromatography (pet. ether/EtOAc 9:1 *→* 7:3). Concentration of the relevant fractions (*R*_f_ = 0.17 in pet. ether/EtOAc 8:2) yielded the title compound **10** (284 mg, 67%, mp. 142–144 °C) as a yellow crystalline solid. IR (neat): ν_max_ 3498, 3394, 3205, 3055, 1623 cm^–1^; ^1^H NMR (400.13 MHz, CDCl_3_): δ 6.75 (d, *J* = 7.5 Hz, 1H), 4.27 (bs, NH_2_); ^13^C{^1^H} NMR (100.61 MHz, CDCl_3_): δ 145.1 (d, *J* = 244.9 Hz), 139.2, 137.8 (d, *J* = 13.4 Hz), 122.6 (d, *J* = 4.2 Hz), 116.0 (d, *J* = 20.5 Hz), 114.1 (d, *J* = 3.9 Hz); ^19^F NMR (376.46 MHz, CDCl_3_): δ −135.1; HRMS (ESI/TOF): Calcd for C_8_H_12_^35^Cl_2_FN_2_O_4_^+^ [M + 2CH_3_OH + H]^+^ 289.01472, found 289.01543.

#### Synthesis of 2-(2,6-Difluorobenzyl)oxirane (**11**)

A 35 mL reactor tube charged with 2-bromo-1,3-difluorobenzene (965 mg, 5.00 mmol), allylboronic acid pinacol ester (1008 mg, 6.00 mmol), Pd(PPh_3_)_4_ (289 mg, 5 mol %), CsF (2.70 g, 17.5 mmol), and anhydr. THF (15 mL) was purged with argon gas and irradiated at 120 °C for 1 h in a microwave reactor. The resulting slurry was filtered with the aid of DCM (100 mL), and the filtrate was evaporated onto celite. 2-Allyl-1,3-difluorobenzene was isolated by silica-gel flash chromatography (pet. ether) and the relevant fractions (*R*_f_ 0.65, pet. ether) were concentrated until 5 mL pet. ether remained. Dry DCM (15 mL) was added, and the solution was cooled to 0 °C (ice/water) followed by addition of *m*CPBA (1.35 g, 6.00 mmol). The reaction mixture was stirred at ambient temperature for 2 h and then rt. for 26 h before quenching with 1:1 sat. aq. NaHCO_3_:10% Na_2_S_2_O_3_ solution (30 mL). The phases were separated, and the aq. layer was extracted with DCM (3 × 15 mL). The combined organic layers were washed with 1:1 sat. aq. 1:1 sat. NaHCO_3_:10% Na_2_S_2_O_3_ (30 mL), sat. aq. NaHCO_3_ solution (30 mL), and water (30 mL), dried (MgSO_4_), filtered, and concentrated to yield **11** as a colorless liquid (340 mg, 40% over two steps). *R*_f_ = 0.43 in pet. ether/DCM 5:5; IR (neat): ν_max_ 3056, 2998, 2928, 1626, 1589, 1468 cm^–1^; ^1^H NMR (400.13 MHz, CDCl_3_): δ 7.24–7.16 (m, 1H), 6.90–6.86 (m, 2H), 3.19–3.10 (m, 2H), 2.82 (dd, *J* = 14.0 Hz, 5.8 Hz, 1H), 2.77–2.75 (m, 1H), 2.56 (dd, *J* = 4.9 Hz, 2.4 Hz); ^13^C{^1^H} NMR (100.61 MHz, CDCl_3_): δ 161.9 (dd, *J* = 247.4 Hz, 8.5 Hz), 128.6 (t, *J* = 10.2 Hz), 112.6 (t, *J* = 20.5 Hz), 111.3 (dd, *J* = 19.0 Hz, 6.9 Hz), 50.8, 47.1, 25.6 (t, *J* = 2.0 Hz); ^19^F NMR (376.46 MHz, CDCl_3_): δ −114.8; HRMS (ESI/TOF): calcd for C_9_H_9_OF_2_^+^ [M + H]^+^ 171.06160, found 171.06276.

#### 2-(2,6-Difluoro-3-nitrobenzyl)oxirane (**12**)

To a stirred solution of 2-allyl-1,3-difluoro-4-nitrobenzene (194 mg, 0.97 mmol) at 0 °C was added *m*CPBA (425 mg, 1.94 mmol). The reaction mixture was stirred for 2 h at 0 °C and 5 d at rt. followed by addition of a 1:1 sat. NaHCO_3_:10% Na_2_S_2_O_3_ (30 mL). The phases were separated, and the aqueous layer was extracted with DCM (3 × 15 mL). The combined organic phases were washed with 1:1 sat. NaHCO_3_:10% Na_2_S_2_O_3_ (30 mL), sat. aq. NaHCO_3_ (30 mL), and water (30 mL), dried (MgSO_4_), filtered, and concentrated under reduced pressure to yield 2-(2,6-difluoro-3-nitrobenzyl)oxirane (**12**) (*R*_f_ = 0.53 in DCM) as a slightly yellow oily liquid (183 mg, 88%). IR (neat): ν_max_ 3104, 3000, 2926, 1728, 1624 cm^–1^; ^1^H NMR (400.13 MHz, CDCl_3_): δ 8.05 (ddd, *J* = 9.2 Hz, 8.5 Hz, 5.7 Hz, 1H), 7.07–7.02 (m, 1H), 3.22–3.17 (m, 1H), 3.16–3.11 (m, 1H), 3.03–2.97 (m, 1H), 2.81–2.79 (m, 1H), 2.56 (dd, *J* = 4.8 Hz, 2.5 Hz, 1H); ^13^C{^1^H} NMR (100.61 MHz, CDCl_3_): δ 164.5 (dd, *J* = 259 Hz, 8 Hz), 155.5 (dd, *J* = 266 Hz, 9 Hz), 134.5, 126.1 (dd, *J* = 11 Hz, 1 Hz), 115.8 (dd, *J* = 22 Hz, 20 Hz), 111.8 (dd, *J* = 25 Hz, 4 Hz), 50.1, 46.9, 25.8 (t, *J* = 2 Hz); ^19^F NMR (376.46 MHz, CDCl_3_): δ −101.5 (d, *J* = 13.6 Hz), −115.7 (d, *J* = 13.6 Hz); HRMS (EI/TOF): 185, 172 (100), 142, 126, 119 Calcd for C_7_H_4_F_2_NO_2_^+•^ [M-C_2_H_3_O]^+•^ 172.02000, found 172.02080.

#### Synthesis of 1-Allyl-4-chlorobenzene

A mixture of 4-chloro-1-iodobenzene (954 mg, 4.00 mmol), Pd(PPh_3_)_4_ (46 mg, 1 mol %), CsF (2127 mg, 14.0 mmol), and allyl boronate pinacol ester (1.51 mL, 8.00 mmol) in anhydr. THF (50 mL) was refluxed (oil bath, 70 °C) under Ar for 16 h. The reaction mixture was cooled to rt., and pet. ether (20 mL) and water (20 mL) were added. The phases were separated, and the aq layer was extracted with pet. ether (3 × 15 mL). The combined organic phases were concentrated under reduced pressure onto celite, and the product was isolated by silica-gel column chromatography (pet. ether). Concentration of the relevant fractions (*R*_f_ = 0.51 in pet. ether) furnished the title compound as a colorless liquid (471 mg, 77%). Spectroscopic data are in accordance with previously reported data in the literature.^43^

#### Synthesis of 2-(4-Chlorobenzyl)oxirane (**13**)

A stirred solution of 1-allyl-4-chlorobenzene (450 mg, 2.95 mmol) in anhydrous DCM (8 mL) under Ar was cooled (ice/water bath) followed by addition of *m*CPBA (804 mg, 3.59 mmol). The reaction mixture was stirred at ambient temperature for 2 h and rt. for 22 h, before being quenched with 1:1 sat. NaHCO_3_:10% Na_2_S_2_O_3_ (20 mL). The phases were separated, and the aq. layer was extracted with DCM (3 × 15 mL). The combined organic phases were washed with 1:1 sat. NaHCO_3_:10% Na_2_S_2_O_3_ (20 mL), sat. aq. NaHCO_3_ (2 × 20 mL), water (20 mL), and brine (20 mL) and then dried (MgSO_4_), filtered, and concentrated in vacuo to yield **11** as a colorless liquid (456 mg, 92%). Spectroscopic data are in accordance with previously reported data in the literature.^50^

#### Lewis Acid-Promoted Epoxide Ring Opening General Procedure for the Preparation of **14e**–**14k**

Lithium perchlorate was dried under vacuum for 1 h and dissolved in dry diethyl ether to a 5 M solution. Aniline (∼0.2 M) and epoxide (1.0 equiv) were added, and the reaction mixture was stirred at 40 °C under Ar. DCM and water were added, the phases were separated, and the aqueous layer was extracted with DCM (3 × 10 mL). The combined organic phases were evaporated on celite and subjected to silica-gel flash chromatography (pet. ether/DCM 3:7). Concentration of the relevant fractions gave the corresponding propan-2-ol derivative essentially pure according to ^1^H-NMR analysis.

#### Synthesis of 1-(3,5-Dichloro-2-fluoro-4-nitrophenyl)-amino-3-(2,6-difluorophenyl)propan-2-ol (**14e**)

A dry round-bottom flask was charged with lithium perchlorate (2.52 g, 23.7 mmol) and diethyl ether (5 mL). The solution was stirred for 30 min followed by addition of 3,5-dichloro-2-fluoro-4-nitroaniline (260 mg, 1.16 mmol) and 2-(2,6-difluorobenzyl)oxirane (198 mg, 1.16 mmol). The reaction mixture was stirred at reflux (oil bath, 60 °C) for 3 d before DCM (10 mL) was added followed by dropwise addition of water (10 mL). The phases were separated, and the organic layer was extracted with DCM (3 × 10 mL). Isolation by silica-gel flash chromatography (pet. ether/DCM 1:1) and concentration of the relevant fractions (*R*_f_ = 0.16 in pet. ether/DCM 3:7) gave **14e** (91 mg, 20%, mp. 158–159 °C) and recovered 3,5-dichloro-2-fluoro-4-nitroaniline (89 mg, 34%). IR (neat): ν_max_ 3402, 3369, 2968, 2928, 1604 cm^–1^; ^1^H NMR (400.13 MHz, CD_3_CN): δ 7.32–7.24 (m, 1H), 7.00–6.93 (m, 1H), 6.83 (d, *J* = 7.5 Hz, 1H), 5.45 (bs, NH), 4.05–3.98 (m, 1H), 3.37–3.31 (m, 1H), 3.31 (d, *J* = 5.3 Hz, OH), 2.87–2.85 (m, 2H); ^13^C{^1^H} NMR (100.61 MHz, CD_3_CN): δ 162.8 (dd, *J* = 245 Hz, 9 Hz), 116.0 (d, *J* = 244 Hz), 141.3 (d, *J* = 12 Hz), 137.3, 129.5 (t, *J* = 10 Hz), 123.4 (d, *J* = 4 Hz), 115.1 (d, *J* = 20 Hz), 115.1 (t, *J* = 20 Hz), 112.1 (d, *J* = 26 Hz), 111.0 (*J* = 4 Hz), 69.8, 49.1, 28.7; ^19^F NMR (376.46 MHz, CD_3_CN): δ −115.7, −136.8; HRMS (ESI/TOF): calcd for C_15_H_10_N_2_O_3_^35^Cl_2_F_3_^–^ [M – H]^−^ 393.00316, found 393.00245.

#### Synthesis of 3-(4-Chlorophenyl)-1-((3,5-dichloro-2-fluoro-4-nitrophenyl)amino)propan-2-ol (**14f**)

3,5-Dichloro-2-fluoro-4-nitroaniline (77 mg, 0.34 mmol) and 2-(4-chlorobenzyl)oxirane (57 mg, 0.34 mmol) were reacted following the general procedure for 20 h. The target compound was isolated by two consecutive purifications by silica-gel flash chromatography (pet. ether/DCM 2:8 and pet. ether/EtOAc 7:3). The relevant fractions were concentrated, and pet. ether (2 mL) and EtOAc (3 drops) were added to the residue. The mixture was heated to 50 °C and the liquid was decanted off, leaving a yellow solid containing 80% pure product. This mixture was washed with a solution of pet. ether (10 mL) and EtOAc (15 drops) followed by a final rinse with EtOAc (10 mL) to yield aminol **14f** as a yellow crystalline solid (20 mg, 15%, mp. 148–149 °C). *R*_f_ = 0.46 in pet. ether/EtOAc 6:4; IR (neat): ν_max_ 3380, 3091, 2918, 2859, 1603 cm^–1^; ^1^H NMR (400.13 MHz, CD_3_CN): δ 7.31 (d, *J* = 8.5 Hz, 2H, ArH), 7.25 (d, *J* = 8.5 Hz, 2H, ArH), 6.79 (d, *J* = 7.5 Hz, 1H, ArH), 5,44 (bs, NH), 3.99–3.91 (m, 1H), 3.29 (ddd, *J* = 13.7 Hz, 6.3 Hz, 3.9 Hz, 1H), 3.19 (d, *J* = 5.1 Hz, OH), 3.17–3.11 (m, 1H), 2.82 (dd, *J* = 13.8 Hz, 4.9 Hz, 1H), 2.70 (dd, *J* = 13.8 Hz, 8.0 Hz, 1H); ^13^C{^1^H} NMR (100.61 MHz, CD_3_CN): δ 146.0 (d, *J* = 244 Hz), 141.3 (d, *J* = 12 Hz), 138.7, 137.3, 132.5, 132.2, 129.2, 123.5 (d, *J* = 4 Hz), 115.2 (d, *J* = 21 Hz), 111.0 (d, *J* = 4 Hz), 71.2, 49.1, 41.1; ^19^F NMR (376.46 MHz, CD_3_CN): δ −136.7; HRMS (ESI/TOF): calcd for C_15_H_11_^35^Cl_3_FN_2_O_3_^–^ [M – H]^−^ 390.98194, found 390.98148.

#### Synthesis of 1-(2,6-Difluorophenyl)amino-3-(4-nitrophenyl)propan-2-ol (**14g**)

2,6-Difluoroaniline (43 mg, 0.33 mmol) and 2-(4-nitrobenzyl)oxirane (59 mg, 0.33 mmol) were reacted according to the general procedure for 6 h to yield aminol **14g** as a white solid (31 mg, 30%, mp. 86–87 °C) along with 43% recovery of epoxide. *R*_f_ = 0.15 in DCM; IR (neat): ν_max_ 3308, 3080, 2946, 2886, 2855, 1600 cm^–1^; ^1^H NMR (400.13 MHz, CD_3_CN): δ 8.13 (d, *J* = 8.7 Hz, 2H), 7.43 (d, *J* = 8.7 Hz, 2H), 6.91–6.81 (m, 2H), 6.73–6.65 (m, 1H), 4.27 (bs, NH), 3.98–3.90 (m, 1H), 3.46–3.40 (m, 1H), 3.20–3.14 (m, 1H, overlapping with OH), 3.17 (d, *J* = 5.3 Hz, OH), 2.95 (dd, *J* = 13.7 Hz, 4.4 Hz, 1H), 2.80 (dd, *J* = 13.7 Hz, 8.4 Hz, 1H); ^13^C{^1^H} NMR (100.61 MHz, CD_3_CN): δ 154.3 (dd, *J* = 240 Hz, 8 Hz), 148.4, 147.6, 131.5, 127.0 (t, *J* = 14 Hz), 124.2, 118.5 (t, *J* = 10 Hz), 112.5 (dd, *J* = 16 Hz, 7 Hz), 71.9, 52.4 (t, *J* = 4 Hz), 41.7; ^19^F NMR (376.46 MHz, CD_3_CN): δ −130.0; HRMS (ESI/TOF): Calcd for C_15_H_15_F_2_N_2_O_3_^+^ [M + H]^+^ 309.10398, found 309.10467.

#### Synthesis of 1-(3,5-Dichloro-2-fluorophenyl)amino-3-(4-nitrophenyl)propan-2-ol (**14h**)

3,5-Dichloro-2-fluoro-aniline (59 mg, 0.33 mmol) and 2-(4-nitrobenzyl)oxirane (59 mg, 0.33 mmol) were reacted according to the general procedure for 20 h to yield aminol **14h** as a white solid (57 mg, 48%, mp. 113–114 °C) along with 32% recovery of epoxide. *R*_f_ = 0.31 in DCM; IR (neat): ν_max_ 3447, 3351, 3263, 3117, 3085, 2950, 2919, 2904, 2850, 1604 cm^–1^; ^1^H NMR (400.13 MHz, CD_3_CN): δ 8.13 (d, *J* = 8.8 Hz, 2H), 7.47 (d, *J* = 8.8 Hz, 2H), 6.68–6.64 (m, 2H), 4.92 (bs, NH), 4.03–3.96 (m, 1H), 3.28–3.22 (m, 1H, overlapping with OH), 3.24 (d, *J* = 5.1 Hz, OH), 3.12–3.05 (m, 1H), 2.97 (dd, *J* = 13.7 Hz, 4.4 Hz, 1H), 2.82 (dd, *J* = 13.7 Hz, 8.4 Hz, 1H); ^13^C{^1^H} NMR (100.61 MHz, CD_3_CN): δ 148.3, 147.6, 146.8 (d, *J* = 239 Hz), 139.9 (d, *J* = 12 Hz), 131.5, 130.5 (d, *J* = 4 Hz), 124.2, 121.3 (d, *J* = 16 Hz), 116.4 (d, *J* = 2 Hz), 111.5 (d, *J* = 3 Hz), 70.9, 49.5, 41.6; ^19^F NMR (376.46 MHz, CD_3_CN): δ −141.6; HRMS (ESI/TOF): Calcd for C_15_H_14_^35^Cl_2_FN_2_O_3_^+^ [M + H]^+^ 359.03545, found 359.03598.

#### Synthesis of 1-(3,5-Dichloro-2,4-difluorophenyl)-amino)-3-(4-nitrophenyl)propan-2-ol (**14i**)

3,5-Dichloro-2,4-difluoroaniline (65 mg, 0.33 mmol) and 2-(4-nitrobenzyl)oxirane (59 mg, 0.33 mmol) were reacted according to the general procedure for 18 h to yield aminol **14i** as a white solid (59 mg, 48%, mp. 124–125 °C) along with 24% recovered epoxide. *R*_f_ = 0.29 in DCM; IR (neat): ν_max_ 3386, 3293, 3116, 3080, 2927, 2850, 1601 cm^–1^; ^1^H NMR (400.13 MHz, CD_3_CN): δ 8.14 (d, *J* = 8.8 Hz, 2H), 7.48 (d, *J* = 8.8 Hz, 2H), 6.76 (dd, *J* = 8.5 Hz, 7.2 Hz, 1H), 4.72 (bs, NH), 4.04–3.96 (m, 1H), 3.26–3.21 (m, 1H, overlapping with OH), 3.21 (d, *J* = 5.0 Hz, OH), 3.10–3.04 (m, 1H), 2.97 (dd, *J* = 13.7 Hz, 4.4 Hz, 1H), 2.82 (dd, *J* = 13.7 Hz, 8.4 Hz, 1H); ^13^C{^1^H} NMR (100.61 MHz, CD_3_CN): δ 148.3, 147.7, 146.9 (dd, *J* = 242 Hz, 2 Hz), 146.3 (dd, *J* = 237 Hz, 2 Hz), 135.7 (dd, *J* = 12 Hz, 3 Hz), 131.5, 124.2, 117.3 (dd, *J* = 18 Hz, 4 Hz), 111.2 (dd, *J* = 22 Hz, 20 Hz), 111.1 (d, *J* = 4 Hz), 70.9, 49.8, 41.6; ^19^F NMR (376.46 MHz, CD_3_CN): δ −134.2 (d, *J* = 4.4 Hz), −136.9 (d, *J* = 4.4 Hz); HRMS (ESI/TOF): calcd for C_15_H_13_^35^Cl_2_F_2_N_2_O_3_^+^ [M + H]^+^ 377.02603, found 377.02648.

#### Synthesis of 3-(2,6-Difluoro-3-nitrophenyl)-1-((2,6-difluorophenyl)amino)propan-2-ol (**14j**)

2,6-Difluoro-aniline (39 mg, 0.30 mmol) and 2-(2,6-difluoro-3-nitrobenzyl)oxirane (65 mg, 0.30 mmol) were reacted according to the general procedure for 19 h to yield aminol **14j** as an off-white solid (26 mg, 25%, mp. 74–76 °C) along with 26% recovery of epoxide. *R*_f_ = 0.23 in DCM; IR (neat): ν_max_ 3346, 3256, 3100, 2933, 1621 cm^–1^; ^1^H NMR (400.13 MHz, CD_3_CN): δ 8.04 (ddd, *J* = 9.2 Hz, 8.6 Hz, 5.7 Hz, 1H), 7.12 (ddd, *J* = 9.2 Hz, 8.6 Hz, 1.8 Hz, 1H), 6.91–6.81 (m, 2H), 6.72–6.66 (m, 1H), 4.31 (bs, NH), 3.98–3.90 (m, 1H), 3.50–3.44 (m, 1H), 3.31–3.20 (m, 2H, overlapping OH), 2.95–2.84 (m, 2H); ^13^C{^1^H} NMR (100.61 MHz, CD_3_CN): δ 165.5 (dd, *J* = 256 Hz, 8 Hz), 156.2 (dd, *J* = 263 Hz, 10 Hz), 154.3 (dd, *J* = 239 Hz, 8 Hz), 135.4, 126.9 (t, *J* = 14 Hz), 126.6 (dd, *J* = 12 Hz, 1 Hz), 118.7 (dd, *J* = 21 Hz, 19 Hz), 118.5 (t, *J* = 10 Hz), 112.7–112.5 (m), 112.5 (dd, *J* = 16 Hz, 7 Hz), 70.3, 52.3 (t, *J* = 4 Hz), 29.1; ^19^F NMR (376.46 MHz, CD_3_CN): δ −103.2 (d, *J* = 14.0 Hz, 1F), −117.7 (d, *J* = 14.0 Hz, 1F), −130.1 (s, 2F); HRMS (ESI/TOF): calcd for C_15_H_13_F_4_N_2_O_3_^+^ [M + H]^+^ 345.08513, found 345.08577.

#### Synthesis of 3-(4-Chlorophenyl)-1-(2,6-difluoro-4-nitrophenyl)aminopropan-2-ol (**14k**)

2,6-Difluoro-4-nitroaniline (272 mg, 1.56 mmol) and 2-(4-chlorobenzyl)oxirane (220 mg, 1.30 mmol) were reacted according to the general procedure for 24 h, and isolation by silica-gel flash column chromatography (pet. ether/DCM 4:6) yielded the target compound **14 k** (31 mg, 7%, *R*_f_ 0.17, DCM).

#### Synthesis of 1-Azido-3-(4-chlorophenyl)propan-2-ol

To a stirred solution of 2-(4-chlorobenzyl)oxirane (**13**) (214 mg, 1.27 mmol) in MeOH (2.7 mL) and water (0.3 mL), were added NaN_3_ (248 mg, 3.81 mmol) and NH_4_Cl (136 mg, 2.54 mmol) at rt. The reaction mixture was stirred at rt. for 18 h. MeOH was removed under reduced pressure and water (5 mL) and EtOAc (5 mL) were added. The phases were separated, and the aq. layer was extracted with EtOAc (3 × 10 mL). The combined organic layers were dried (MgSO_4_), filtered, and concentrated under reduced pressure to give the title compound as a colorless oily liquid (250 mg, 93%), which was essentially pure based on ^1^H NMR. *R*_f_ = 0.59 in pet. ether/EtOAc 1:1; IR (neat): ν_max_ 3415, 2922, 2096, 1491 cm^–1^; ^1^H NMR (400.13 MHz, CDCl_3_): δ 7.30 (d, *J* = 8.5 Hz, 2H), 7.15 (d, *J* = 8.6 Hz, 2H), 4.00–3.94 (m, 1H), 3.39 (dd, *J* = 12.5 Hz, 3.7 Hz, 1H), 3.29 (dd, *J* = 12.5 Hz, 6.8 Hz, 1H), 2.79–2.77 (m, 2H); ^13^C{^1^H} NMR (100.61 MHz, CDCl_3_): δ 135.7, 132.9, 130.8, 129.0, 71.6, 56.1, 40.2; HRMS (EI/TOF): 156, 154, 127, 125 (100), 91, 89, 65, 63 Calcd for C_8_H_7_^35^ClO^+•^ [M-C_2_H_4_N_3_]^+•^ 154.01799, found 154.01802.

#### Synthesis of 1-Amino-3-(4-chlorophenyl)propan-2-ol Hydrochloride

1-Azido-3-(4-chlorophenyl)propan-2-ol (520 mg, 2.46 mmol) and PPh_3_ (708 mg, 2.70 mmol) in a mixture of THF (9 mL) and water (1 mL) were stirred at 50 °C under Ar for 2 h. THF was removed under reduced pressure, and EtOAc (20 mL) and 6 M aq. hydrochloric acid (20 mL) were added. The phases were separated, and the organic layer was extracted with water (2 × 10 mL). The combined aq. phases were then washed with Et_2_O (40 mL) and concentrated under reduced pressure. Traces of water were azeotropically removed by adding toluene (5 mL) followed by evaporation under reduced pressure. Three repetitions of this process gave the title compound as sharp white needles (459 mg, 84%, mp. 188–190 °C). *R*_f_ = 0.17 as freebase in DCM/EtOH/NH_3_ (20%) 89:10:1; IR (neat): ν_max_ 3452, 3209, 2913, 1600 cm^–1^; ^1^H NMR (400.13 MHz, D_2_O) δ 7.30 (d, *J* = 8.4 Hz, 2H), 7.21 (d, *J* = 8.4 Hz, 2H), 4.10–4.04 (m, 1H), 3.17 (dd, *J* = 13.1 Hz, 2.5 Hz, 1H), 2.93 (dd, *J* = 13.0 Hz, 10.2 Hz, 1H), 2.84 (dd, *J* = 14.0 Hz, 4.9 Hz, 1H), 2.72 (dd, *J* = 14.0 Hz, 8.4 Hz, 1H); ^13^C{^1^H} NMR (100.61 MHz, D_2_O) δ 135.8, 131.9, 130.9, 128.5, 68.6, 44.1, 39.7; HRMS (ESI/TOF): calcd for C_9_H_13_NO^35^Cl^+^ [M + H]^+^ 186.06747, found 186.06890.

#### Synthesis of 3-(4-Chlorophenyl)-1-(2,6-difluoro-4-nitrophenyl)aminopropan-2-ol (**14k**)

A solution of 1-amino-3-(4-chlorophenyl)propan-2-ol hydrochloride (222 mg, 1.00 mmol), 3,4,5-trifluoronitrobenzene (128 μL, 194 mg, 1.10 mmol), and DIPEA (700 μL, 519 mg, 4.00 mmol) in ACN (6 mL) was stirred at 40 °C under Ar for 14 h. The product was isolated by silica-gel column chromatography (DCM), and concentration of the relevant fractions (*R*_f_ = 0.18 in DCM) yielded **14k** as a yellow crystalline solid (273 mg, 80%, mp. 130–132 °C). IR (neat): ν_max_ 3491, 3294, 3095, 3023, 2897, 1610 cm^–1^; ^1^H NMR (400.13 MHz, CDCl_3_): δ 7.78 (dd, *J* = 8.1 Hz, 2.1 Hz, 2H), 7.31 (d, *J* = 8.4 Hz, 2H), 7.16 (d, *J* 8.4 Hz, 2H), 4.80 (bs, NH), 4.07–4.01 (m, 1H), 3.75 (d, *J* = 13.4 Hz, 1H), 3.43 (dd, *J* = 13.4 Hz, 8.2 Hz, 1H), 2.87 (dd, *J* = 13.7 Hz, 4.5 Hz, 1H), 2.74 (dd, *J* = 13.7 Hz, 8.5 Hz, 1H); ^13^C{^1^H} NMR (100.61 MHz, CDCl_3_): δ 150.1 (dd, *J* = 243.6 Hz, 9.0 Hz), 135.9 (t, *J* = 10.7 Hz), 135.5, 133.1, 132.7 (t, *J* = 12.6 Hz), 130.8, 129.1, 109.0 (dd, *J* = 18.2 Hz, 9.6 Hz), 71.8, 50.1 (t, *J* = 4.5 Hz), 40.9; ^19^F NMR (376.46 MHz, CDCl_3_): δ −128.8; HRMS (ESI/TOF): Calcd for C_15_H_12_N_2_O_3_^35^ClF_2_^–^ [M – H]^−^ 341.05155, found 341.05100.

#### General Procedure for Photodecomposition of **14a**–**14d**

A solution of the appropriate compound (≈0.10 mmol) in ACN was added to a photochemical reactor containing distilled water at the appropriate pH to a concentration of ≈0.7 mM and a total volume of either 75 or 150 mL (CAN/water 7:3), depending on the reactor size. The reaction vessel was either purged with N_2_ during the reaction or left open to air. The reaction mixture was photolyzed with a 6 W low-pressure mercury-vapor lamp (mainly 254 nm irradiation). After completion, the reaction mixture was transferred to a separatory funnel, saturated with NaCl, and extracted with EtOAc (3 × 50 mL). The pH was adjusted to ≈2 and the aqueous layer was extracted again with EtOAc (3 × 50 mL). The combined organic layers were dried (MgSO_4_), filtered, and concentrated in vacuo on a rotary evaporator to yield a residue which was analyzed by ^1^H NMR.

##### Photolysis of 1-((2,5-Dichloro-4-(1,1,2,3,3,3-hexa-fluoropropoxy)phenyl)amino)-3-(4-nitrophenyl)propan-2-ol (**14a**)

A solution of compound **14a** (26.6 mg, 0.052 mmol) was photolyzed in a 75 mL (0.70 mM) photochemical reactor according to the general procedure with a 6 W medium-pressure mercury-vapor lamp for 24 h at pH 13 and 8. The resulting reaction mixture was worked up according to the general procedure.

##### Photolysis of 1-((3,5-Dichloro-2-fluorophenyl)amino)-3-(2,6-difluoro-3-nitrophenyl)propan-2-ol (**14b**)

A solution of compound **14b** (20.4 mg, 0.052 mmol) was photolyzed in a 75 mL (0.69 mM) photochemical reactor according to the general procedure with a 6 W medium-pressure mercury-vapor lamp for 24 h at pH 13 and 8. The aqueous layer was extracted with EtOAc. The resulting reaction mixture was worked up according to the general procedure.

##### Photolysis of 1-((3,5-Dichloro-2,4-difluorophenyl)-amino)-3-(2,6-difluoro-3-nitrophenyl)propan-2-ol (**14c**)

A solution of compound **14c** (21.0 mg, 0.051 mmol) was photolyzed in a 75 mL (0.68 mM) photochemical reactor according to the general procedure with a 6 W medium-pressure mercury-vapor lamp for 24 h at pH 13 and 8. The aqueous layer was extracted with EtOAc. The resulting reaction mixture was worked up according to the general procedure.

##### Photolysis of 1-((2,5-Dichloro-4-(1,1,2,3,3,3-hexa-fluoropropoxy)phenyl)amino)-3-(2,6-difluoro-3-nitrophenyl)propan-2-ol (**14d**)

A solution of compound **14d** (26.8 mg, 0.049 mmol) was photolyzed in a 75 mL (0.66 mM) photochemical reactor according to the general procedure with a 6 W medium-pressure mercury-vapor lamp for 24 h at pH 13 and 8. The aqueous layer was extracted with EtOAc. The resulting reaction mixture was worked up according to the general procedure.

## Supporting Information Available

Table S1, Figures S1–S4, and NMR spectra of all new compounds; integration data from the photolysis of compound **5a** in an NMR tube; ^1^H-NMR spectra of compound **9a** before and after photolysis; ^1^H-NMR spectra of compound **14a** before and after photolysis; ^1^H NMR spectra of compound **14b** before and after photolysis; and ^19^F NMR of compounds **14b** and **14c** before and after photolysis (PDF)
