# Supplementary material for: Photodegradable Antimicrobial Agents: Synthesis and Mechanism of Degradation
Source: J Org Chem. 2022 Jun 2;87(12):8034–47. doi: 10.1021/acs.joc.2c00681 (PMC9207920; doi:10.1021/acs.joc.2c00681)

Supporting information

Photodegradable Antimicrobial Agents – Synthesis and Mechanism of Degradation

Vebjørn Eikemo,[a] Bjarte Holmelid,[b] Leiv K. Sydnes[b] & Magne O. Sydnes[a]*

[a]Department of Chemistry, Bioscience and Environmental Engineering, Faculty of Science and Technology, University of Stavanger, NO-4036 Stavanger, Norway
[b]Department of Chemistry, University of Bergen, NO-5007 Bergen, Norway

magne.o.sydnes@uis.no

Table of Contents

Appendix 1 .... S2

NMR spectra of all compounds S7

Appendix 1

Table S1. Results from the photodecomposition of compound **5a** in an NMR tube.[a]

Reaction time (min.)	Integral of signal at 6.53 ppm of the compound **5a**
0.00	1.35
5.00	1.27
10.00	1.17
15.00	1.15
25.00	1.05
35.00	0.94
45.00	0.85
60.00	0.76
75.00	0.69
90.00	0.64
110.00	0.60
130.00	0.55
150.00	0.53
180.00	0.52
210.00	0.51
240.00	0.42
1440.00	0.38

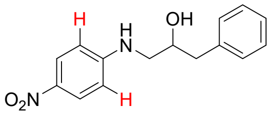

[a]The sample was dissolved in acetonitrile-d_3/water-d_2 (7/3 v/v). The residual solvent peak of acetonitrile-d_3 was used as internal reference and the integral for that signal was set to one. The integral for the two ortho protons of the aniline moiety (marked red in the structure) of compound **5a** was compared to the integral of acetonitrile to establish the decomposition rate.

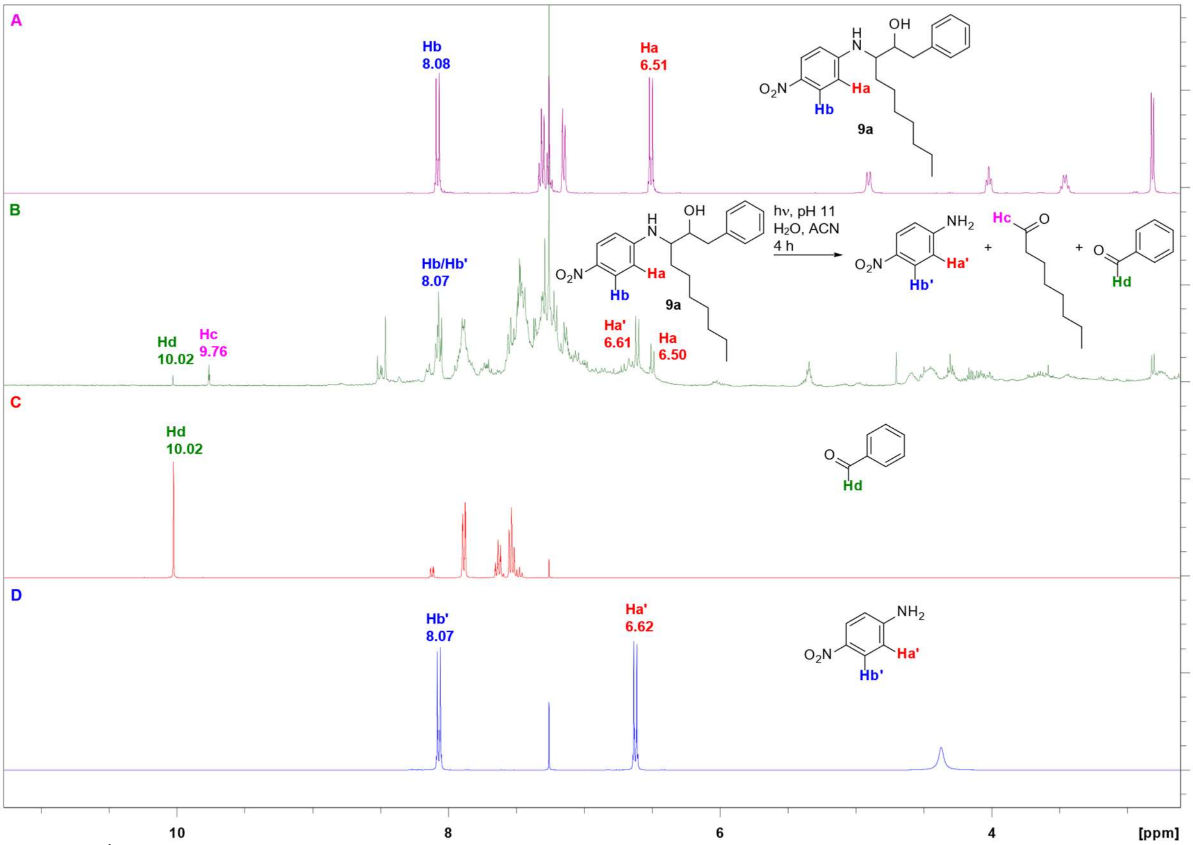

Figure S1. ^{1}H NMR spectra (A) Pure compound **9a**; (B) Crude degradation mixture after 4 h photolysis at pH 11; (C) Decomposition product benzaldehyde; (D) Decomposition product *p*-nitroaniline.

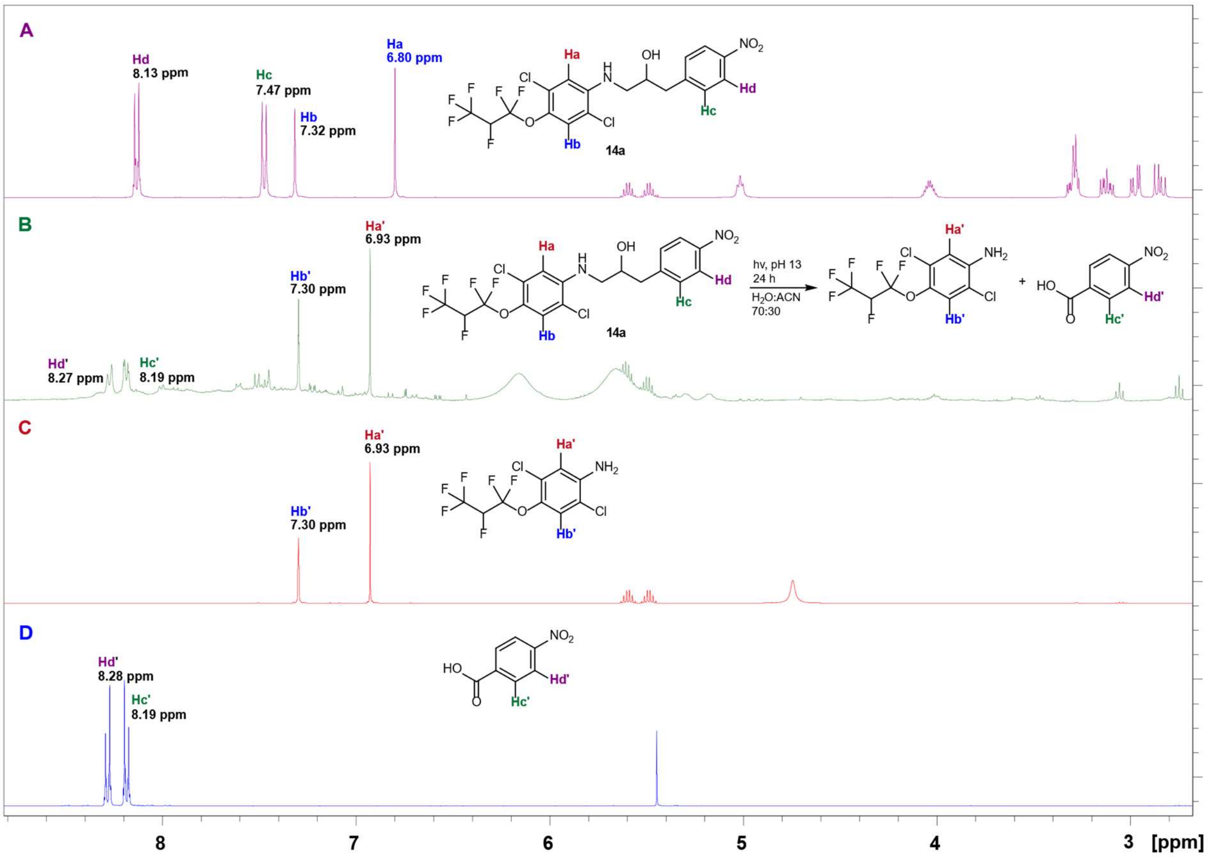

Figure S2. ^{1}H NMR spectra (A) Pure compound **14a**; (B) Crude degradation mixture after 24 h photolysis at pH 13; (C) Decomposition product 2,5-dichloro-4-(1,1,2,3,3-hexafluoropropoxy)aniline; (D) Decomposition product *p*-nitrobenzoic acid.

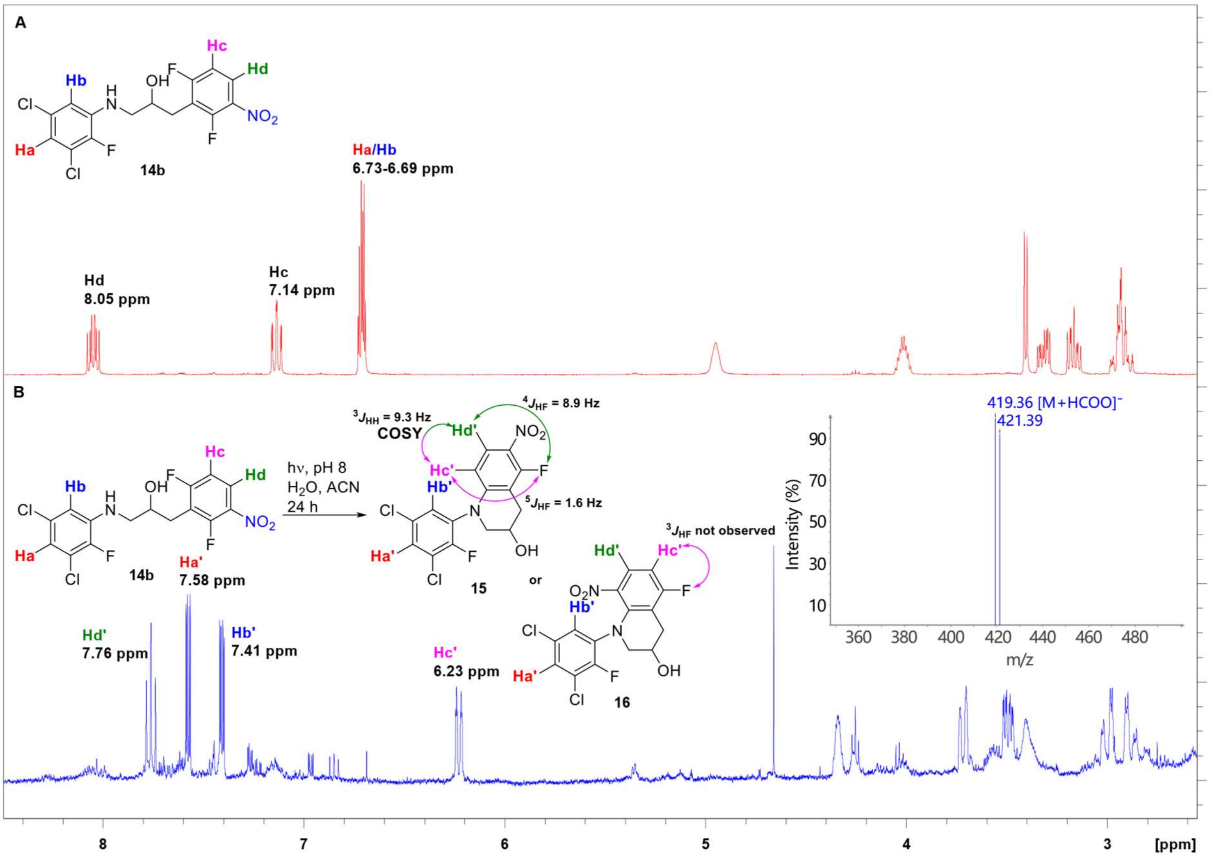

Figure S3. (A) ^{1}H-NMR spectrum of compound **14b** after 24 h in the dark at pH 8 showing no signs of instability; (B) ^{1}H-NMR spectrum of crude degradation mixture after 24 h photolysis of **14b** at pH 8 with the low-resolution MS inserted in the top right corner.

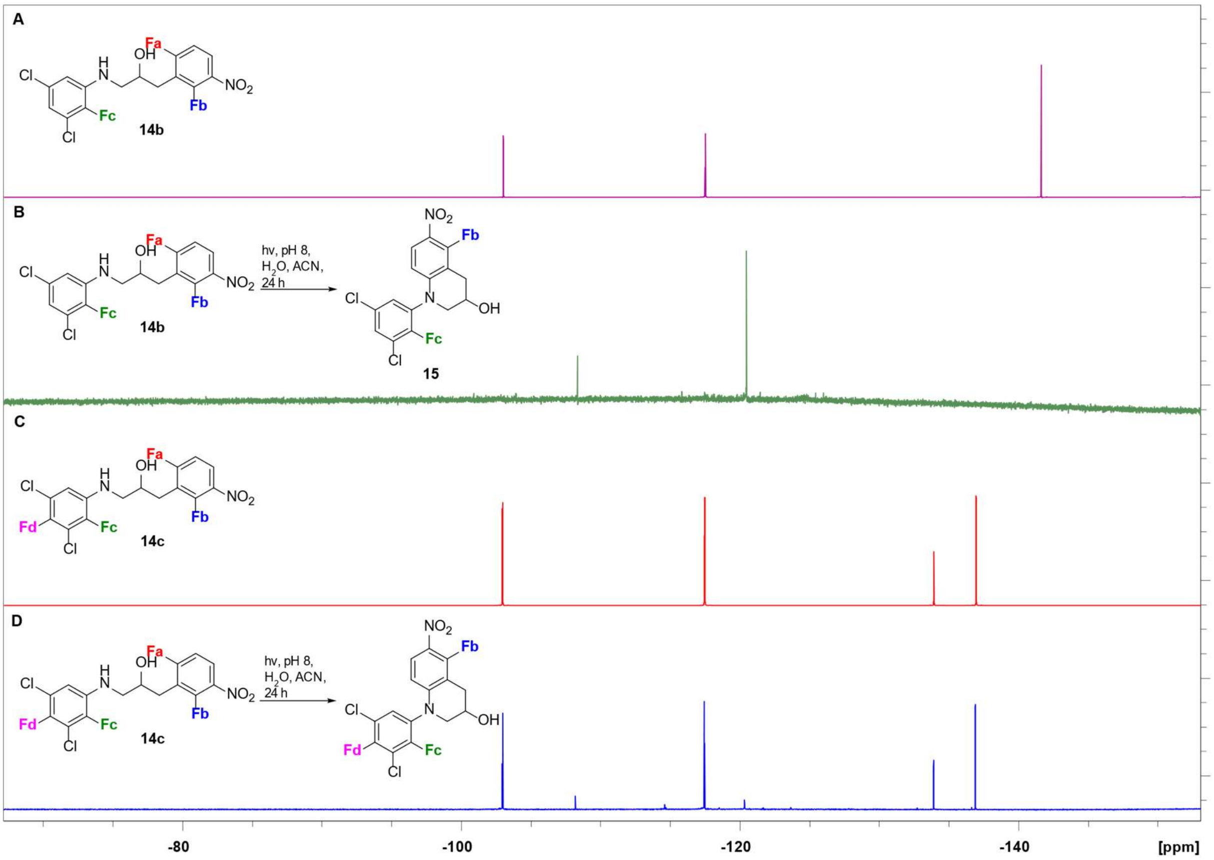

Figure S4. ^{19}F-NMR spectra of compound **14b** and **14c** before and after photolysis displaying the loss of one fluorine atom in the photoreaction.

NMR spectra of all compounds

^{1}H NMR spectrum of **5a** (400.13 MHz, $CDCl_3$)

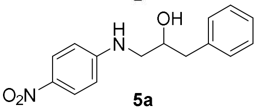

5a

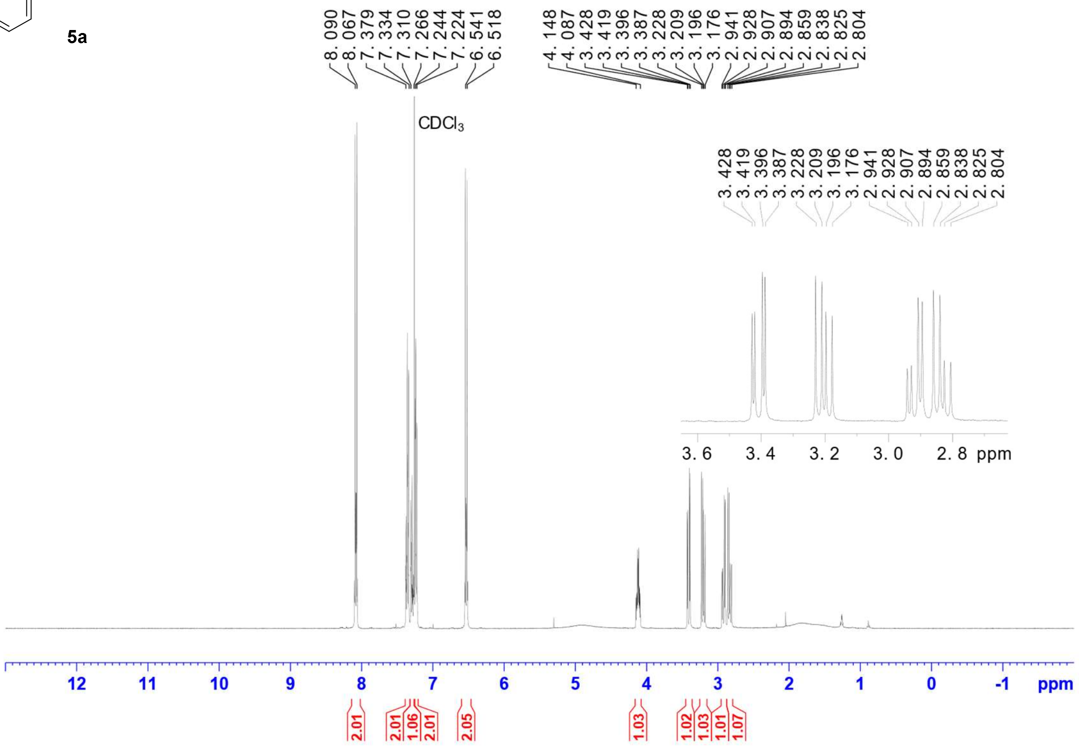

$^{13}C\{^{1}H\}$ NMR spectrum of **5a** (100.61 MHz, $CDCl_3$)

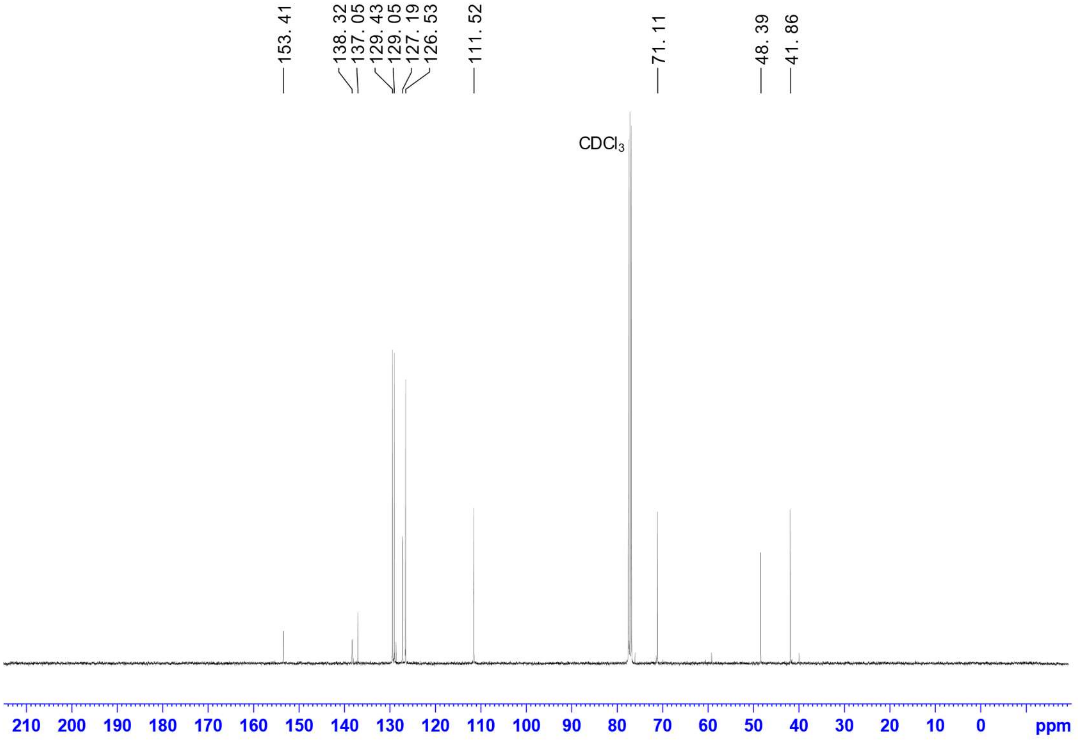

^{1}H NMR spectrum of **5b** (400.13 MHz, $CDCl_3$)

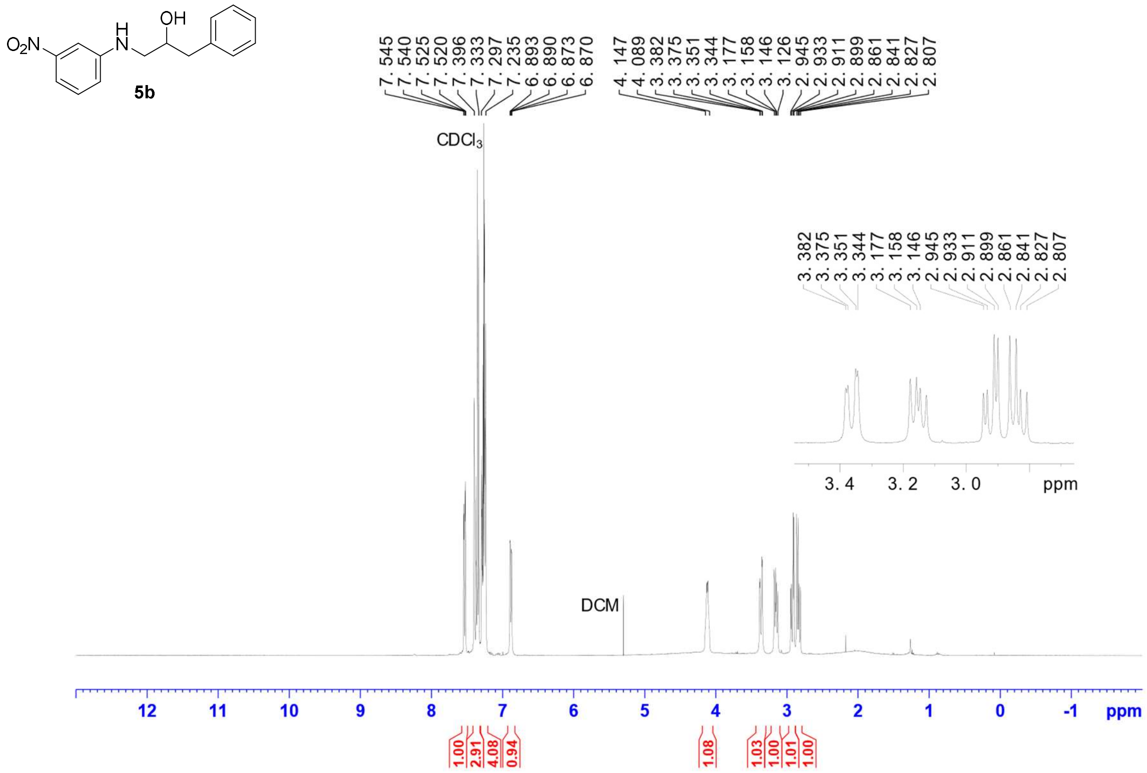

^{13}C{^{1}H} NMR spectrum of **5b** (100.61 MHz, $CDCl_3$)

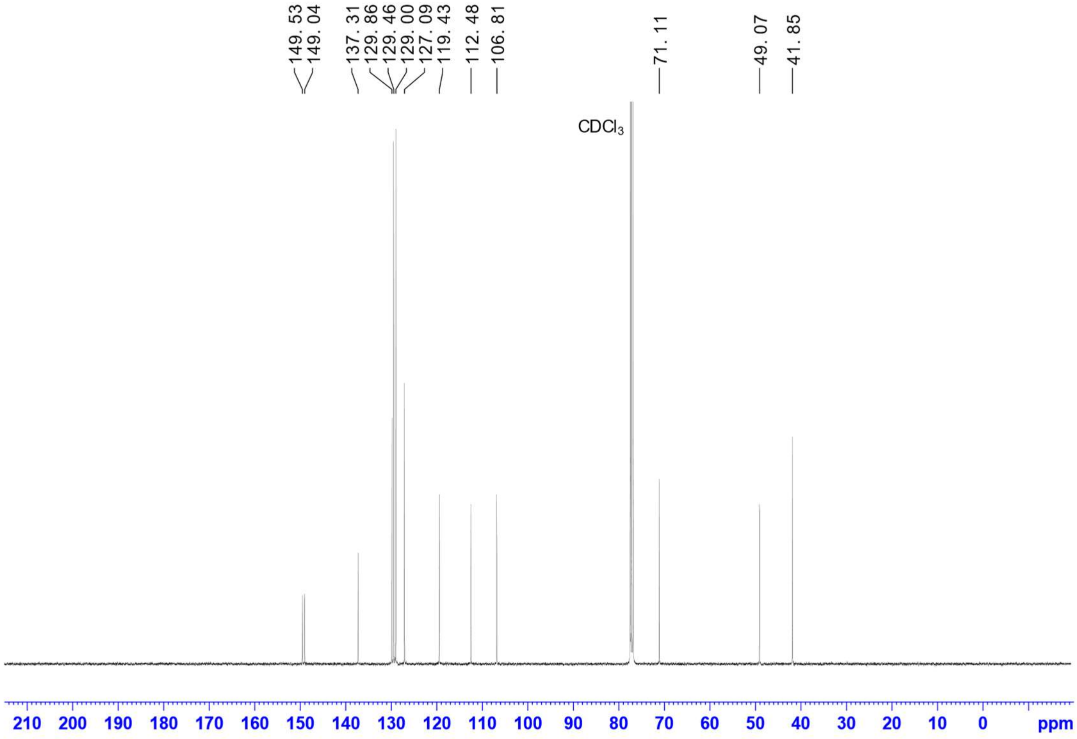

^{1}H NMR spectrum of 1-allyl-4-nitrobenzene (400.13 MHz, $CDCl_3$)

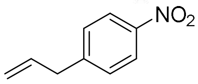

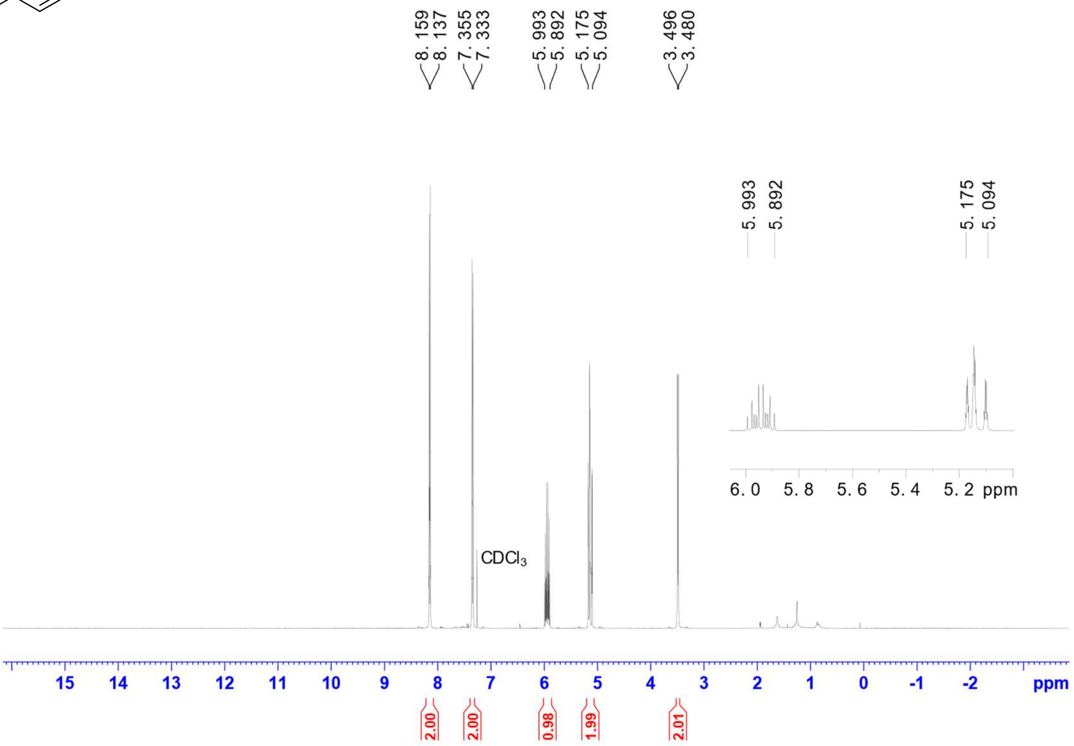

^{13}C{^{1}H} NMR spectrum of 1-allyl-4-nitrobenzene (100.61 MHz, $CDCl_3$)

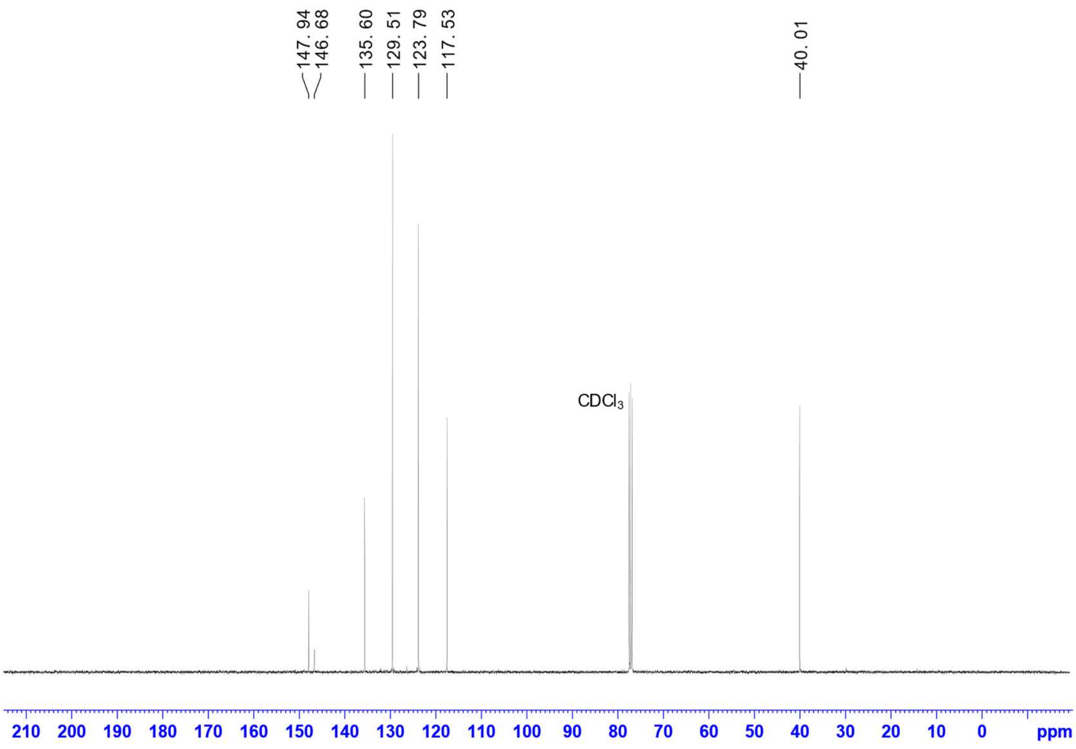

^{1}H NMR spectrum of 2-(4-nitrobenzyl)oxirane (400.13 MHz, $CDCl_3$)

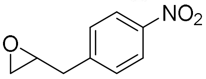

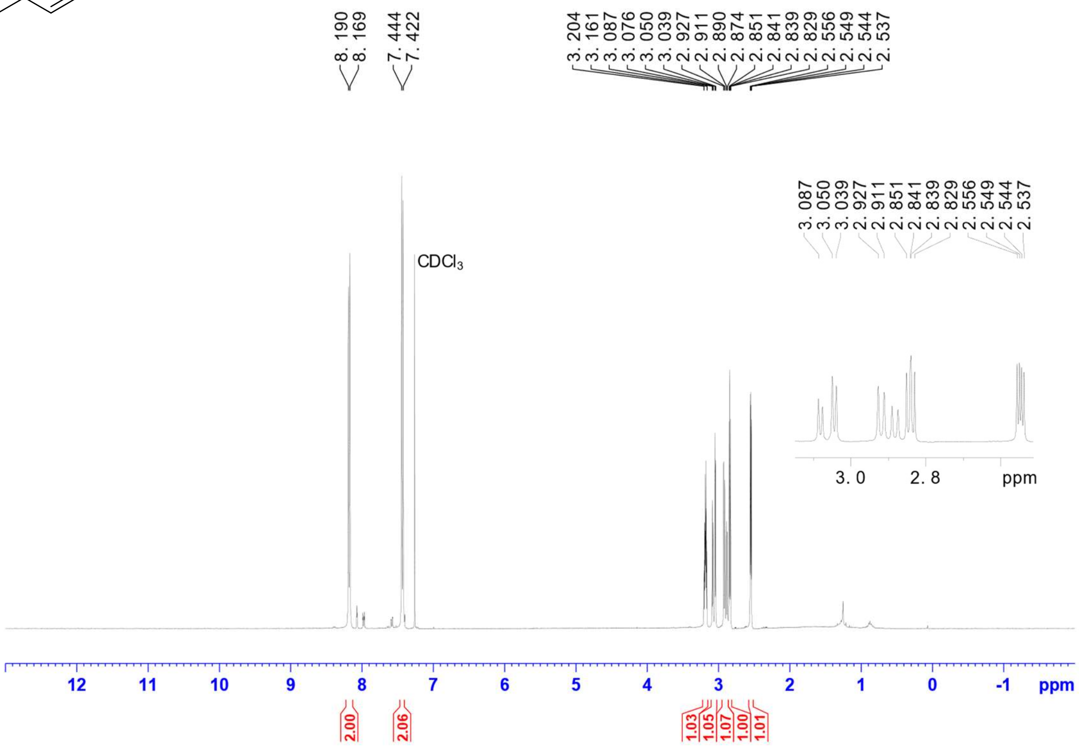

$^{13}C\{^{1}H\}$ NMR spectrum of 2-(4-nitrobenzyl)oxirane (100.61 MHz, $CDCl_3$)

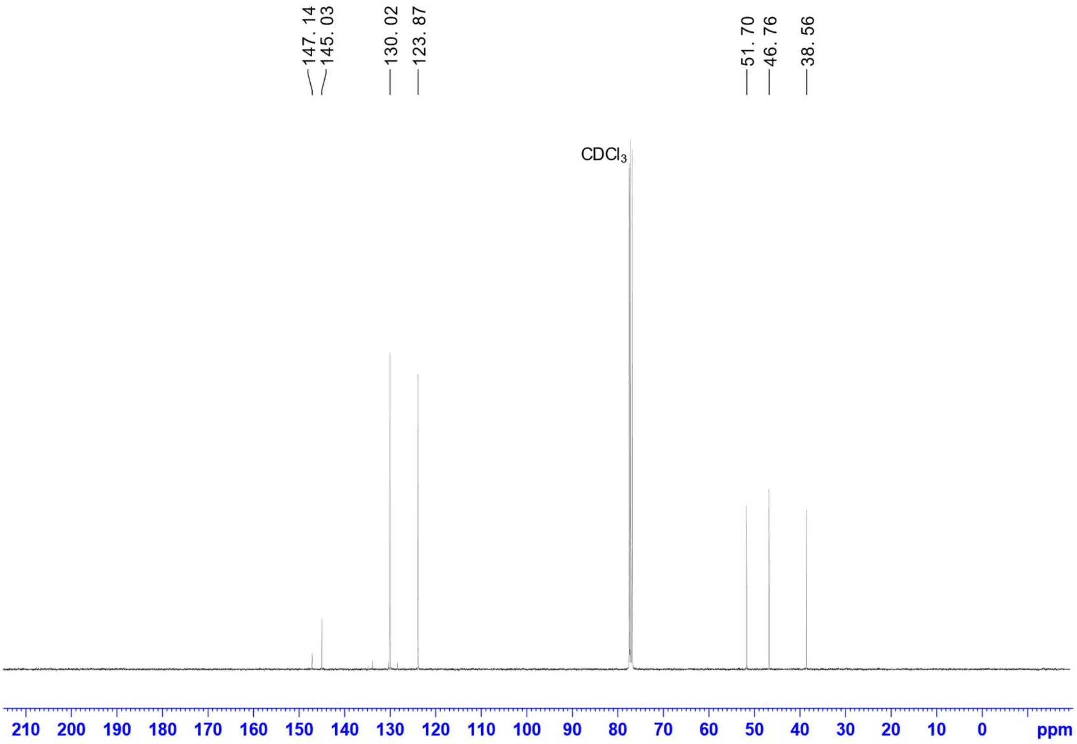

^{1}H NMR spectrum of **5c** (400.13 MHz, $CDCl_3$)

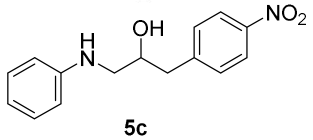

5c

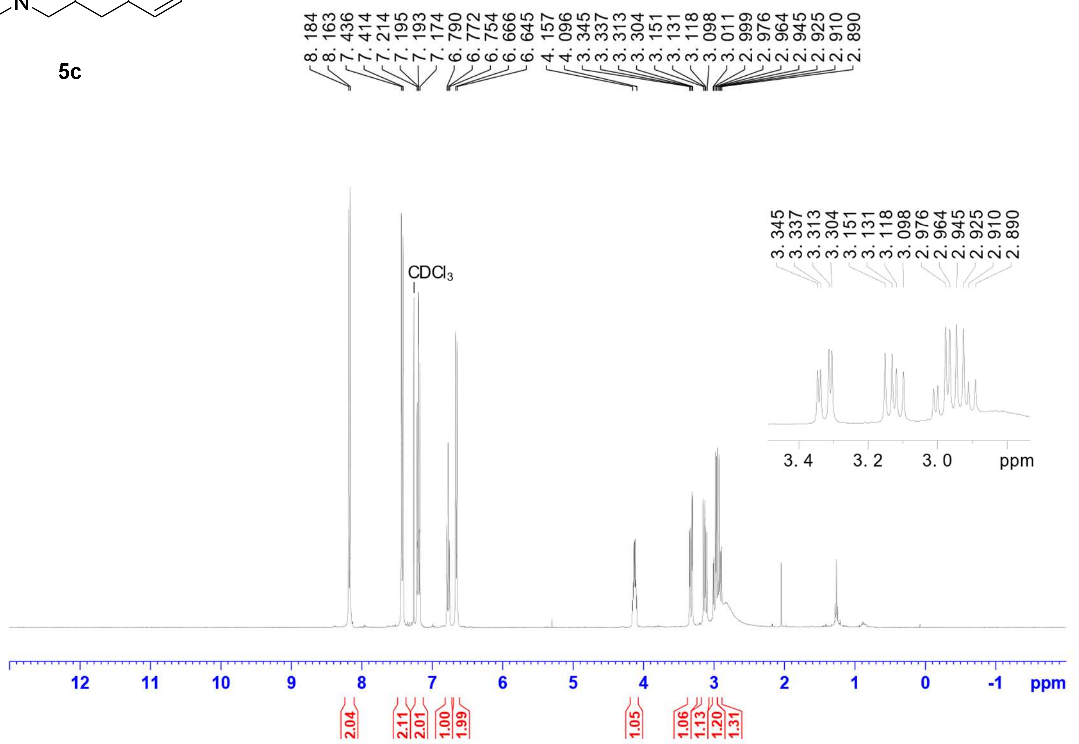

$^{13}C\{^{1}H\}$ NMR spectrum of **5c** (100.61 MHz, $CDCl_3$)

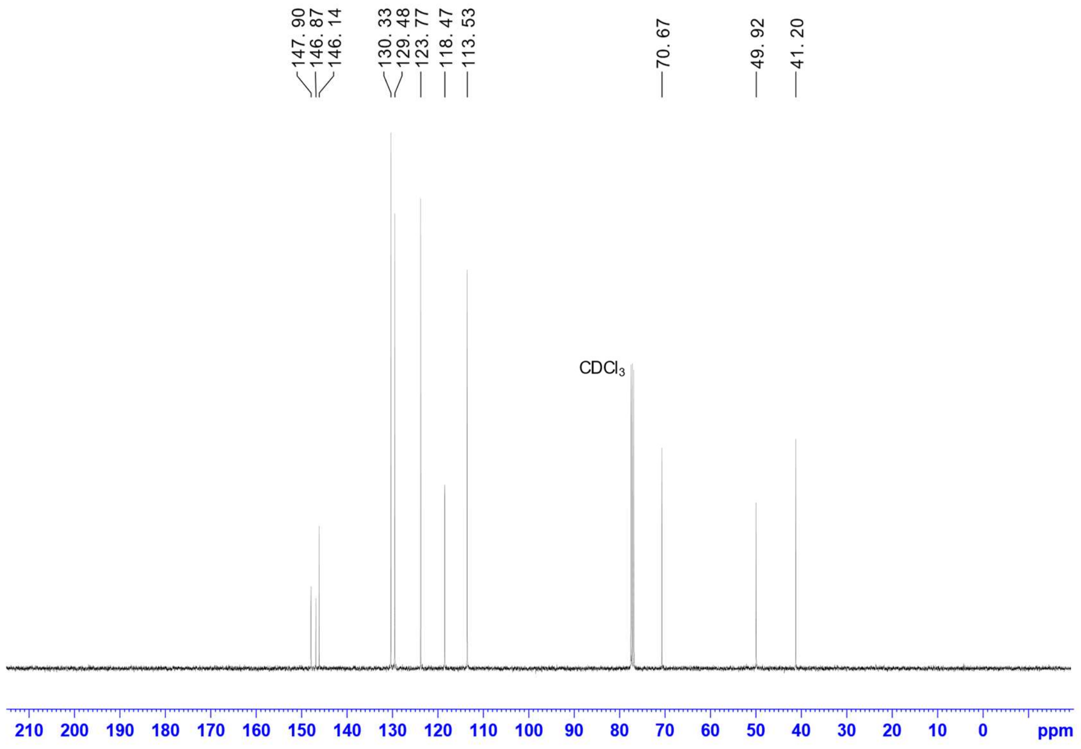

^{1}H NMR spectrum of 1-allyl-3-nitrobenzene (400.13 MHz, $CDCl_3$)

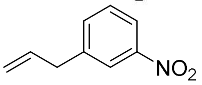

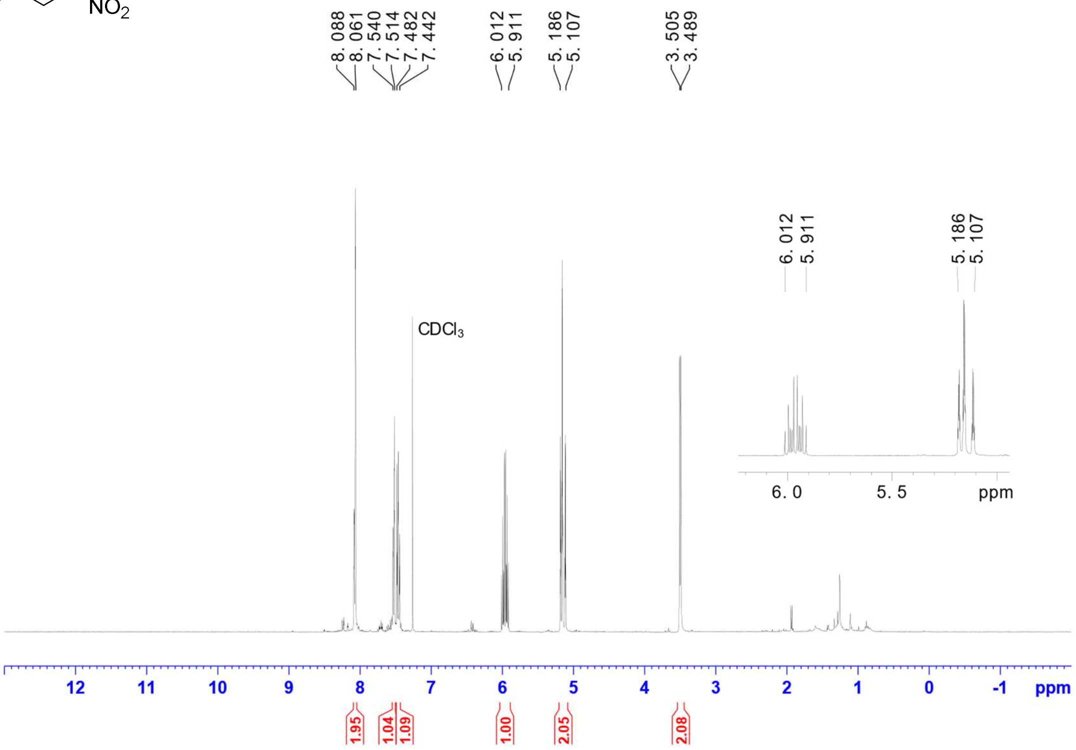

^{13}C{^{1}H} NMR spectrum of 1-allyl-3-nitrobenzene (100.61 MHz, $CDCl_3$)

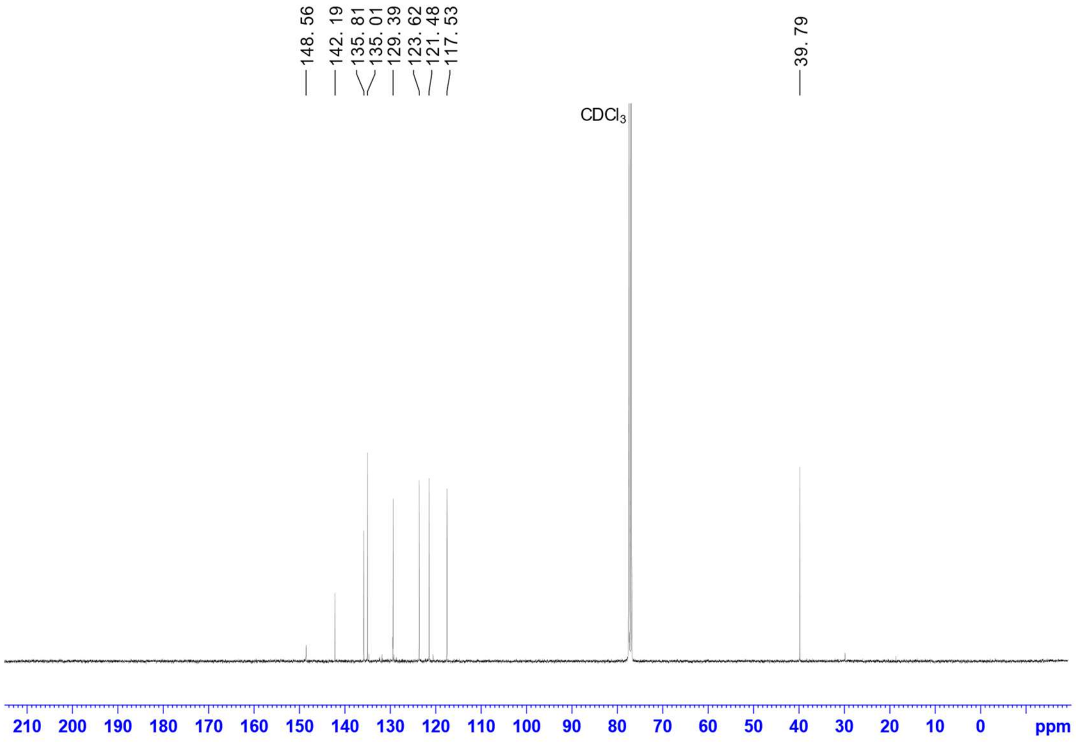

^{1}H NMR spectrum of 2-(3-nitrobenzyl)oxirane (400.13 MHz, $CDCl_3$)

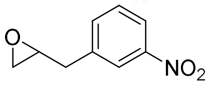

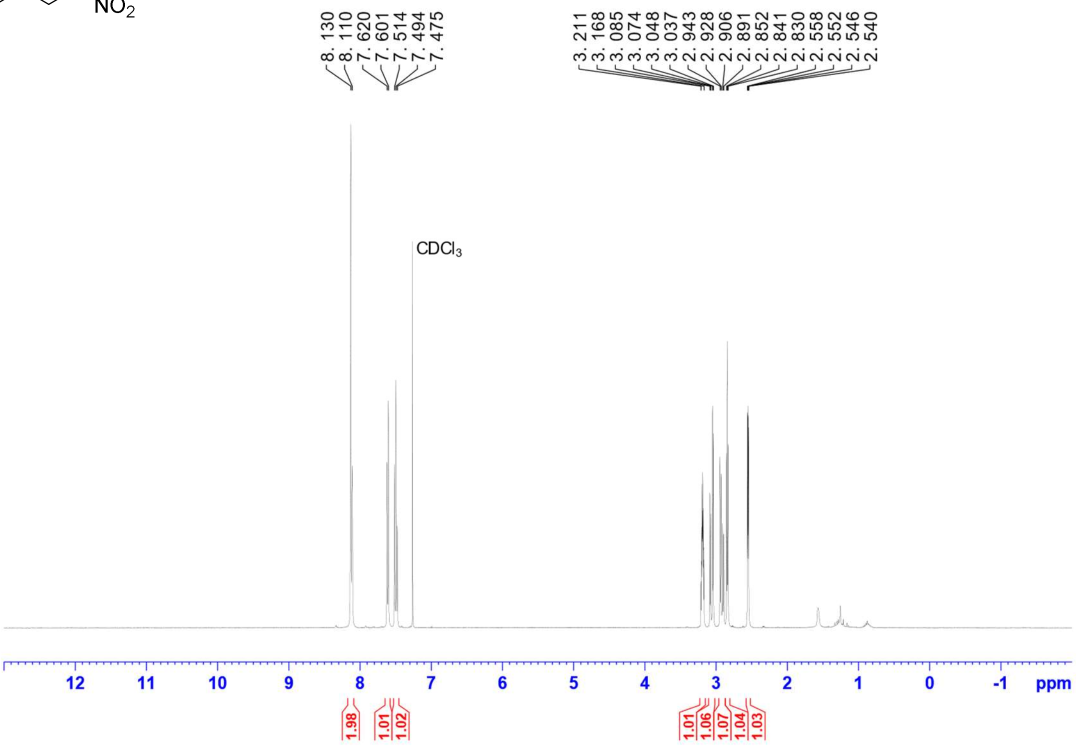

^{13}C{^{1}H} NMR spectrum of 2-(3-nitrobenzyl)oxirane (100.61 MHz, $CDCl_3$)

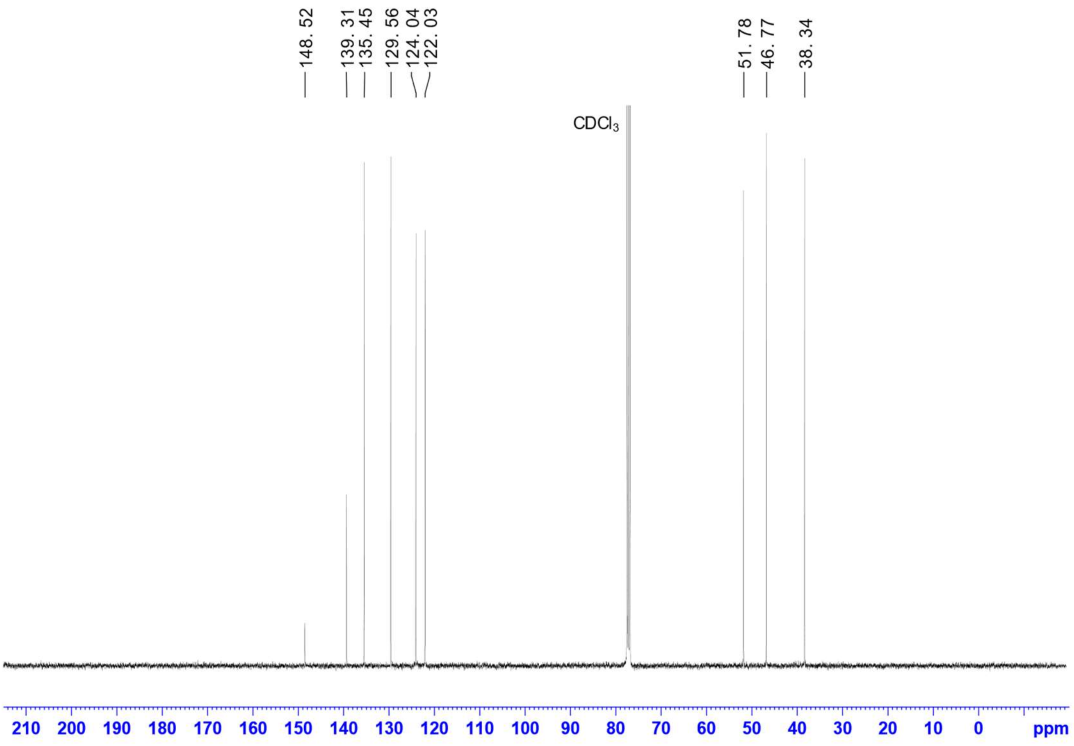

^{1}H NMR spectrum of **5d** (400.13 MHz, $CDCl_3$)

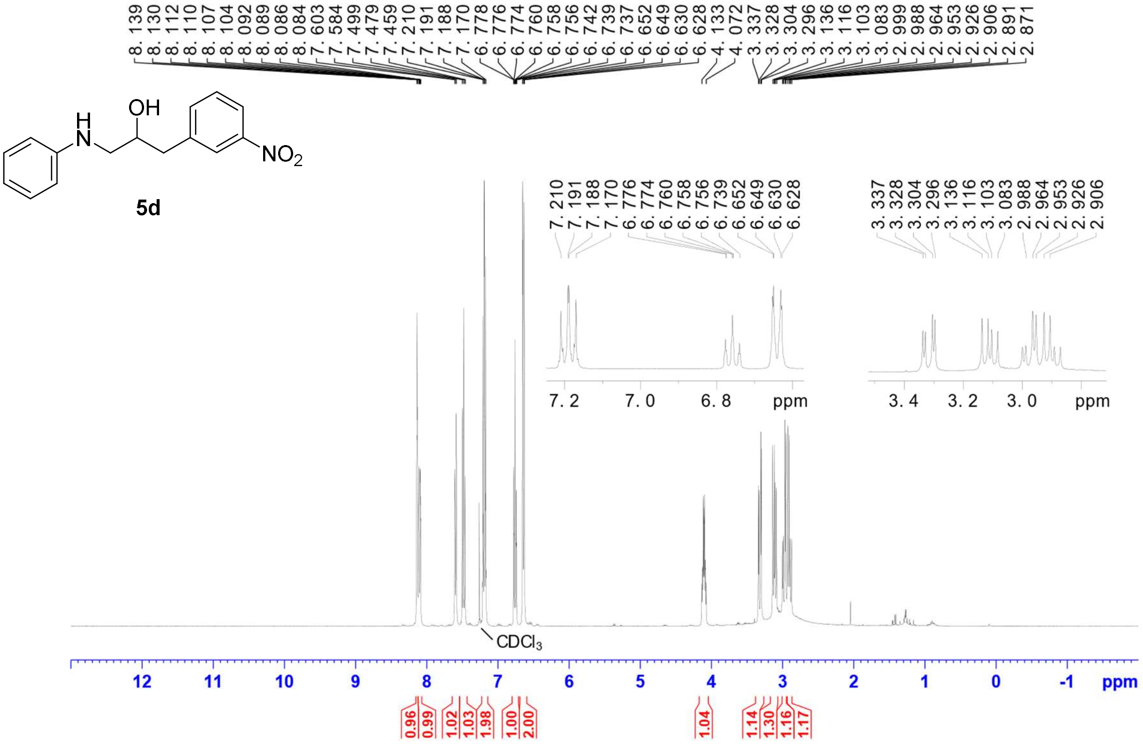

$^{13}C\{^{1}H\}$ NMR spectrum of **5d** (100.61 MHz, $CDCl_3$)

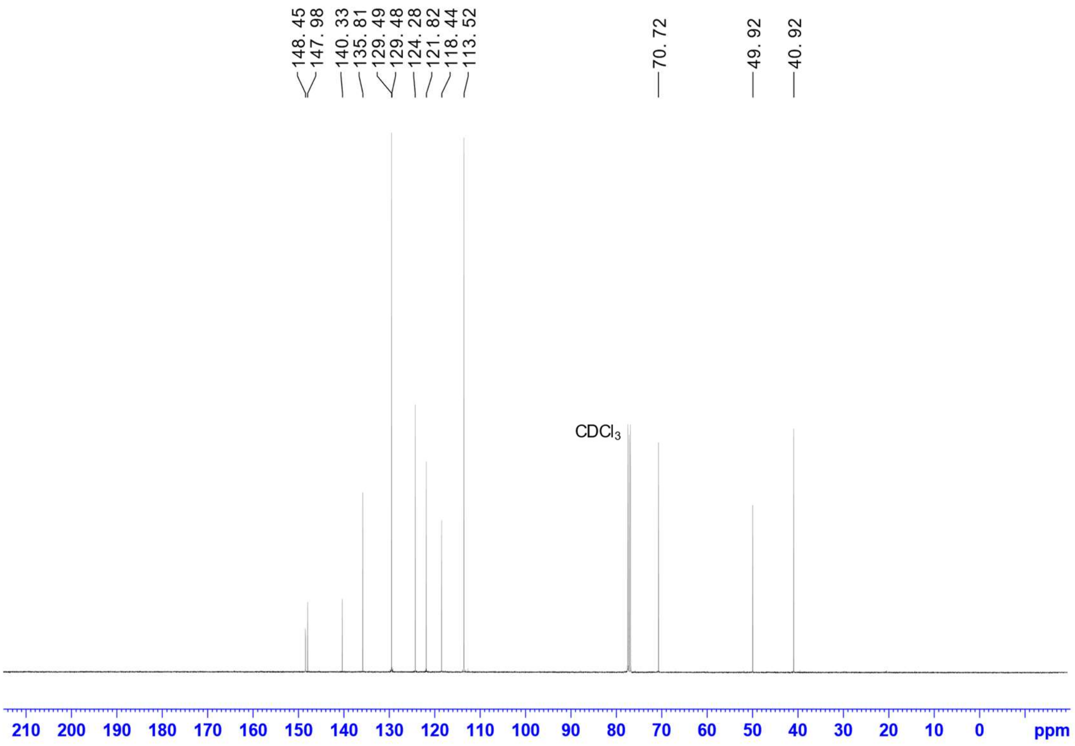

^{1}H NMR spectrum of octyltriphenylphosphonium bromide (400.13 MHz, $CDCl_3$)

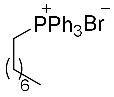

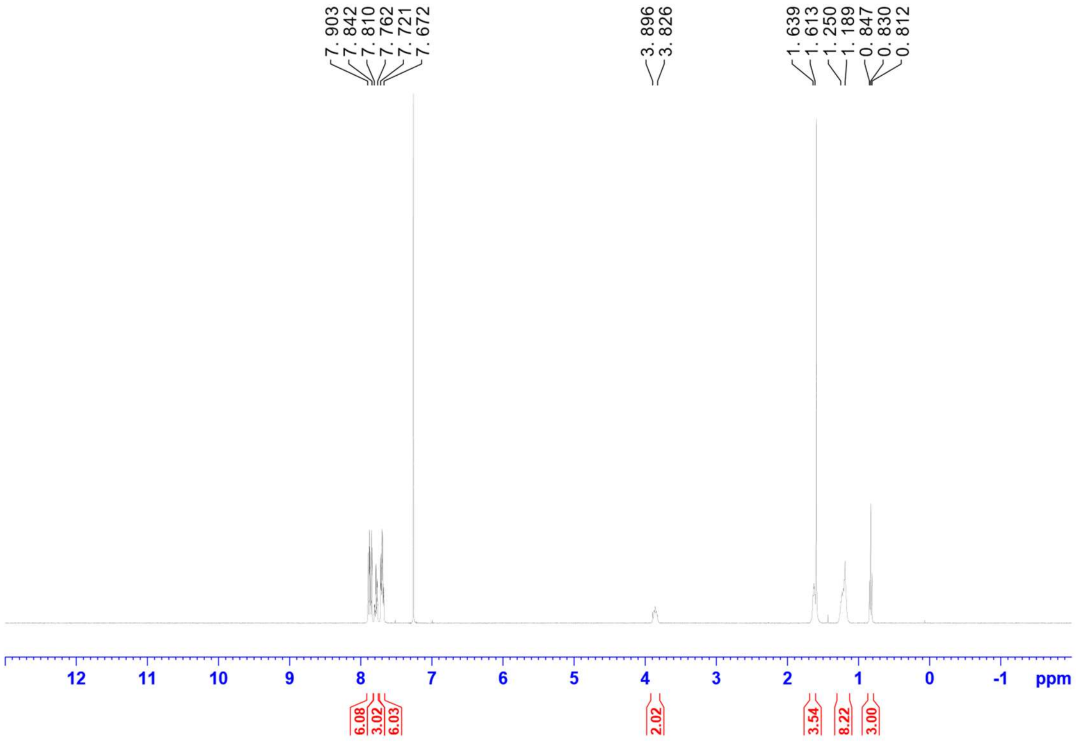

^{13}C{^{1}H} NMR spectrum of octyltriphenylphosphonium bromide (100.61 MHz, $CDCl_3$)

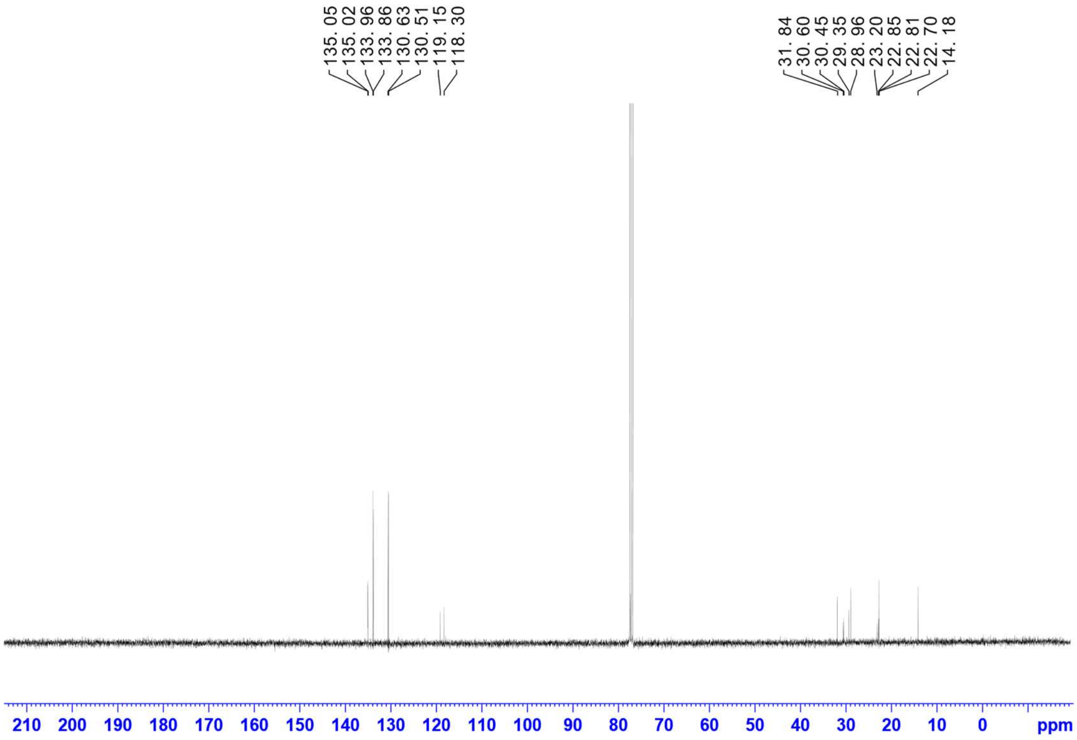

^{31}P NMR spectrum of octyltriphenylphosphonium bromide (161.98 MHz, $CDCl_3$)

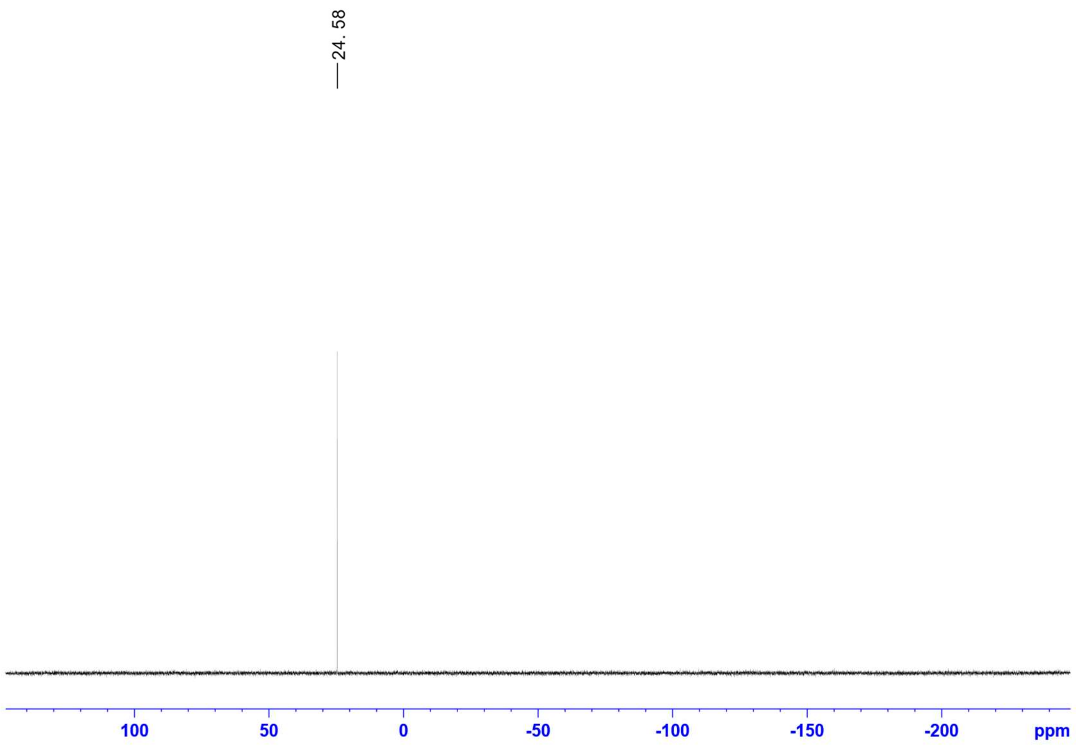

^{1}H NMR spectrum of 1-phenyldec-2-ene (400.13 MHz, $CDCl_3$)

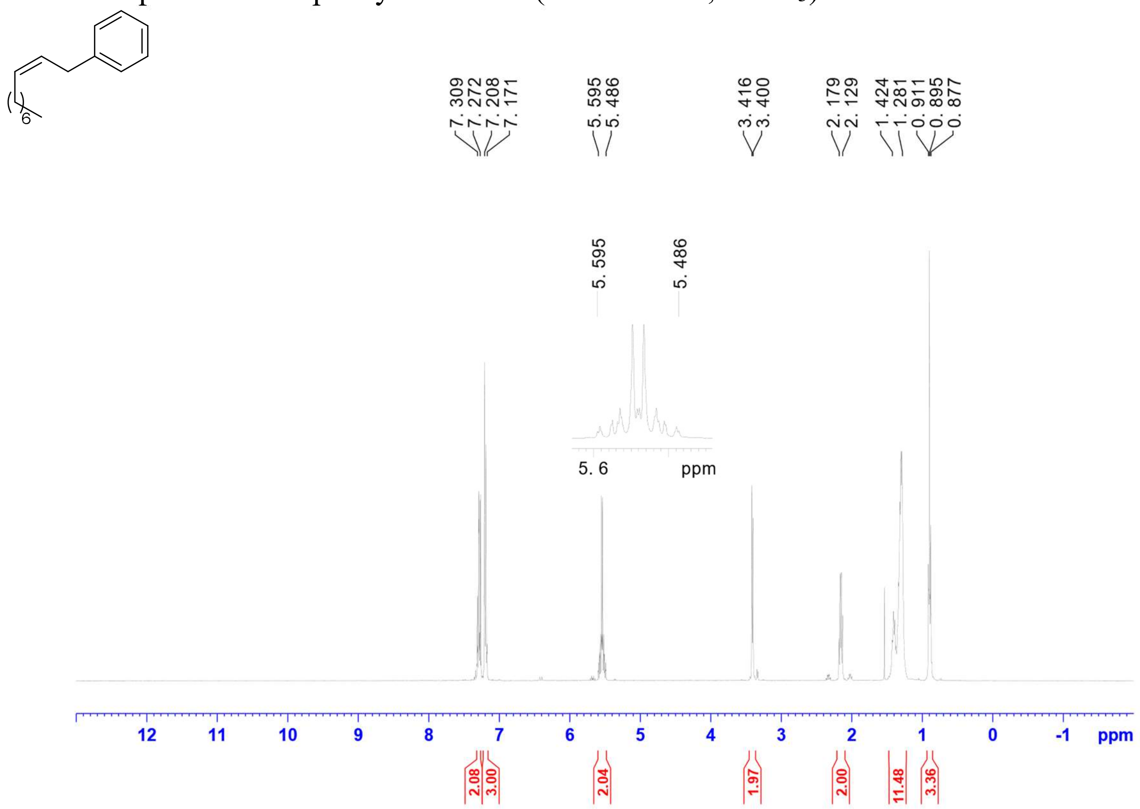

$^{13}C\{^{1}H\}$ NMR spectrum of 1-phenyldec-2-ene (100.61 MHz, $CDCl_3$)

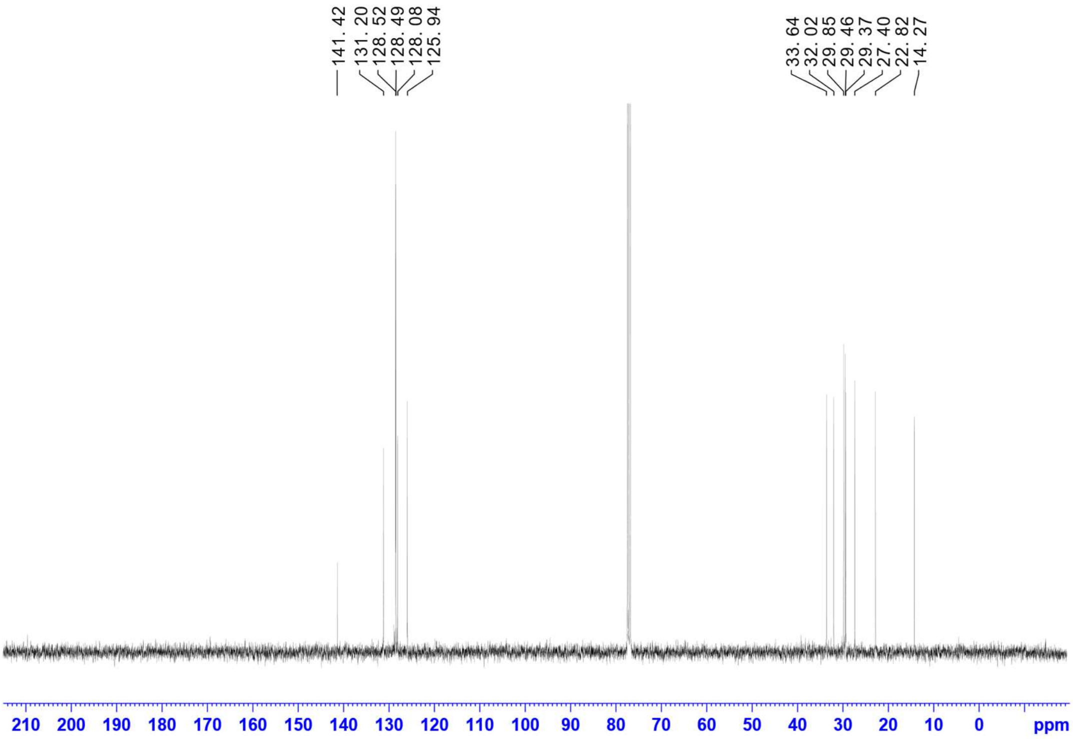

^{1}H NMR spectrum of *cis*-2-benzyl-3-heptyloxirane (400.13 MHz, $CDCl_3$)

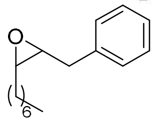

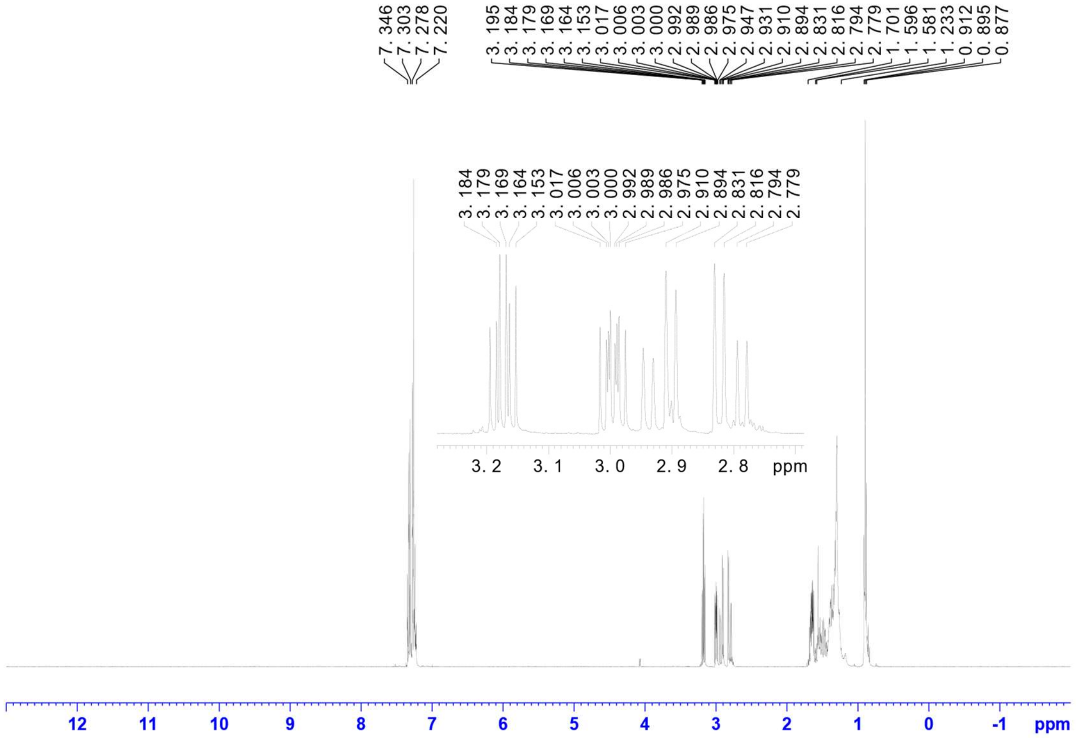

^{13}C{^{1}H} NMR spectrum of *cis*-2-benzyl-3-heptyloxirane (100.61 MHz, $CDCl_3$)

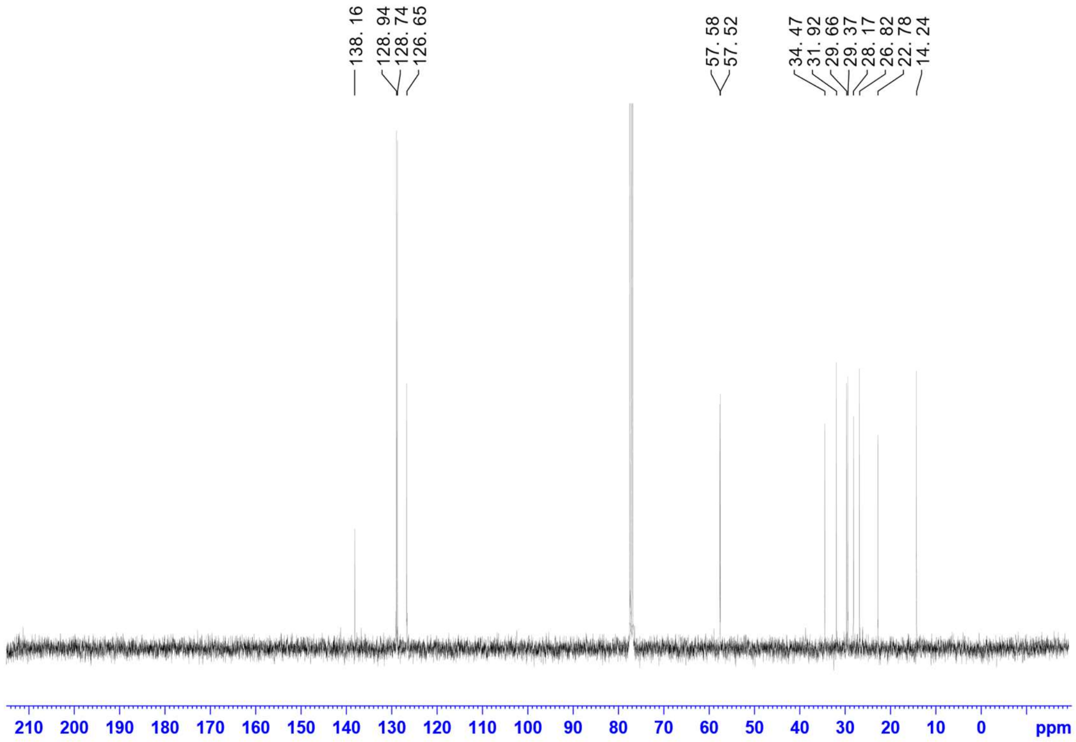

^{1}H NMR spectrum of **7a** and **7b** (400.13 MHz, $CDCl_3$)

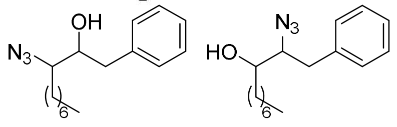

7a **7b**

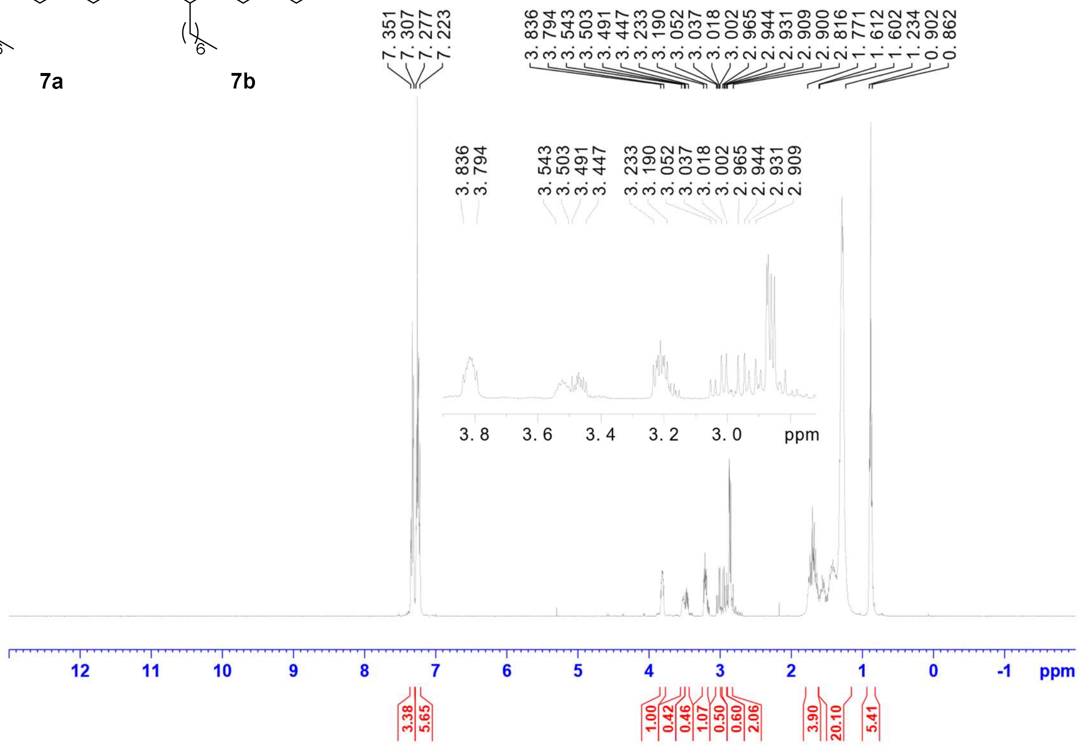

$^{13}C\{^1H\}$ NMR spectrum of **7a** and **7b** (100.61 MHz, $CDCl_3$)

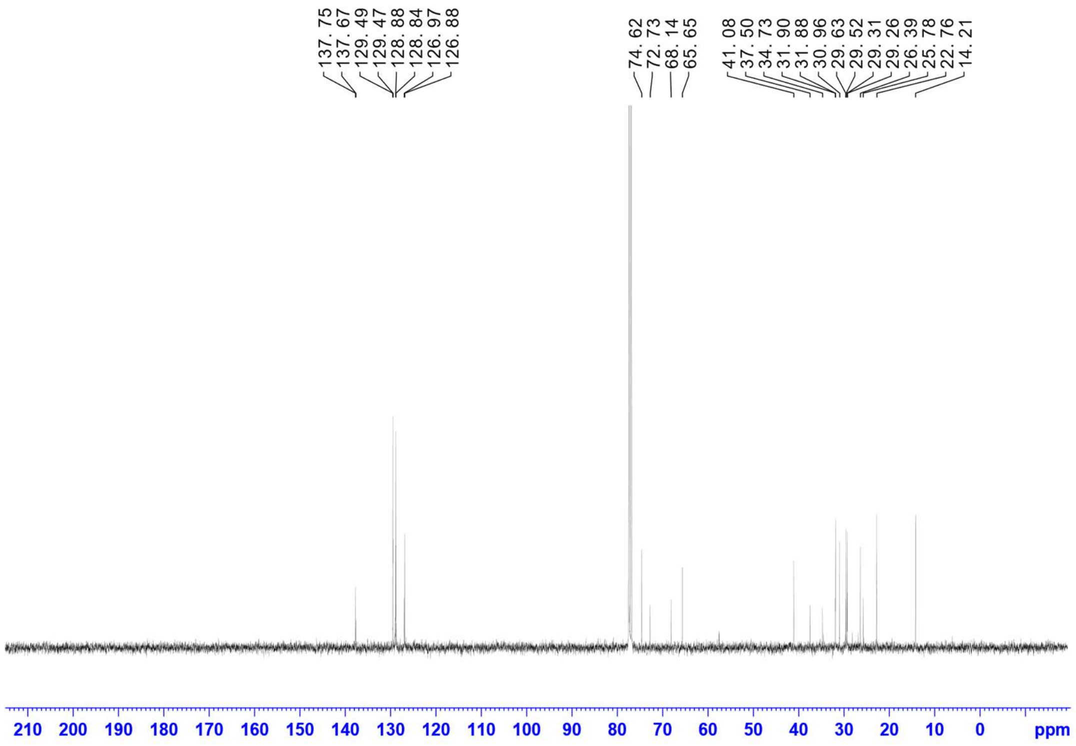

^{1}H NMR spectrum of **8a** and **8b** (400.13 MHz, $CDCl_3$)

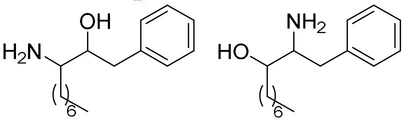

8a **8b**

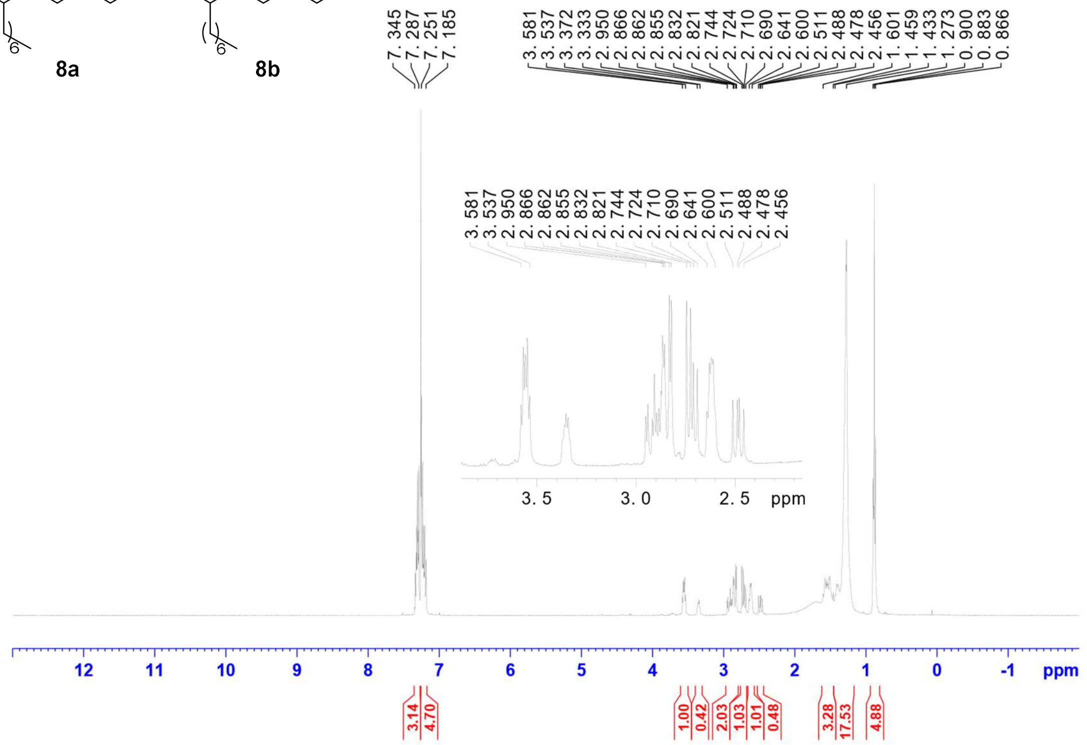

^{13}C{^{1}H} NMR spectrum of **8a** and **8b** (100.61 MHz, $CDCl_3$)

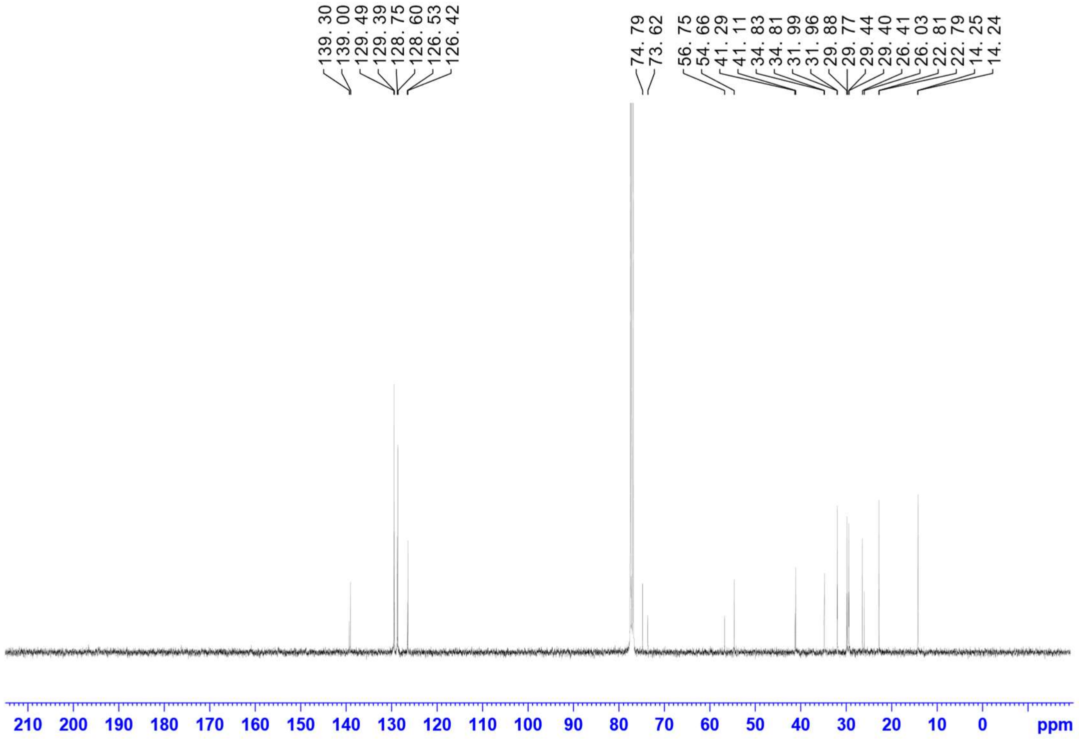

^{1}H NMR spectrum of **9a** (400.13 MHz, $CDCl_3$)

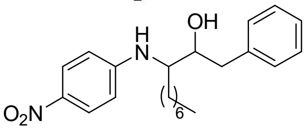

9a

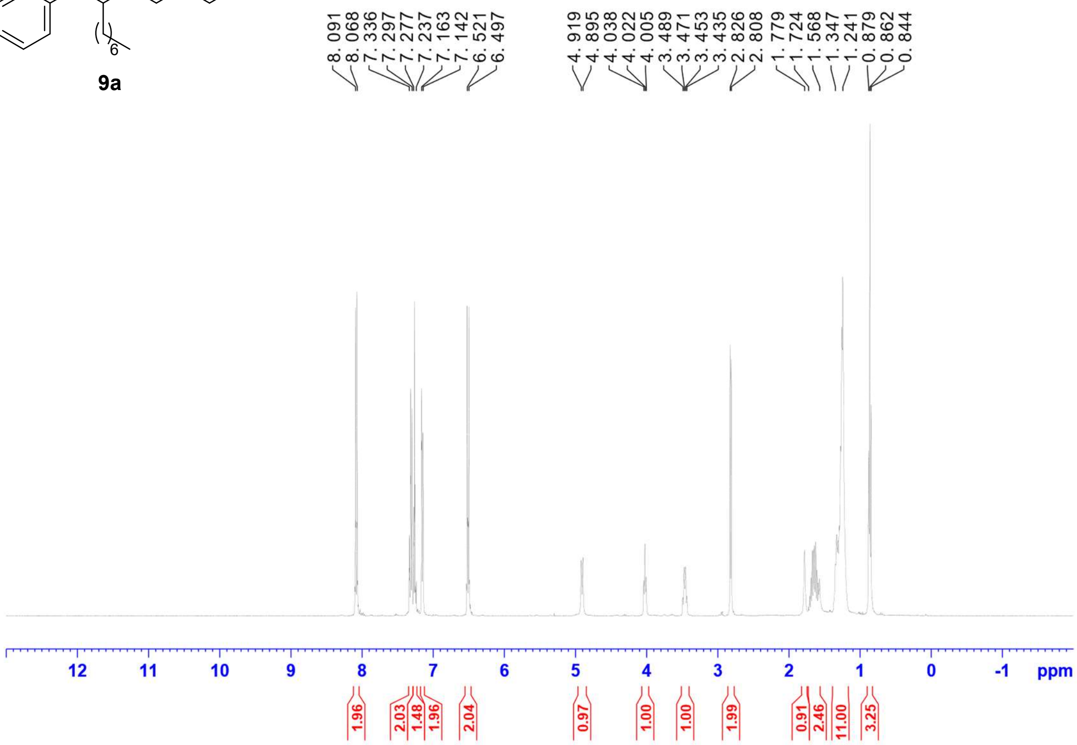

^{13}C{^{1}H} NMR spectrum of **9a** (100.61 MHz, $CDCl_3$)

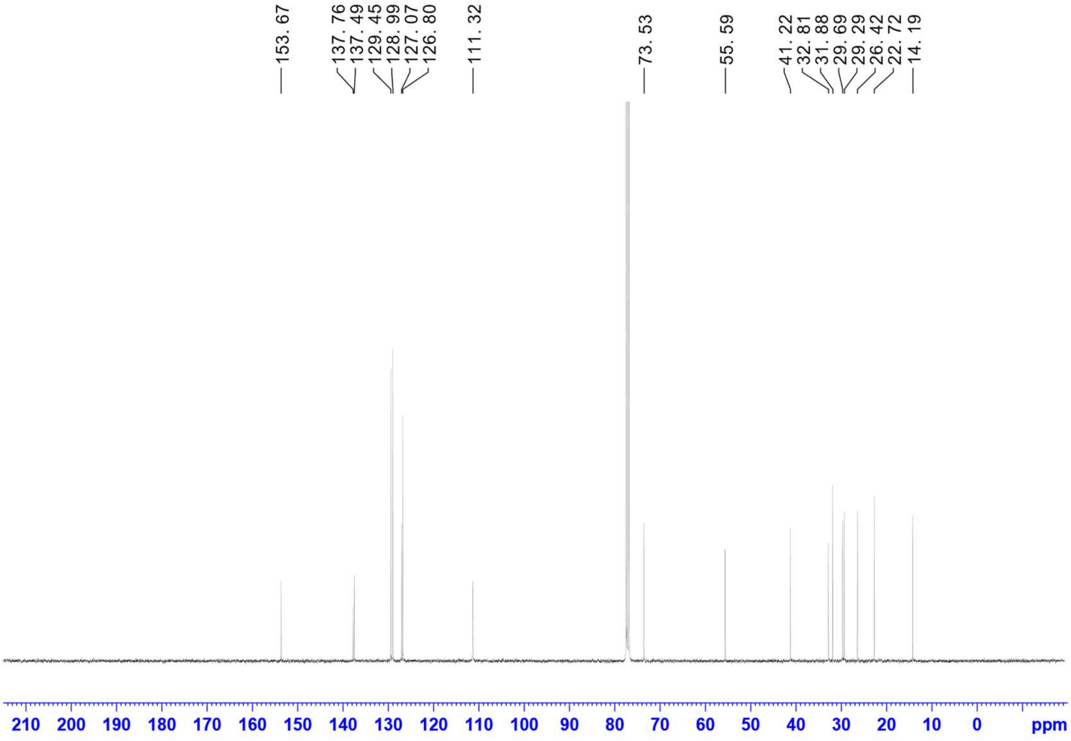

^{1}H NMR spectrum of *N*-(3,5-dichloro-2-fluorophenyl)acetamide (400.13 MHz, $CDCl_3$)

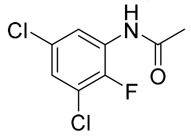

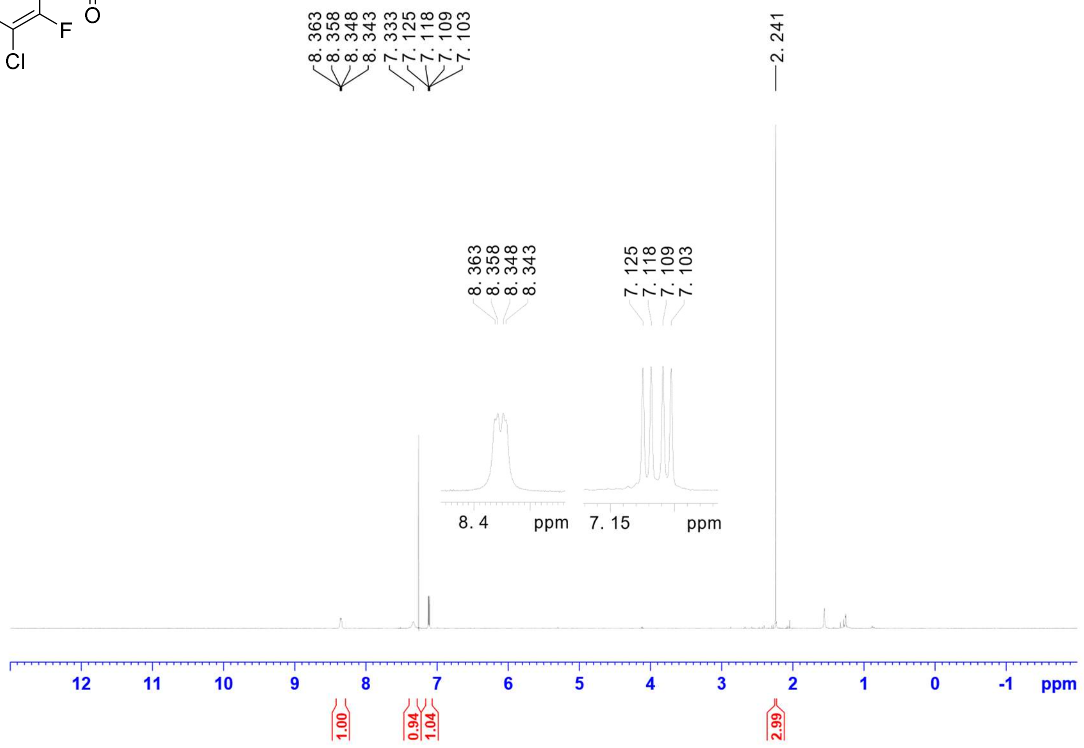

^{13}C{^{1}H} NMR spectrum of *N*-(3,5-dichloro-2-fluorophenyl)acetamide (100.61 MHz, $CDCl_3$)

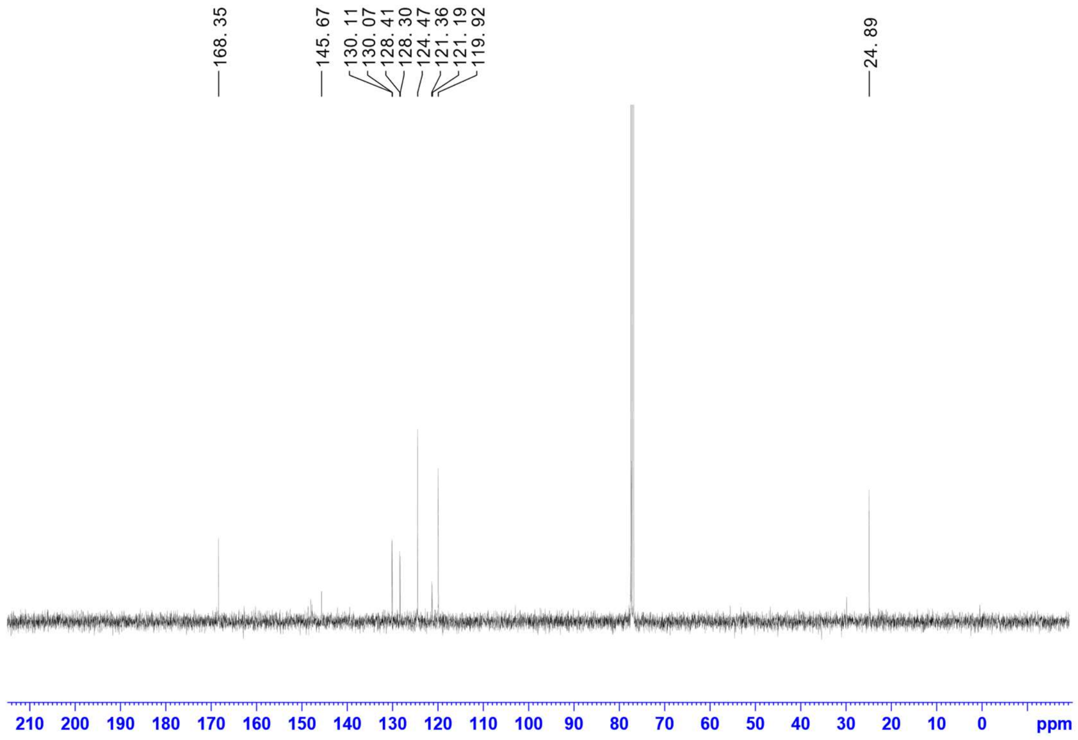

^{19}F NMR spectrum of *N*-(3,5-dichloro-2-fluorophenyl)acetamide (376.46 MHz, $CDCl_3$)

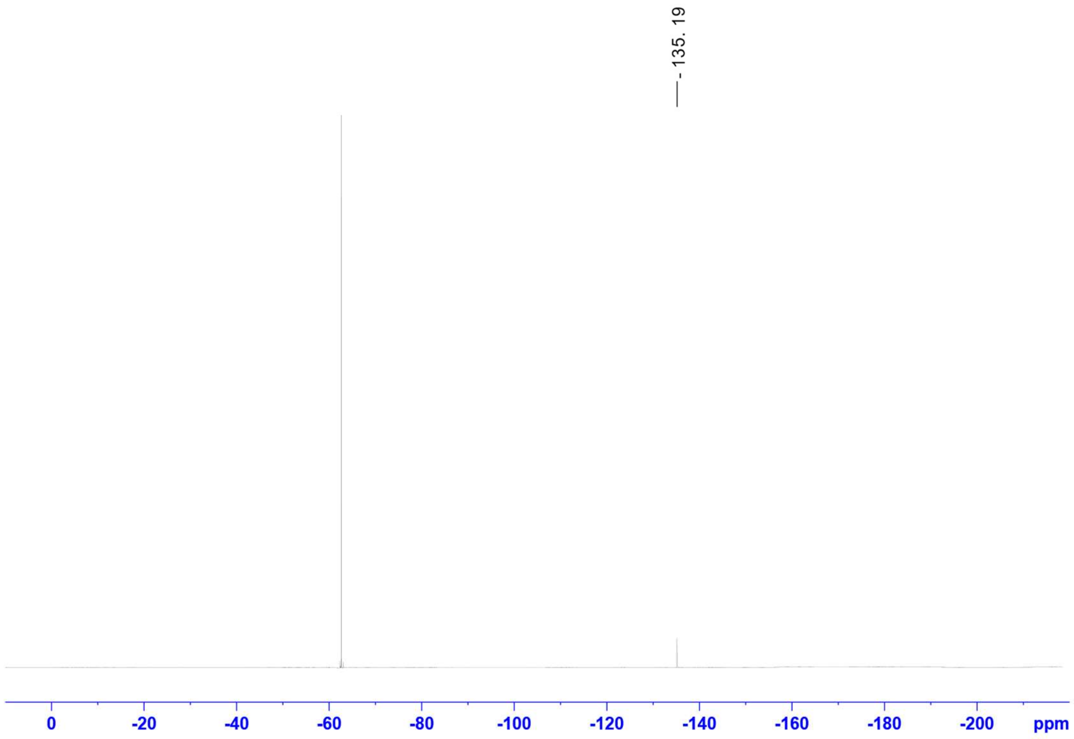

^{1}H NMR spectrum of *N*-(3,5-dichloro-2-fluoronitrophenyl)acetamide (400.13 MHz, $CDCl_3$)

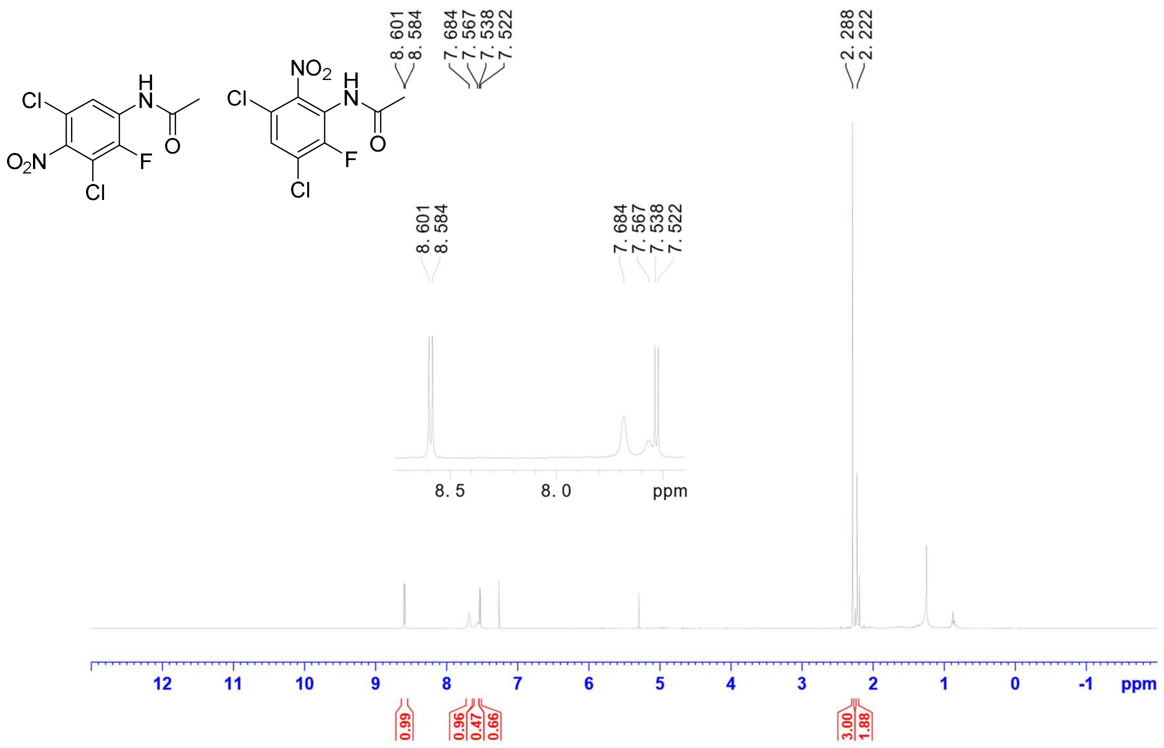

^{13}C{^{1}H} NMR spectrum of *N*-(3,5-dichloro-2-fluoronitrophenyl)acetamide (100.61 MHz, $CDCl_3$)

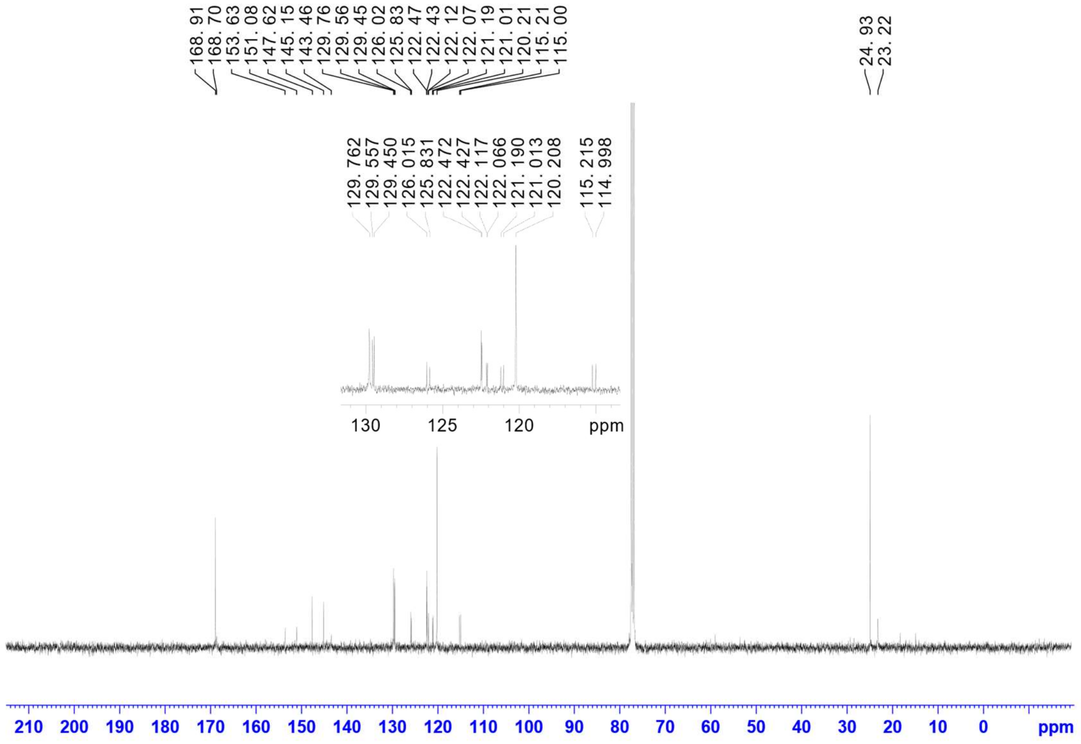

^{19}F NMR spectrum of *N*-(3,5-dichloro-2-fluoronitrophenyl)acetamide (376.46 MHz, $CDCl_3$)

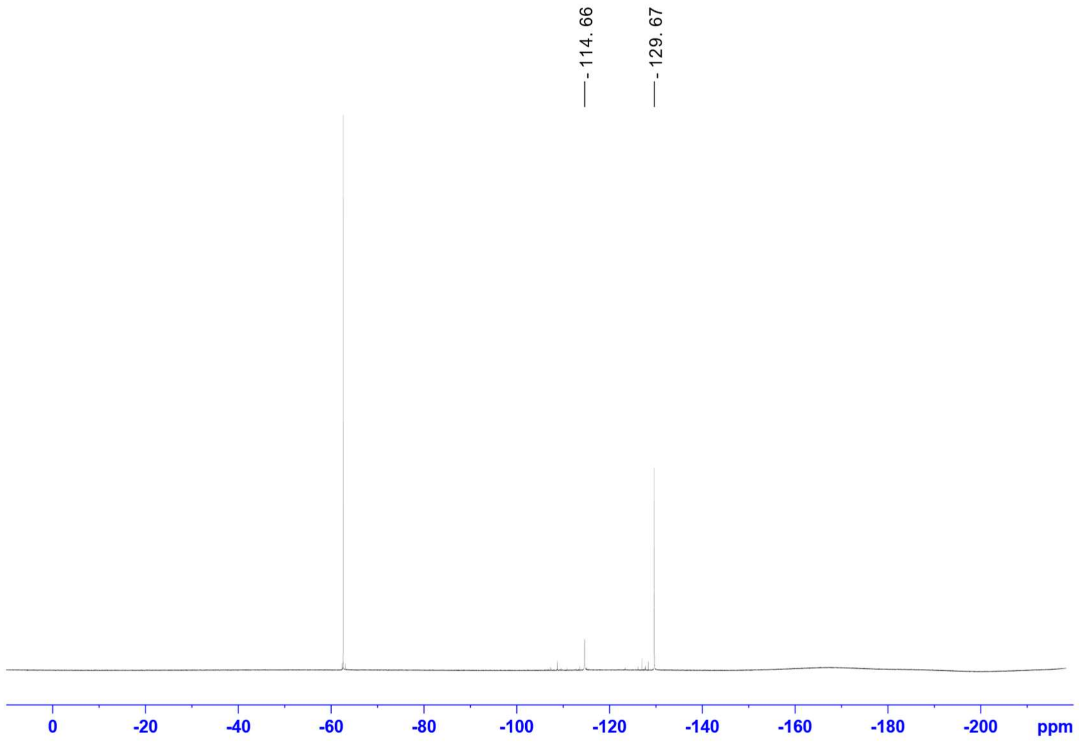

^{1}H NMR spectrum of **10** (400.13 MHz, $CDCl_3$)

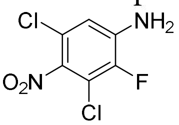

10

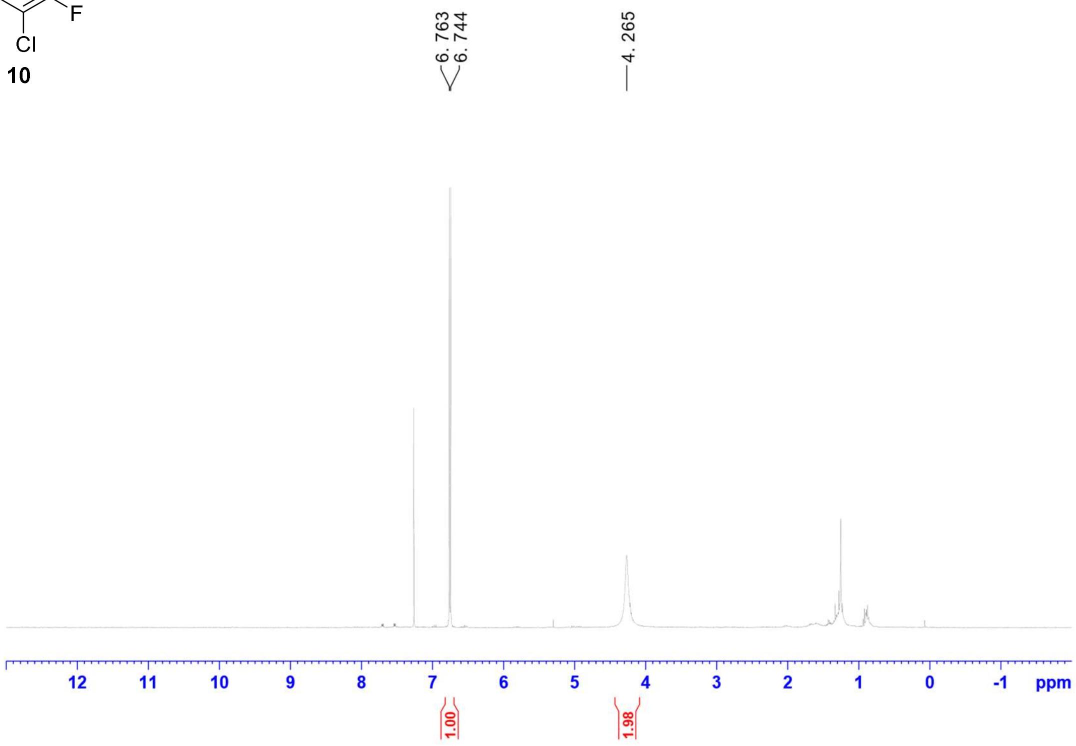

^{13}C{^{1}H} NMR spectrum of **10** (100.61 MHz, $CDCl_3$)

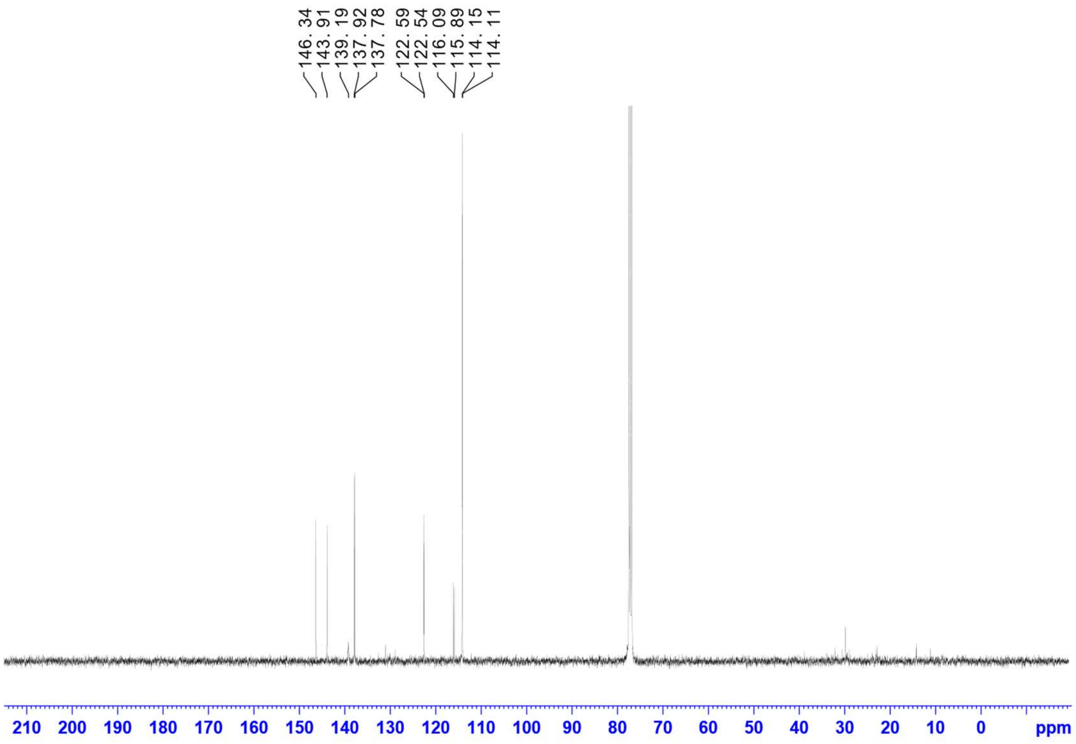

^{19}F NMR spectrum of **10** (376.46 MHz, $CDCl_3$)

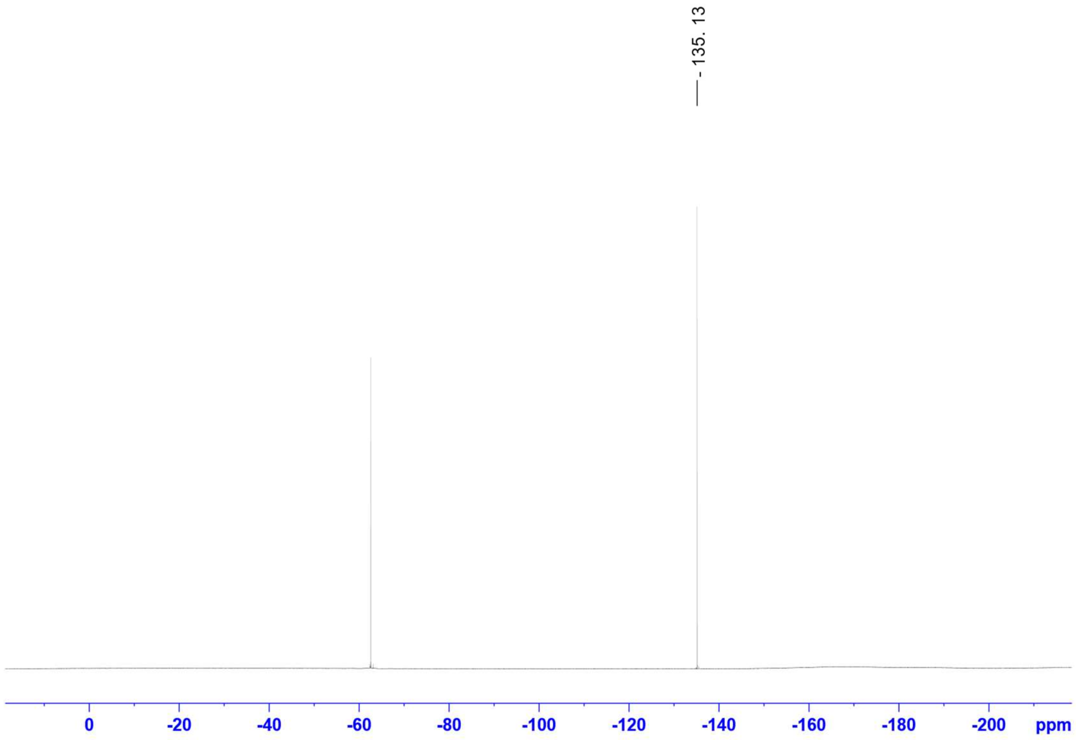

^{1}H NMR spectrum of **11** (400.13 MHz, $CDCl_3$)

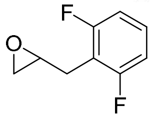

11

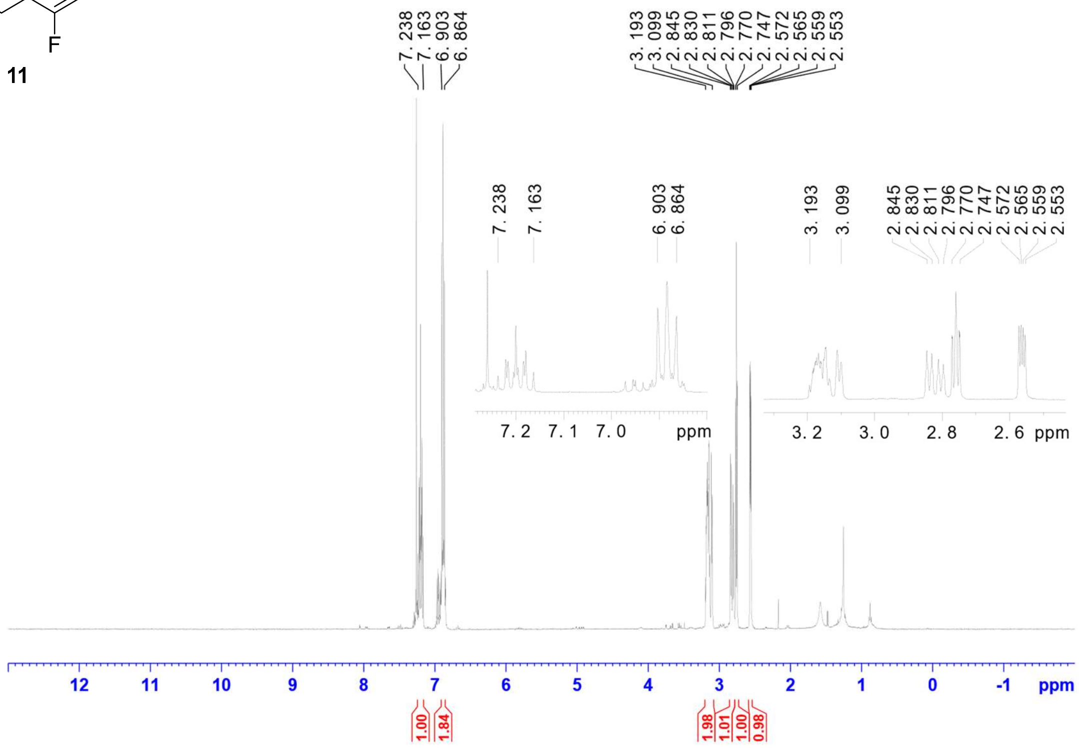

^{13}C{^{1}H} NMR spectrum of **11** (100.61 MHz, $CDCl_3$)

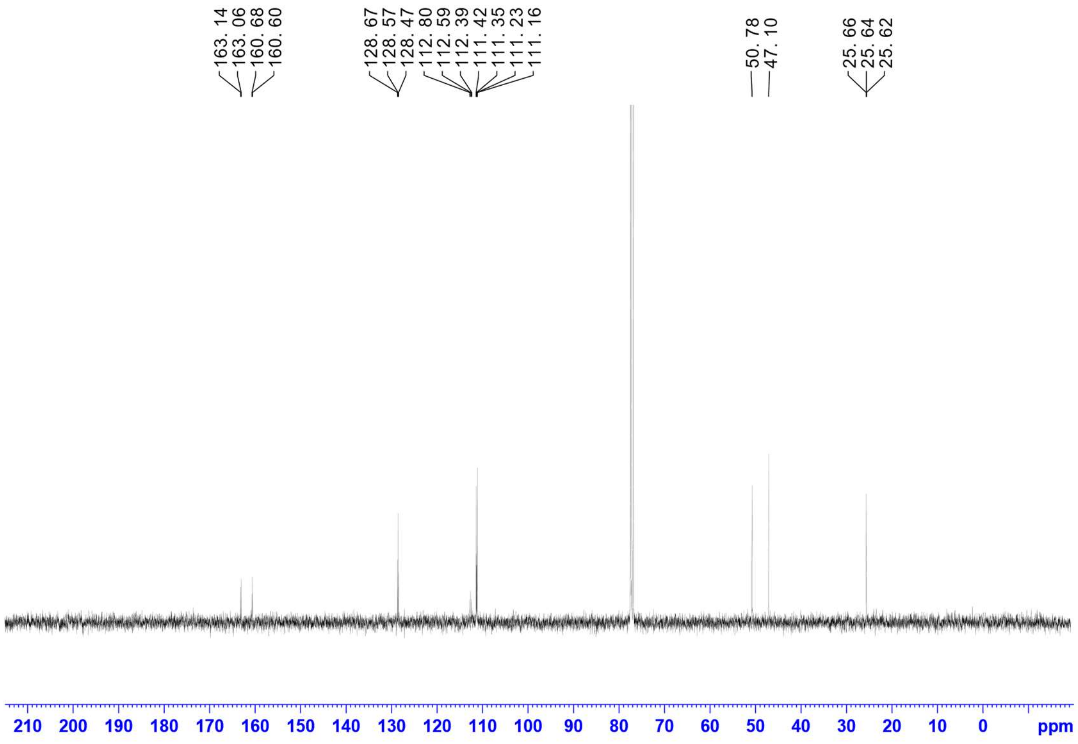

^{19}F NMR spectrum of **11** (376.46 MHz, $CDCl_3$)

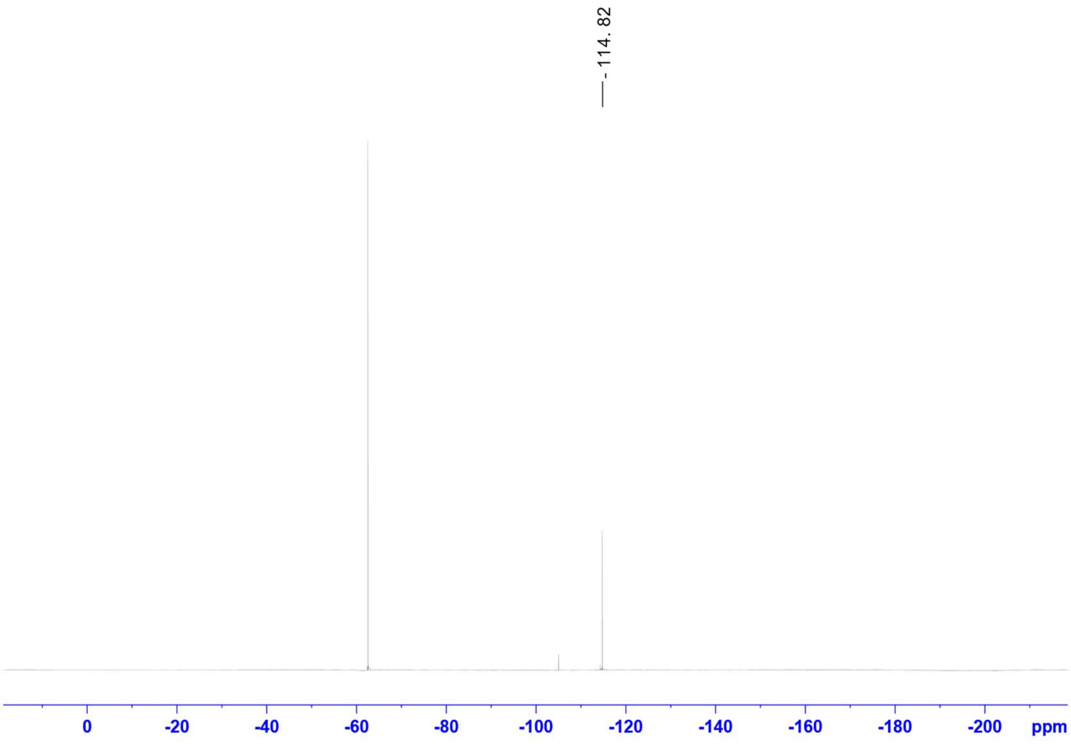

^{1}H NMR spectrum of 2-bromo-1,3-difluoro-4-nitrobenzene (400.61 MHz, $CDCl_3$)

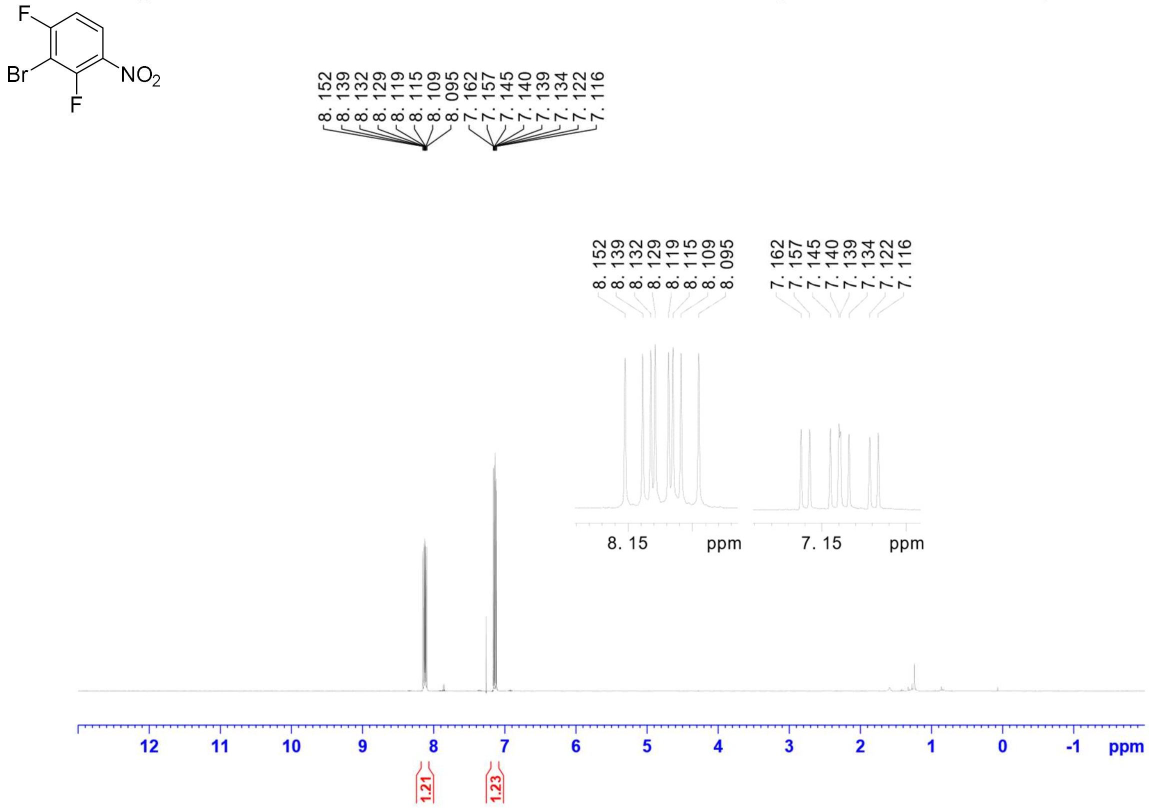

^{13}C{^{1}H} NMR spectrum of 2-bromo-1,3-difluoro-4-nitrobenzene (100.61 MHz, $CDCl_3$)

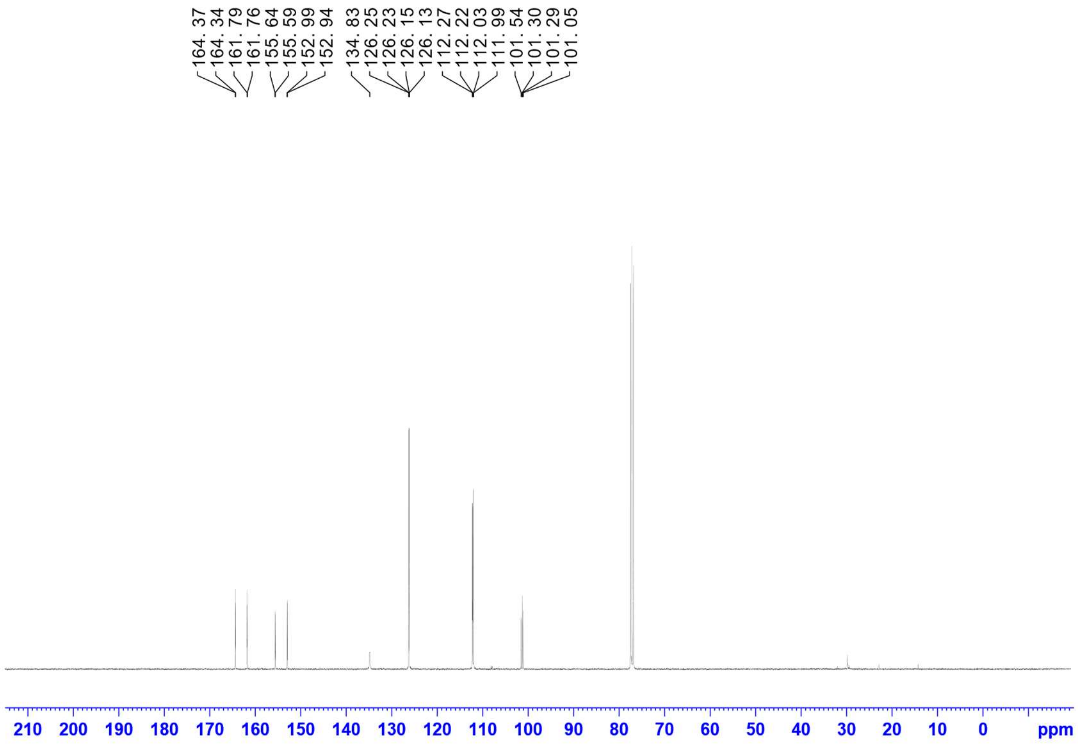

^{19}F NMR spectrum of 2-bromo-1,3-difluoro-4-nitrobenzene (376.46 MHz, $CDCl_3$)

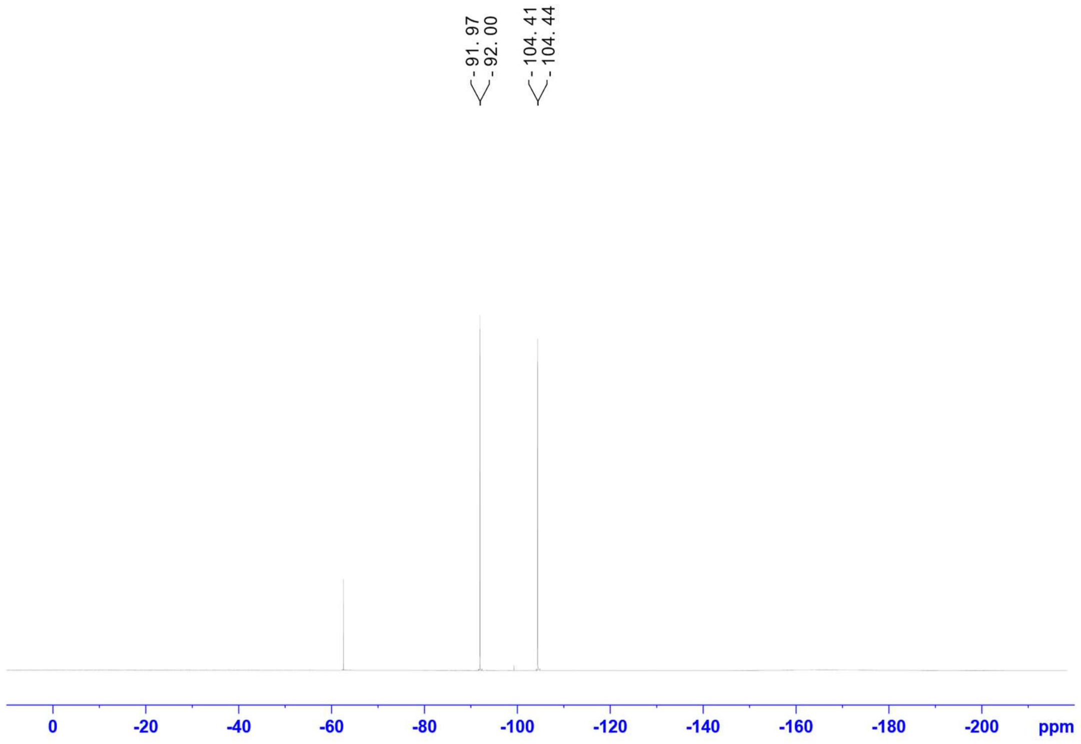

^{1}H NMR spectrum of 2-allyl-1,3-difluoro-4-nitrobenzene (400.13 MHz, $CDCl_3$)

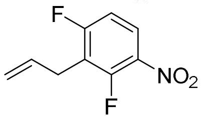

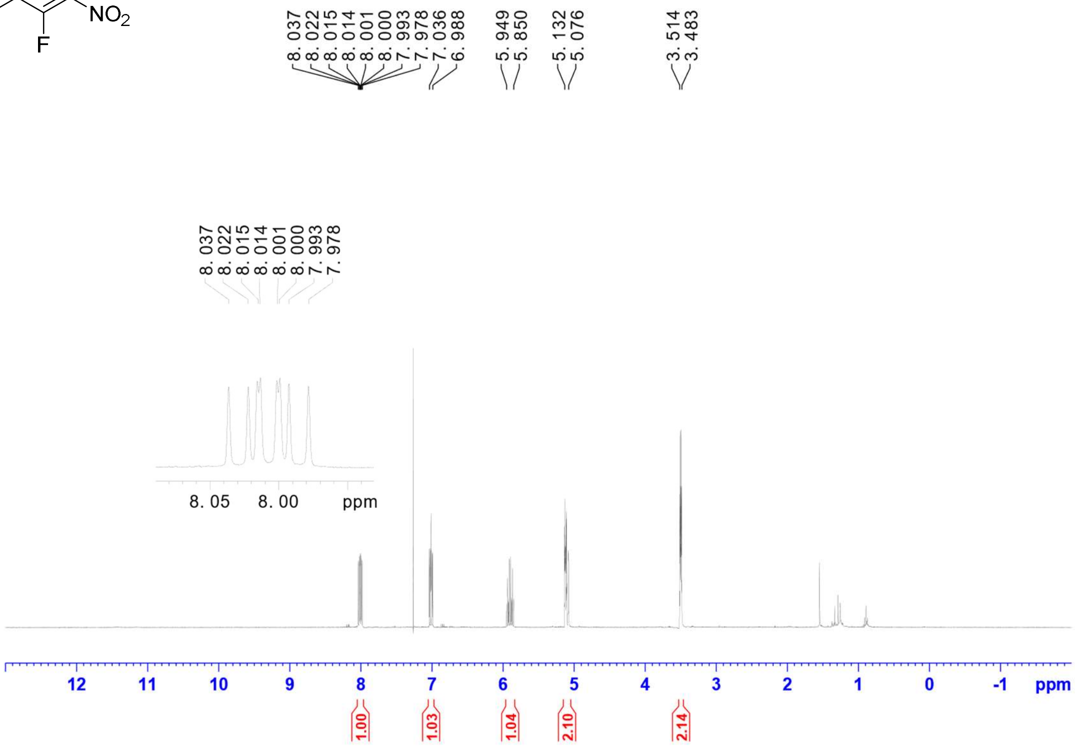

^{13}C{^{1}H} NMR spectrum of 2-allyl-1,3-difluoro-4-nitrobenzene (100.61 MHz, $CDCl_3$)

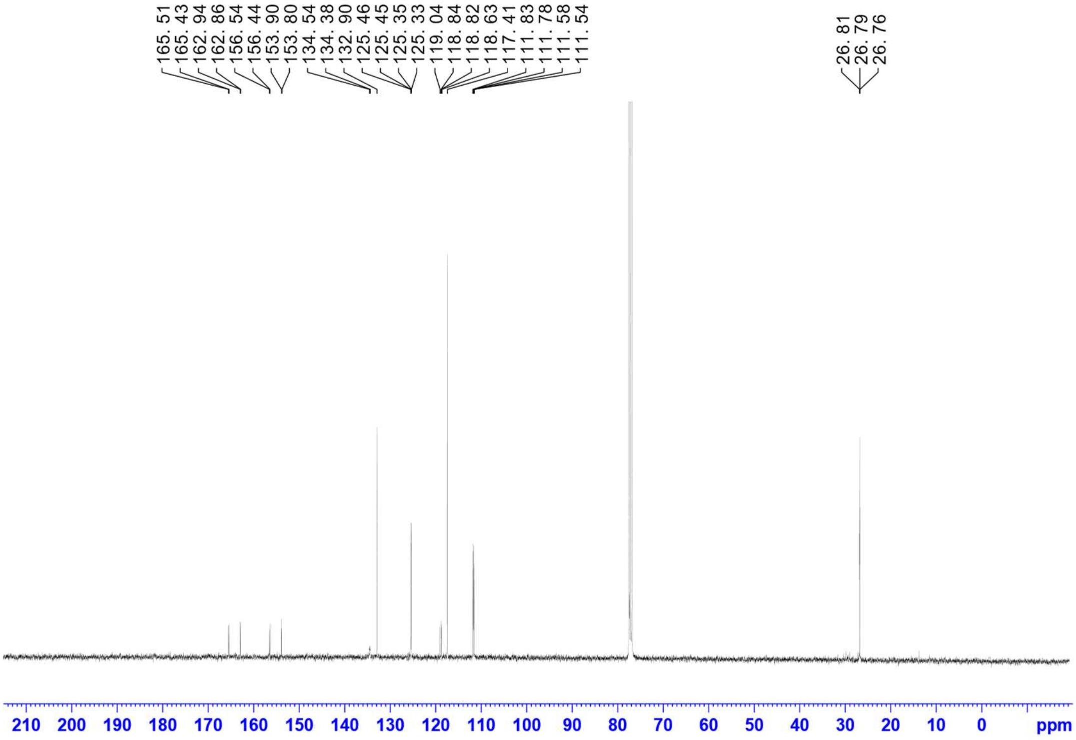

^{19}F NMR spectrum of 2-allyl-1,3-difluoro-4-nitrobenzene (376.46 MHz, $CDCl_3$)

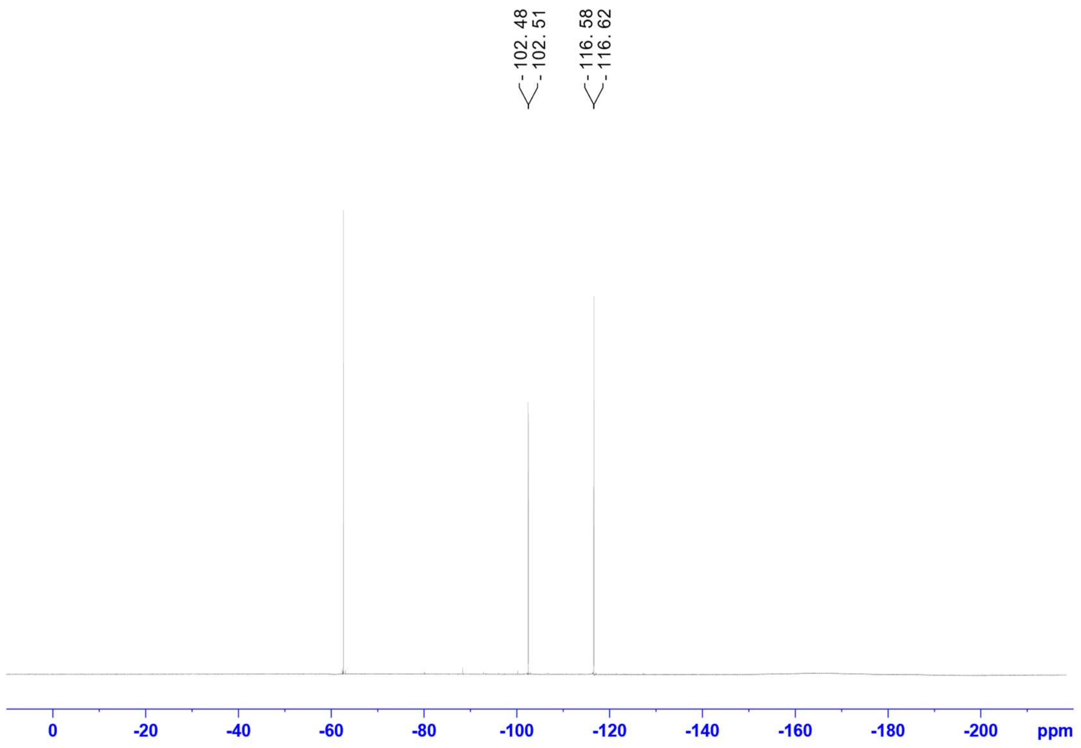

^{1}H NMR spectrum of **12** (400.13 MHz, $CDCl_3$)

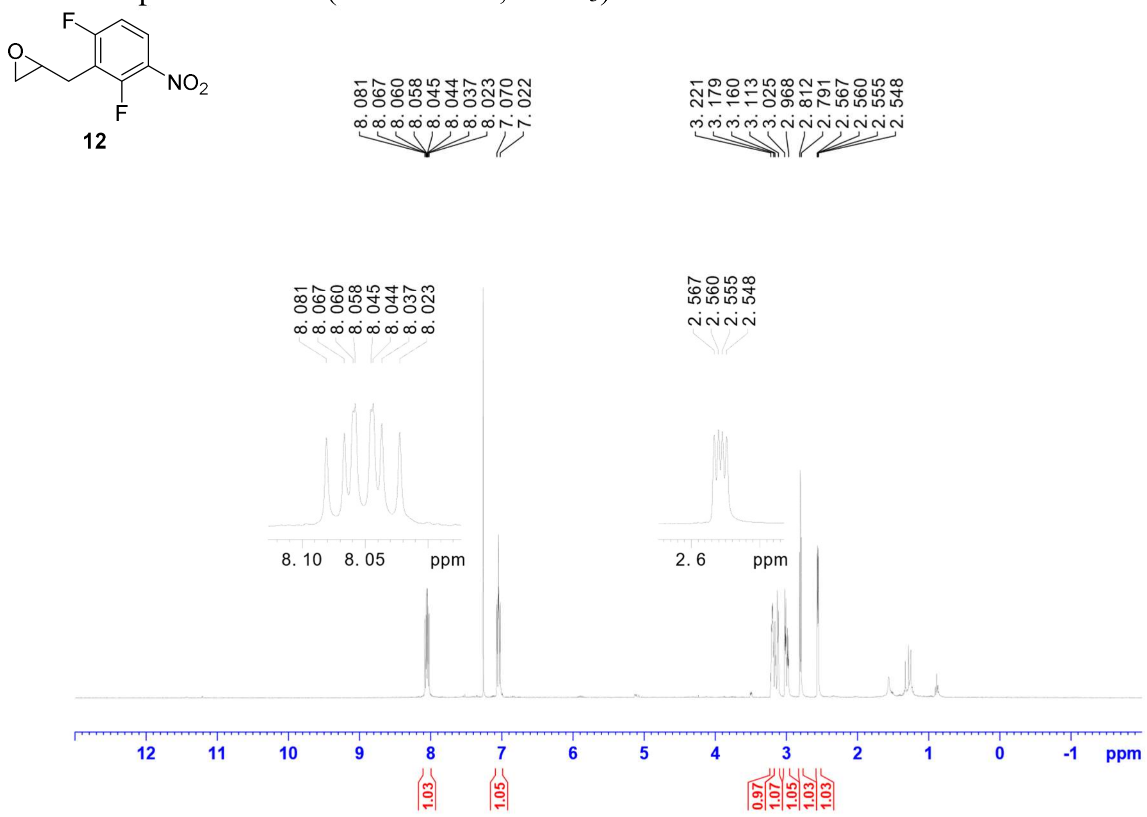

^{13}C{^{1}H} NMR spectrum of **12** (100.61 MHz, $CDCl_3$)

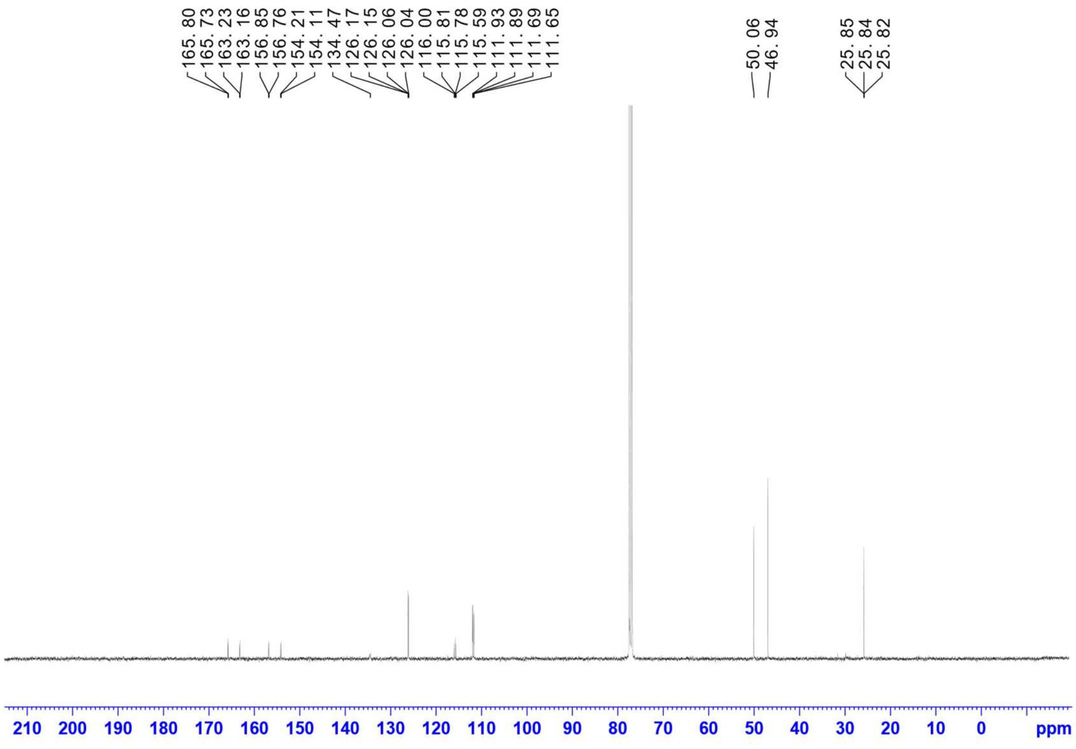

^{19}F NMR spectrum of **12** (376.46 MHz, $CDCl_3$)

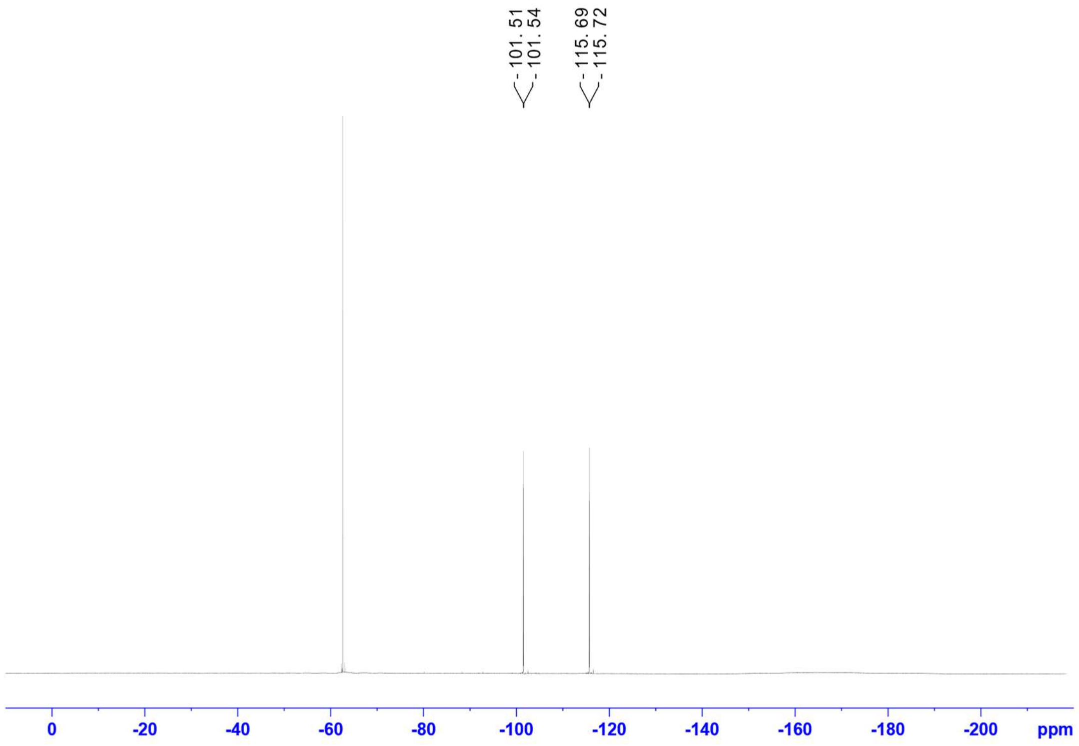

^{1}H NMR spectrum of 1-allyl-4-chlorobenzene (400.13 MHz, $CDCl_3$)

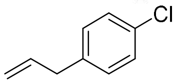

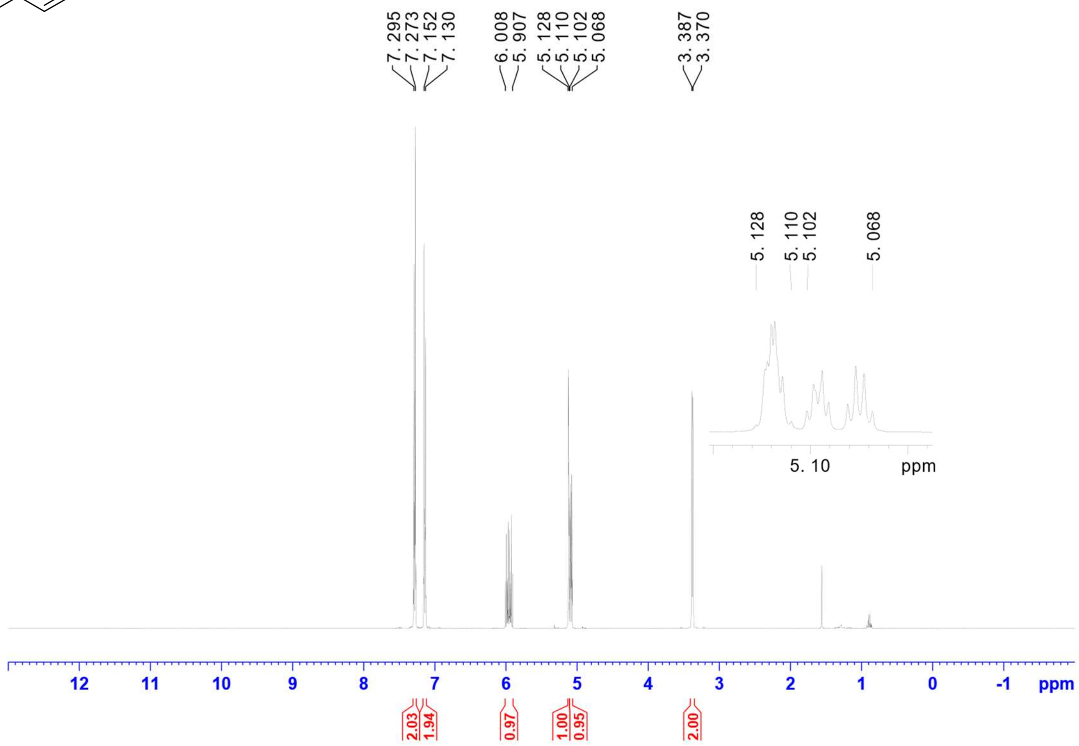

^{13}C{^{1}H} NMR spectrum of 1-allyl-4-chlorobenzene (100.61 MHz, $CDCl_3$)

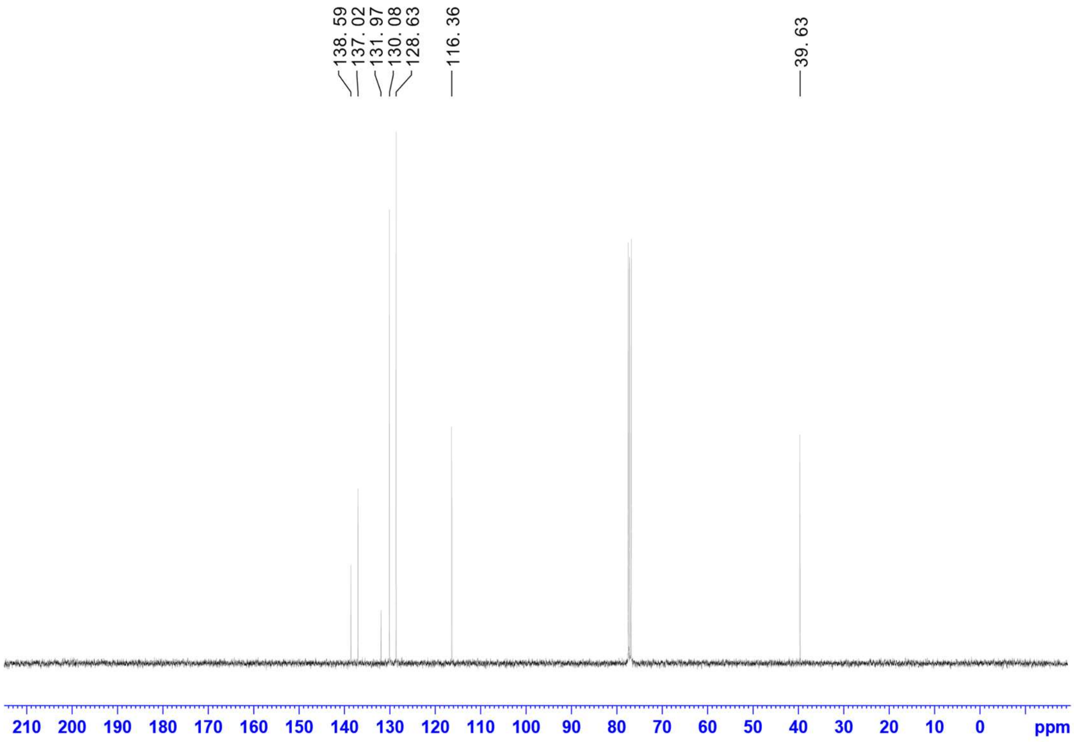

^{1}H NMR spectrum of **13** (400.13 MHz, $CDCl_3$)

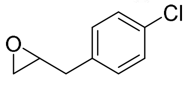

13

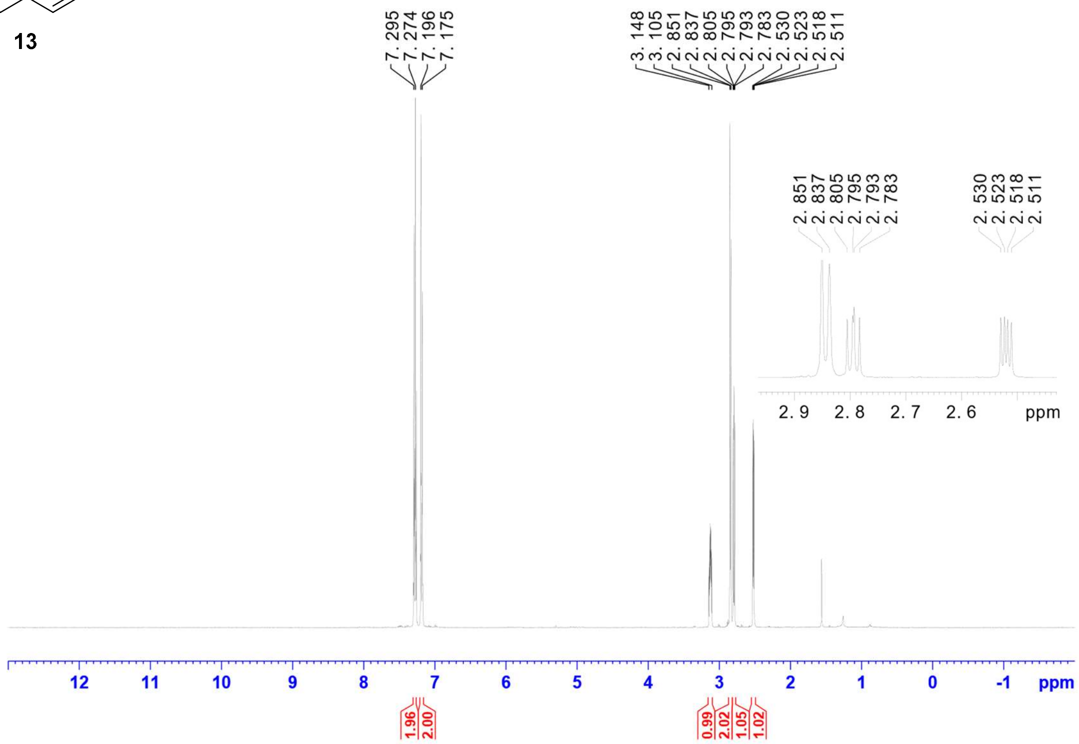

^{13}C{^{1}H} NMR spectrum of **13** (100.61 MHz, $CDCl_3$)

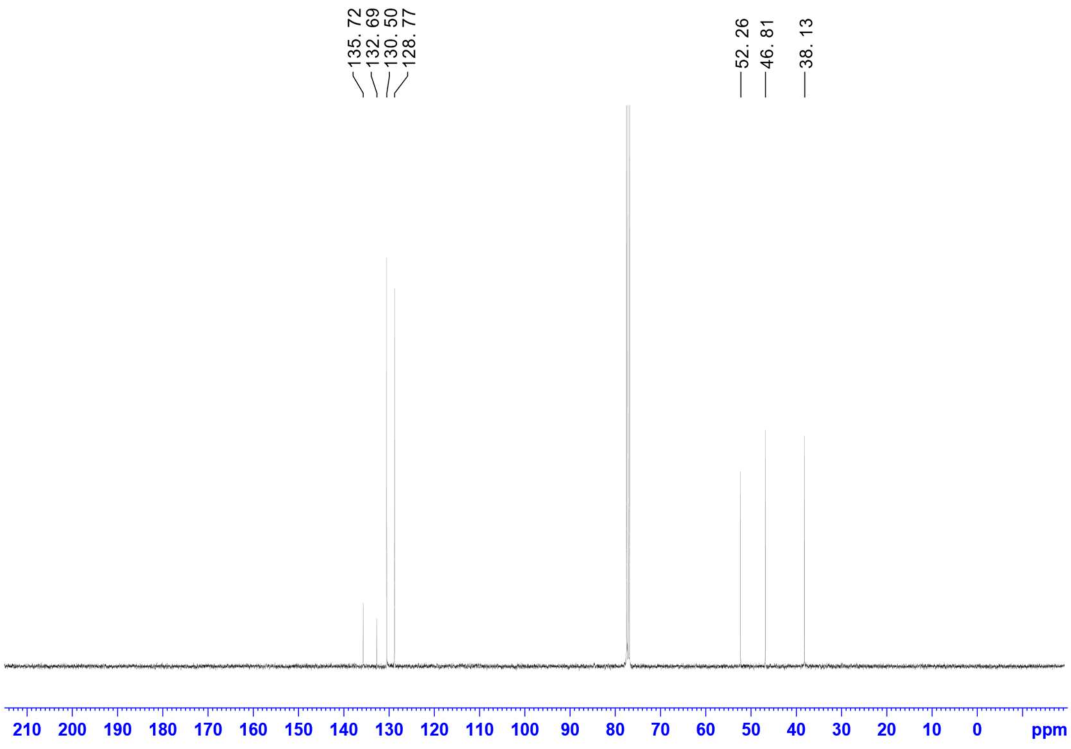

^{1}H NMR spectrum of **14a** (850.13 MHz, CD_3CN)

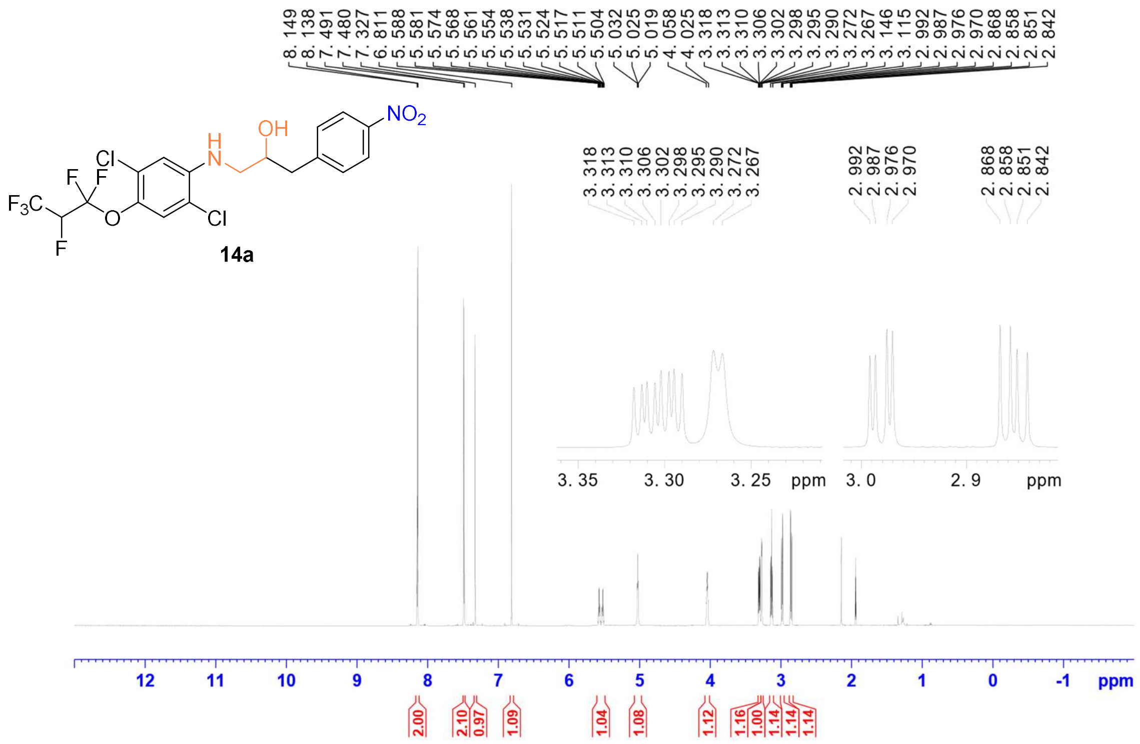

^{13}C{^{1}H} NMR spectrum of **14a** (213.77 MHz, CD_3CN)

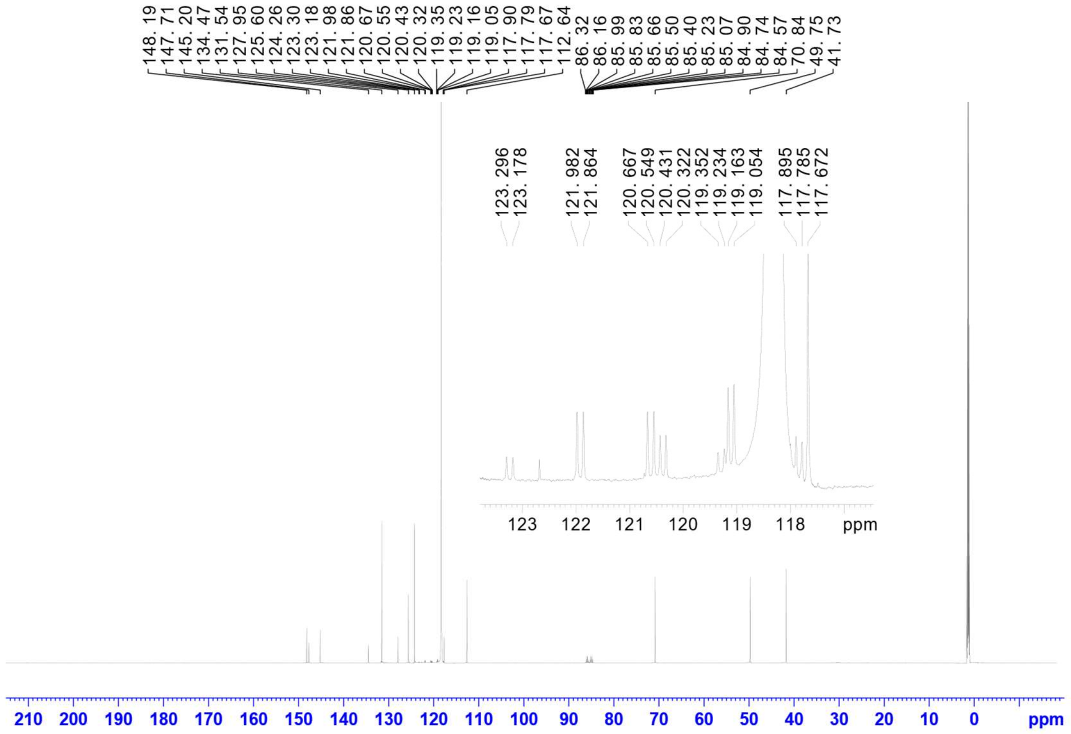

^{19}F NMR spectrum of **14a** (376.46 MHz, CD_3CN)

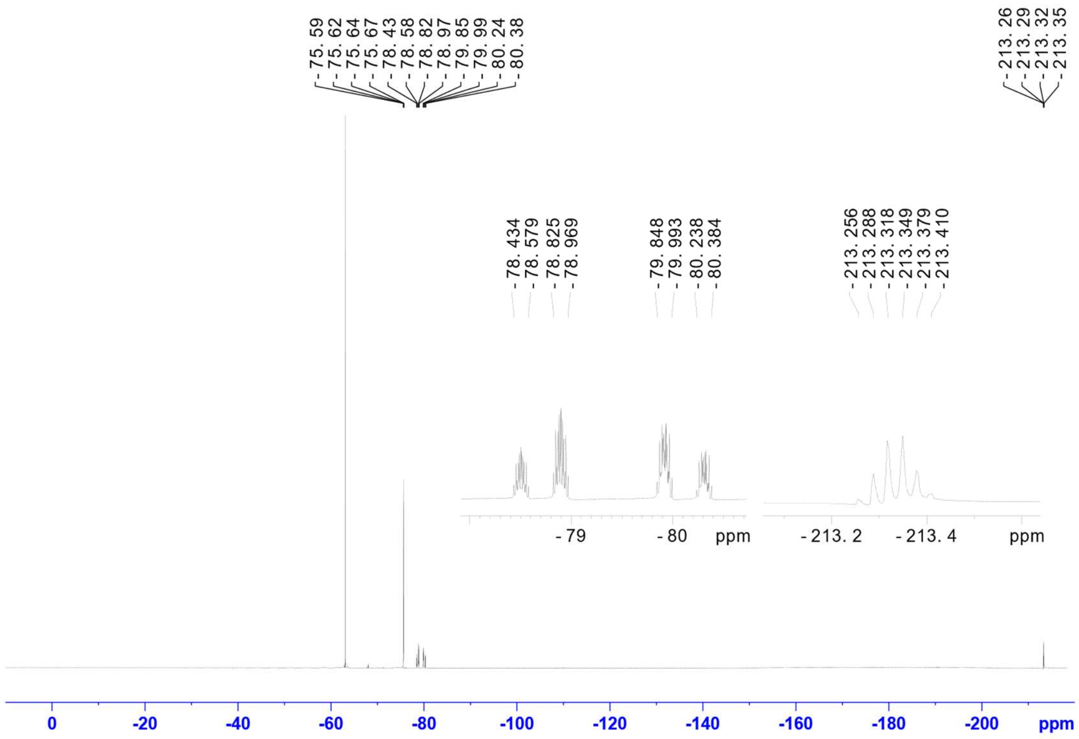

^{1}H NMR spectrum of **14b** (400.13 MHz, CD_3CN)

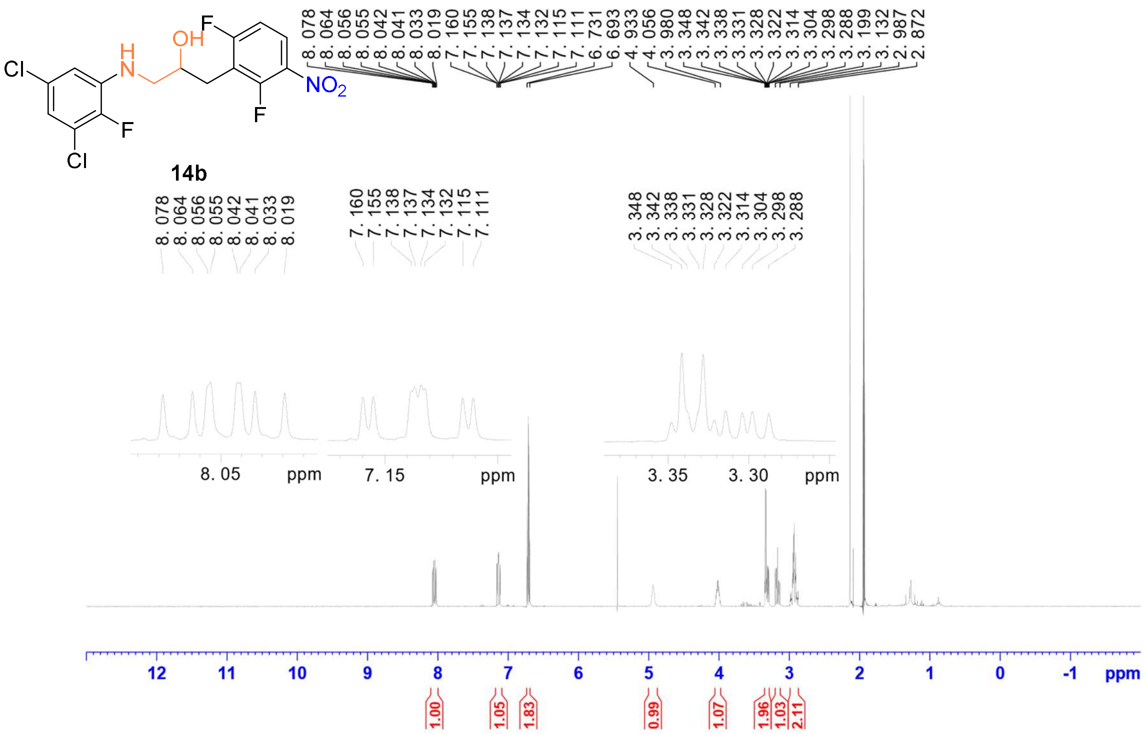

^{13}C{^{1}H} NMR spectrum of **14b** (100.61 MHz, CD_3CN)

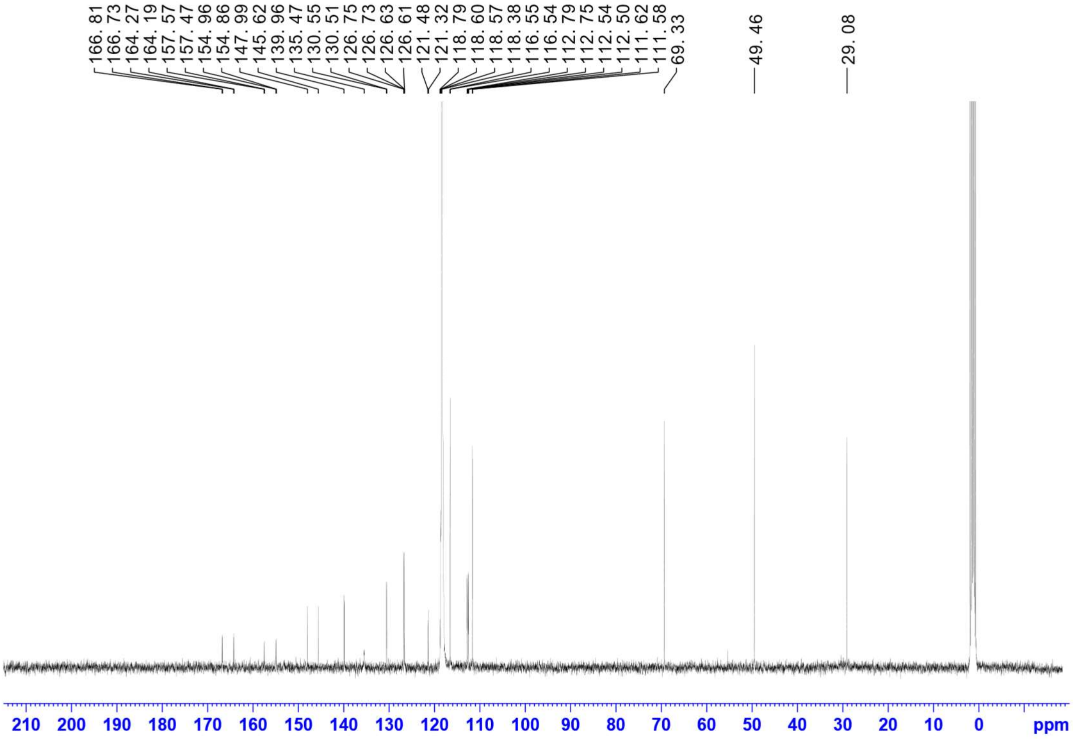

^{19}F NMR spectrum of **14b** (376.46 MHz, CD_3CN)

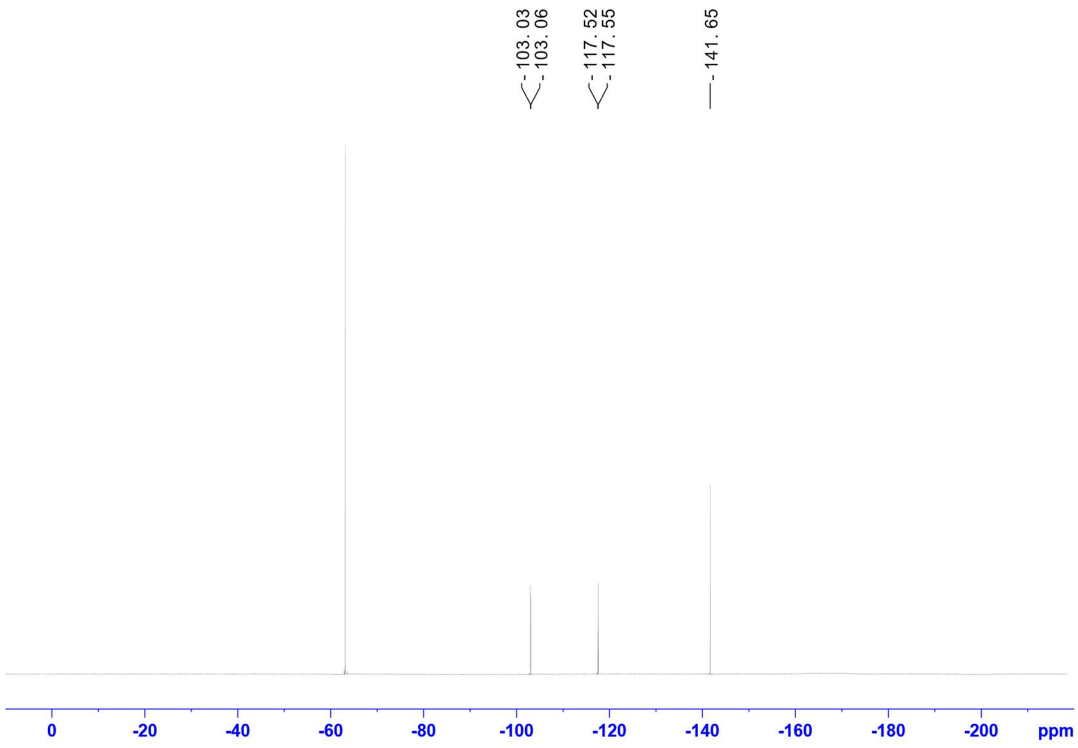

^{1}H NMR spectrum of **14c** (400.13 MHz, CD_3CN)

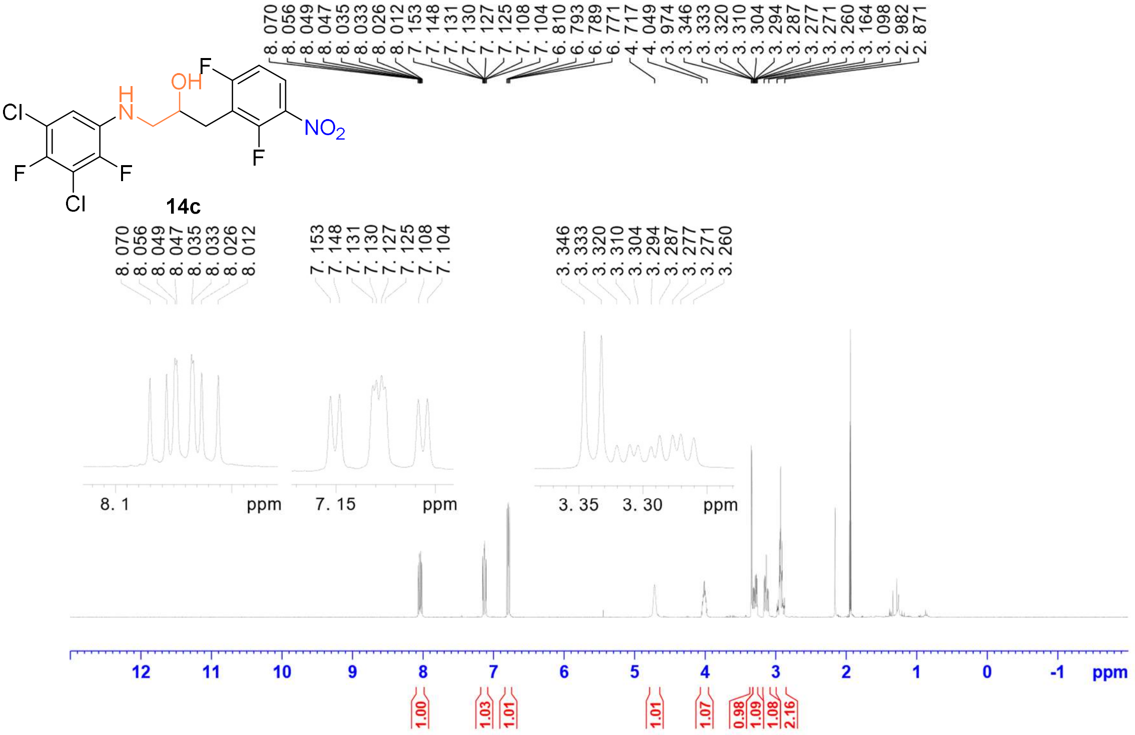

$^{13}C\{^{1}H\}$ NMR spectrum of **14c** (100.61 MHz, CD_3CN)

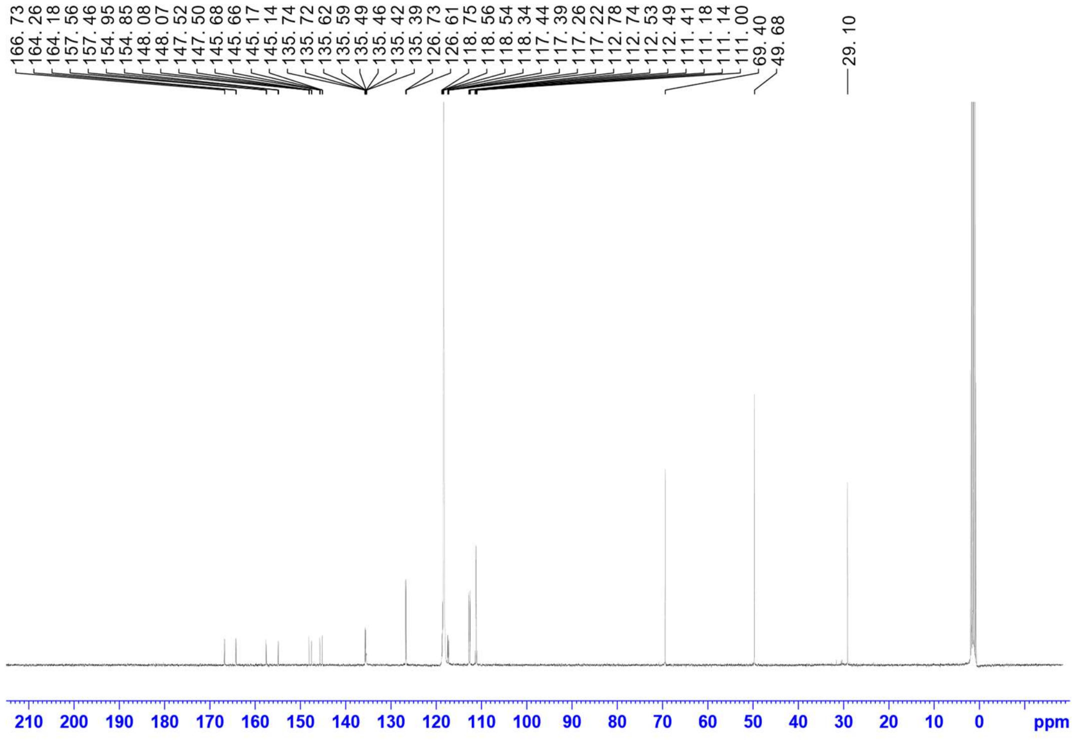

^{19}F NMR spectrum of **14c** (376.46 MHz, CD_3CN)

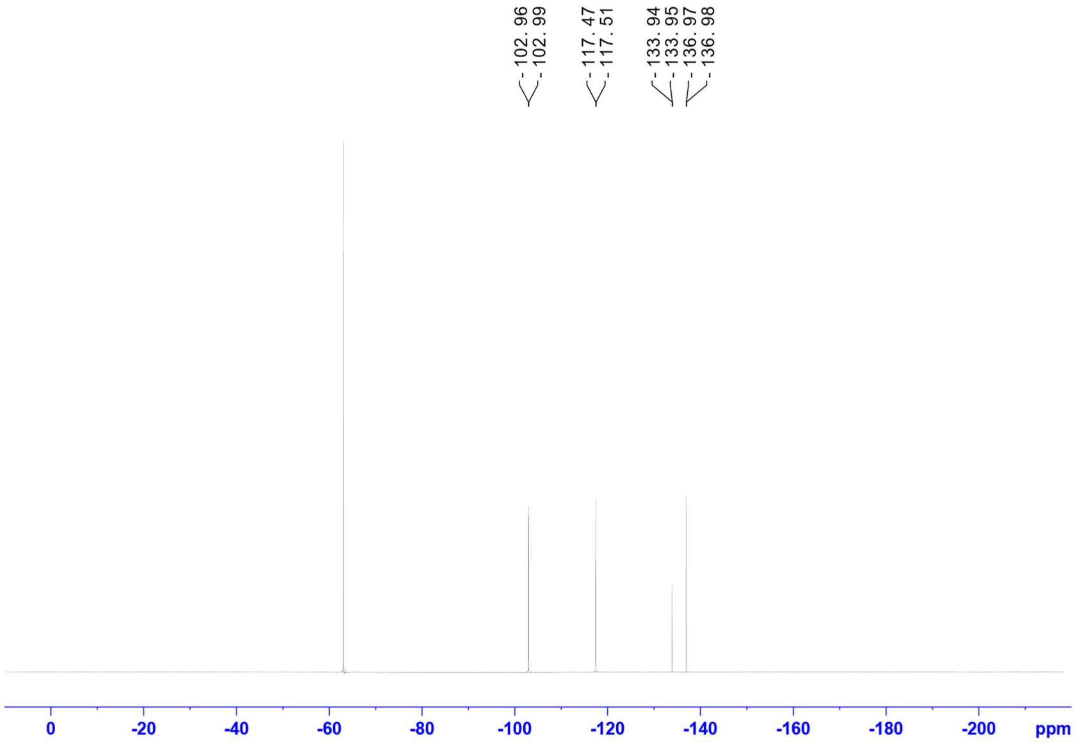

^{1}H NMR spectrum of **14d** (850.13 MHz, CD_3CN)

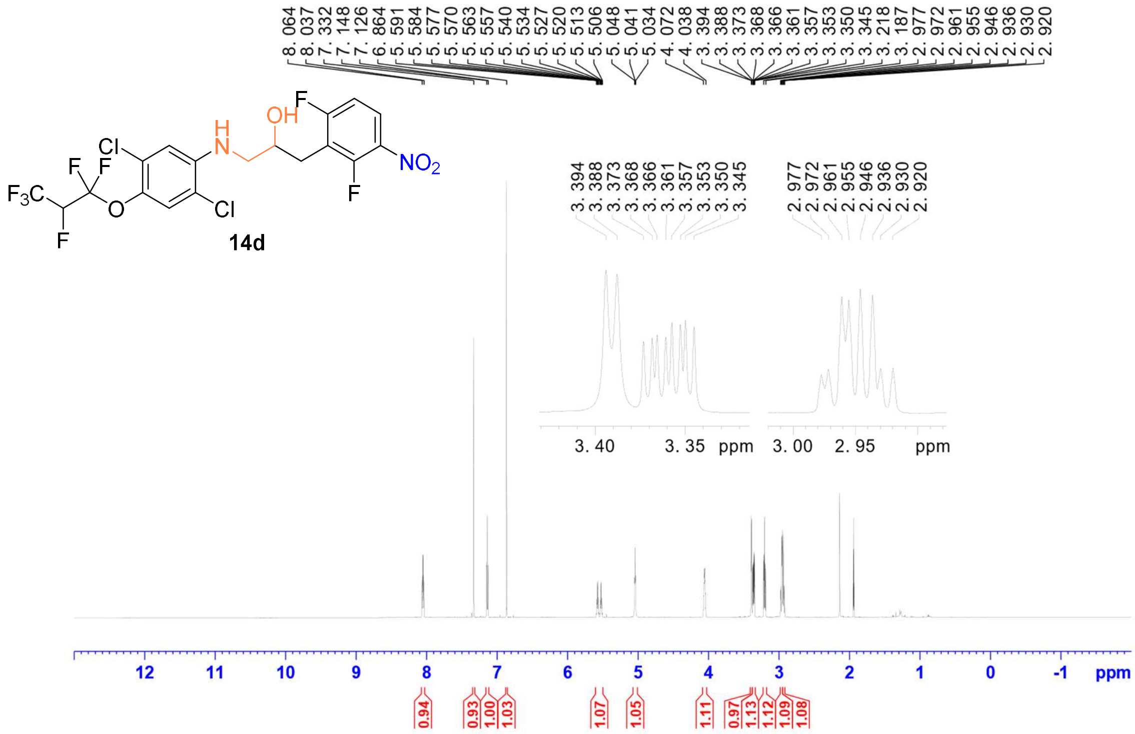

^{13}C{^{1}H} NMR spectrum of **14d** (213.77 MHz, CD_3CN)

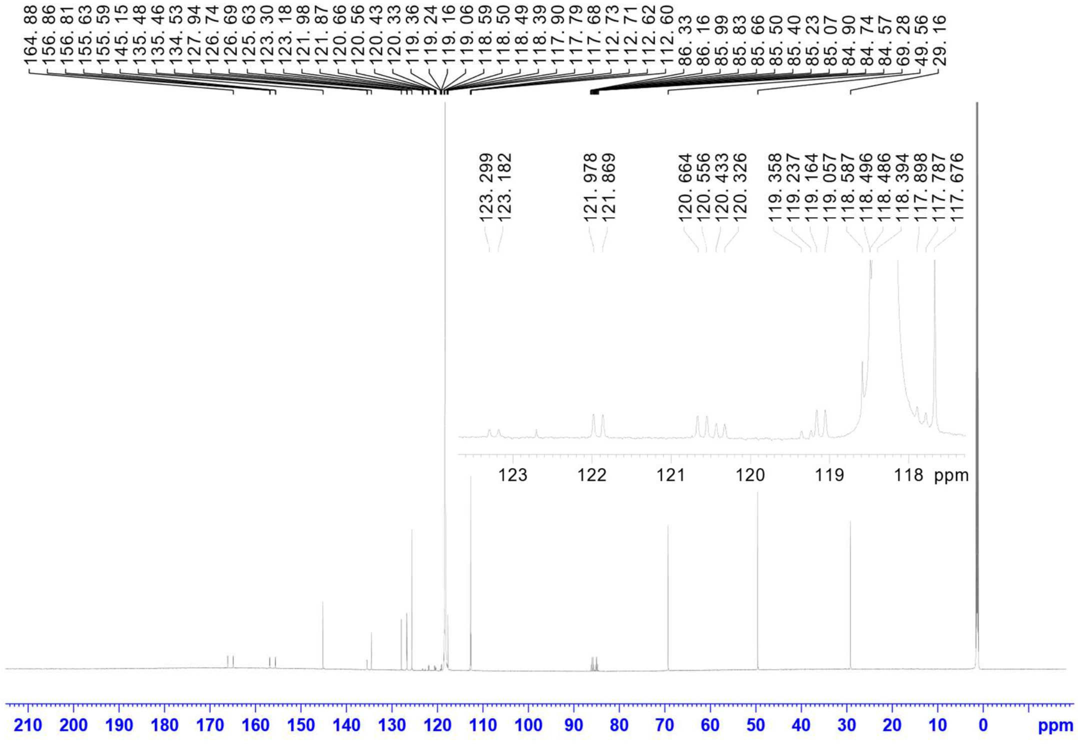

^{19}F NMR spectrum of **14d** (376.46 MHz, CD_3CN)

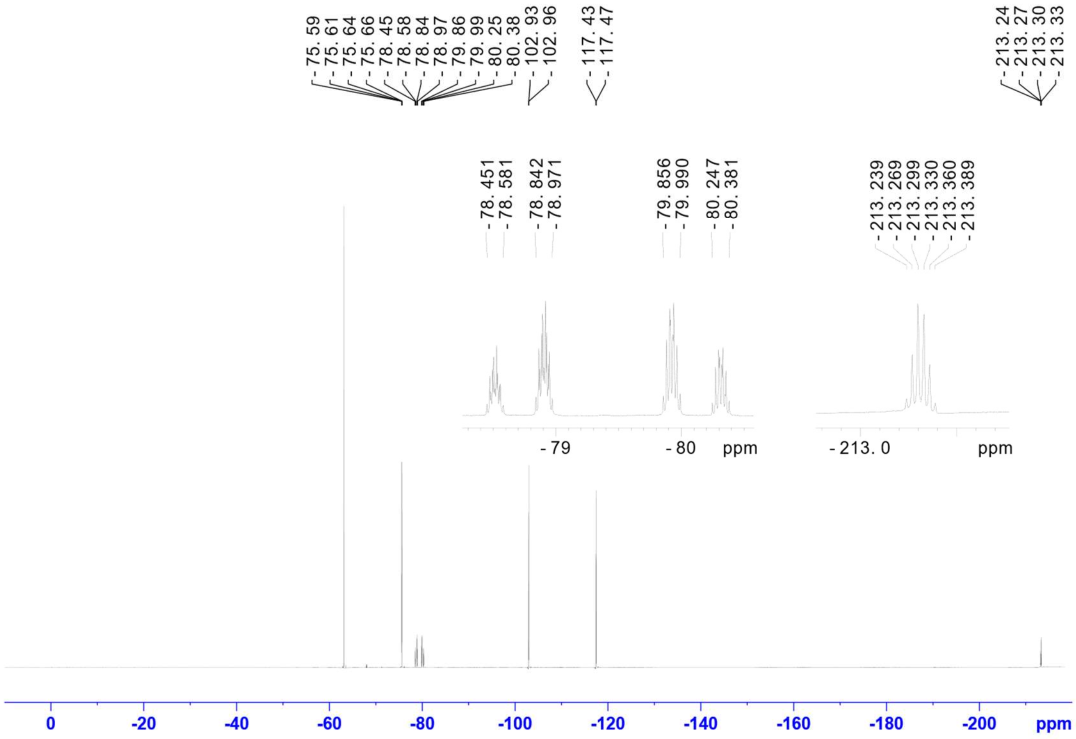

^{1}H NMR spectrum of **14e** (400.13 MHz, CD_3CN)

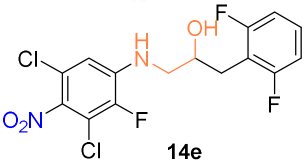

14e

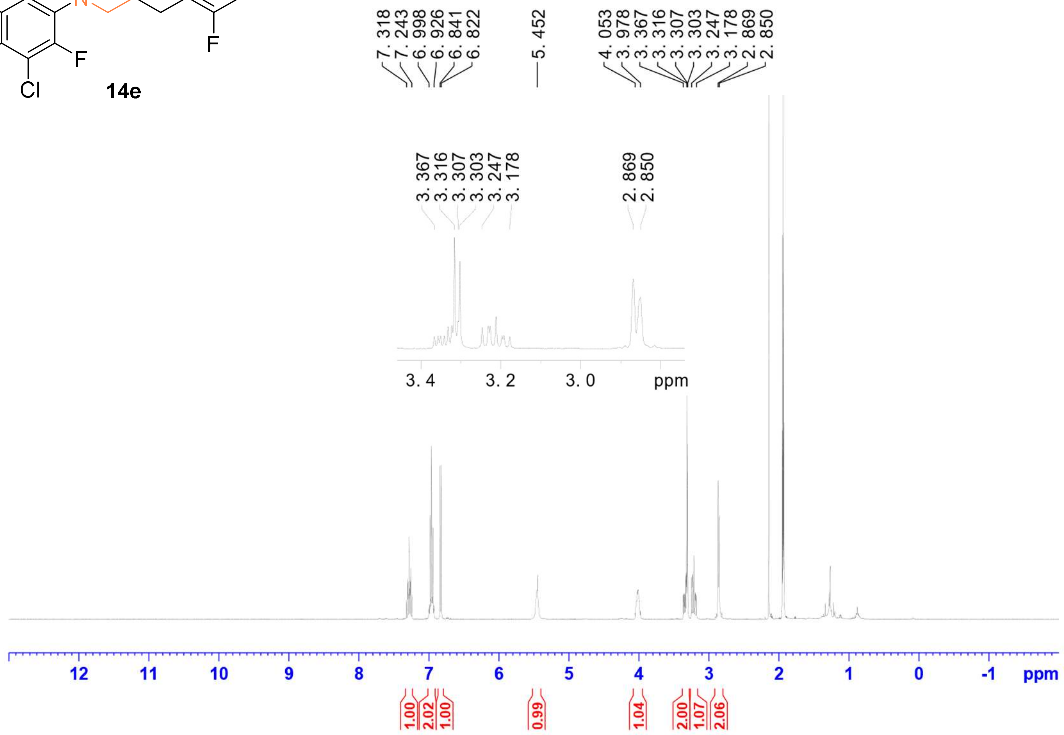

^{13}C{^{1}H} NMR spectrum of **14e** (100.61 MHz, CD_3CN)

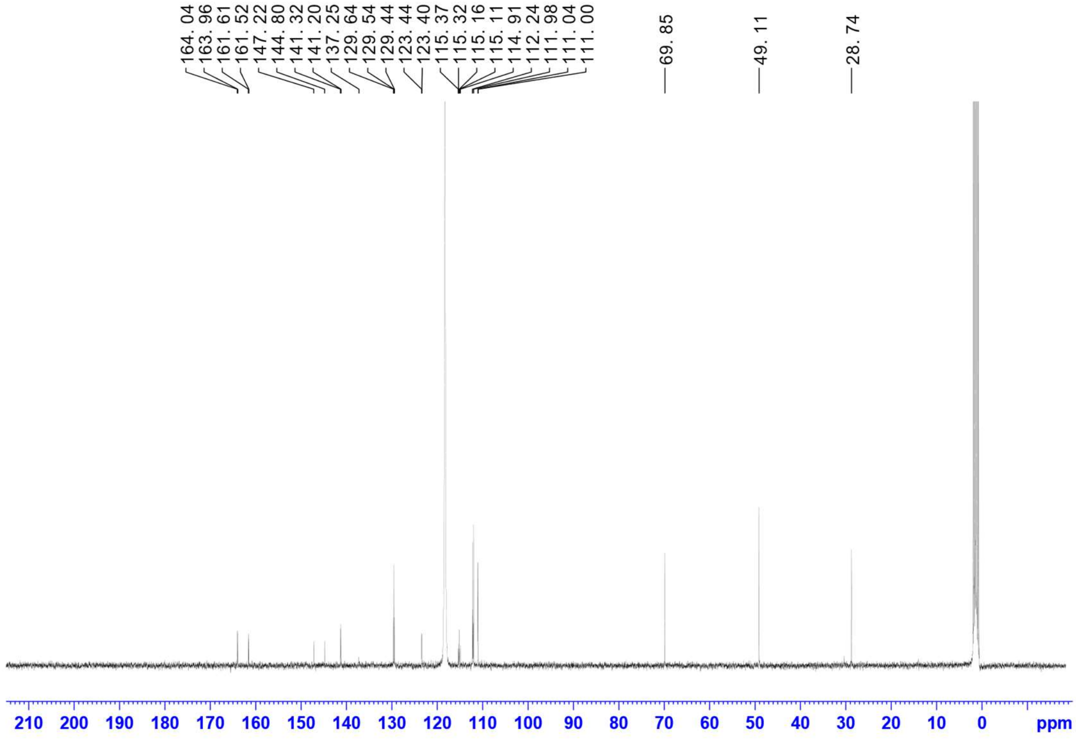

^{19}F NMR spectrum of **14e** (376.46 MHz, CD_3CN)

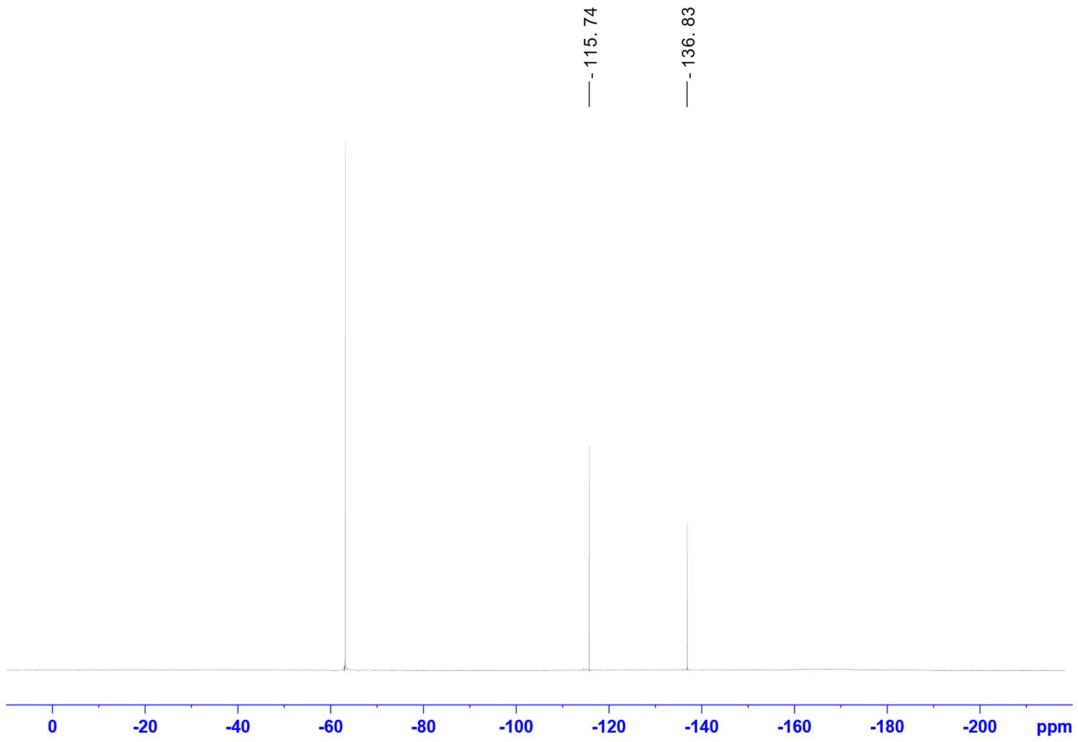

^{1}H NMR spectrum of **14f** (400.13 MHz, CD_3CN)

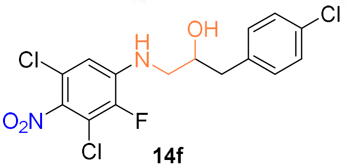

14f

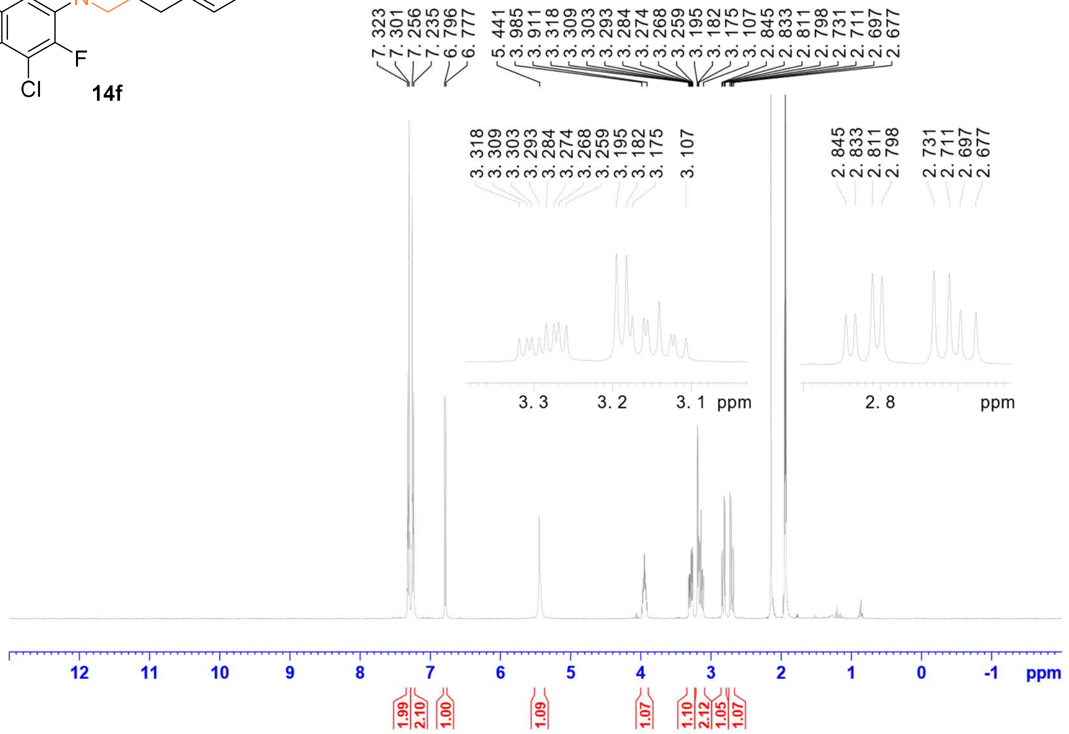

^{13}C{^{1}H} NMR spectrum of **14f** (100.61 MHz, CD_3CN)

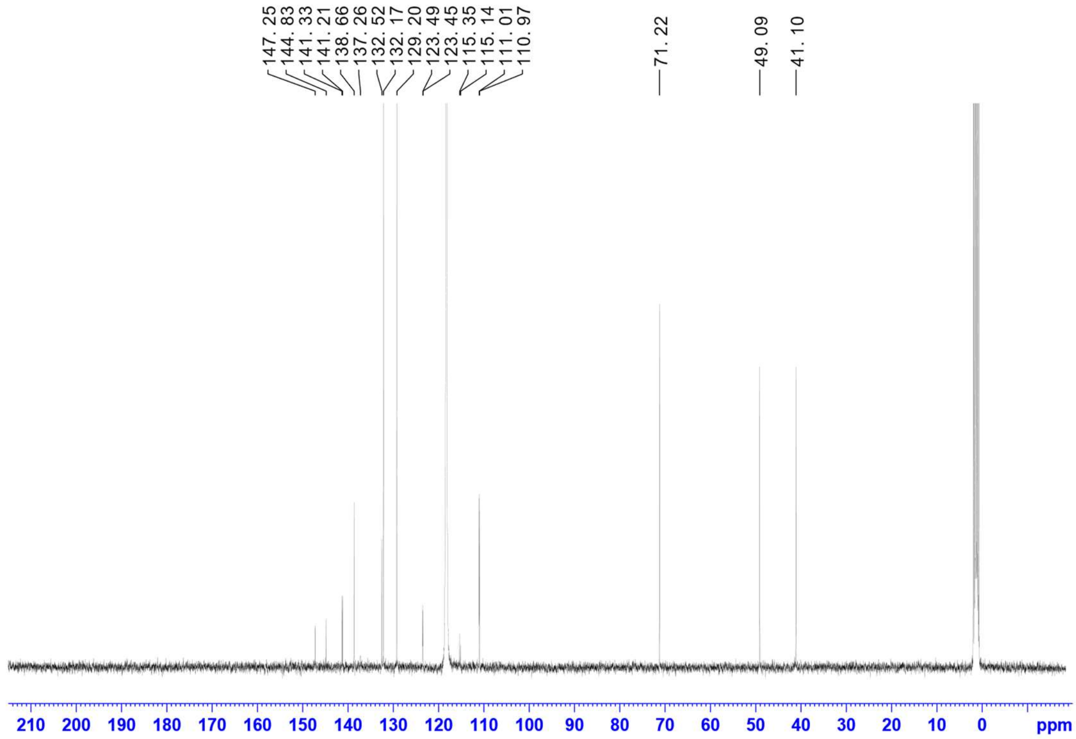

^{19}F NMR spectrum of **14f** (376.46 MHz, CD_3CN)

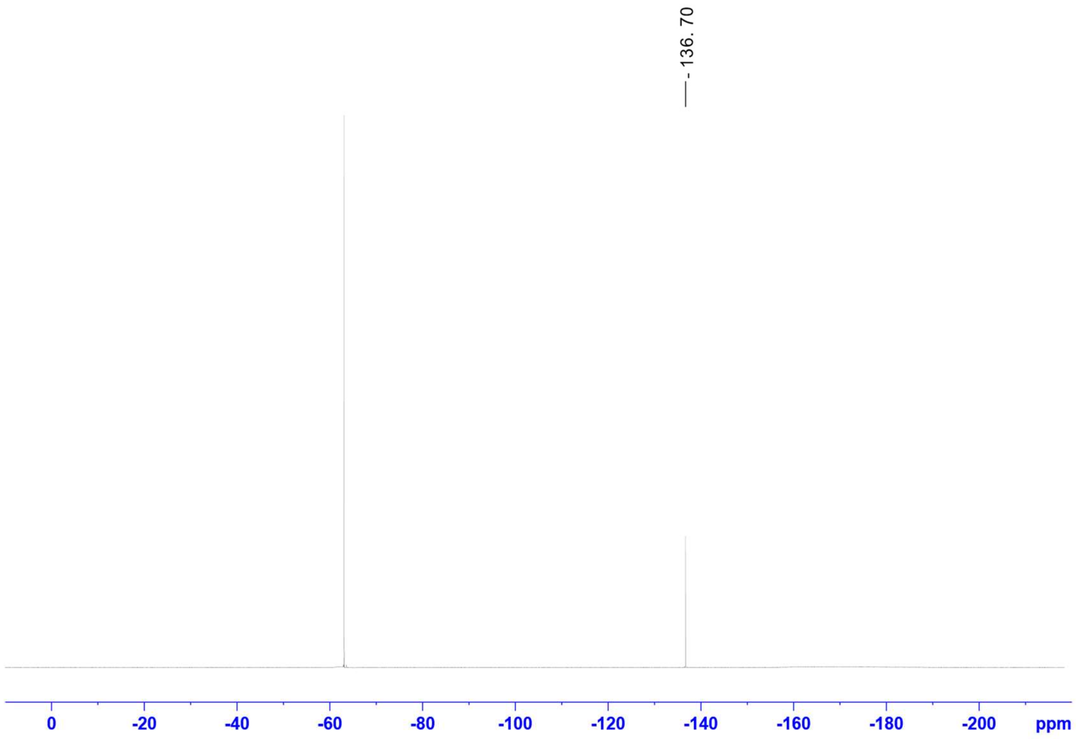

^{1}H NMR spectrum of **14g** (400.13 MHz, CD_3CN)

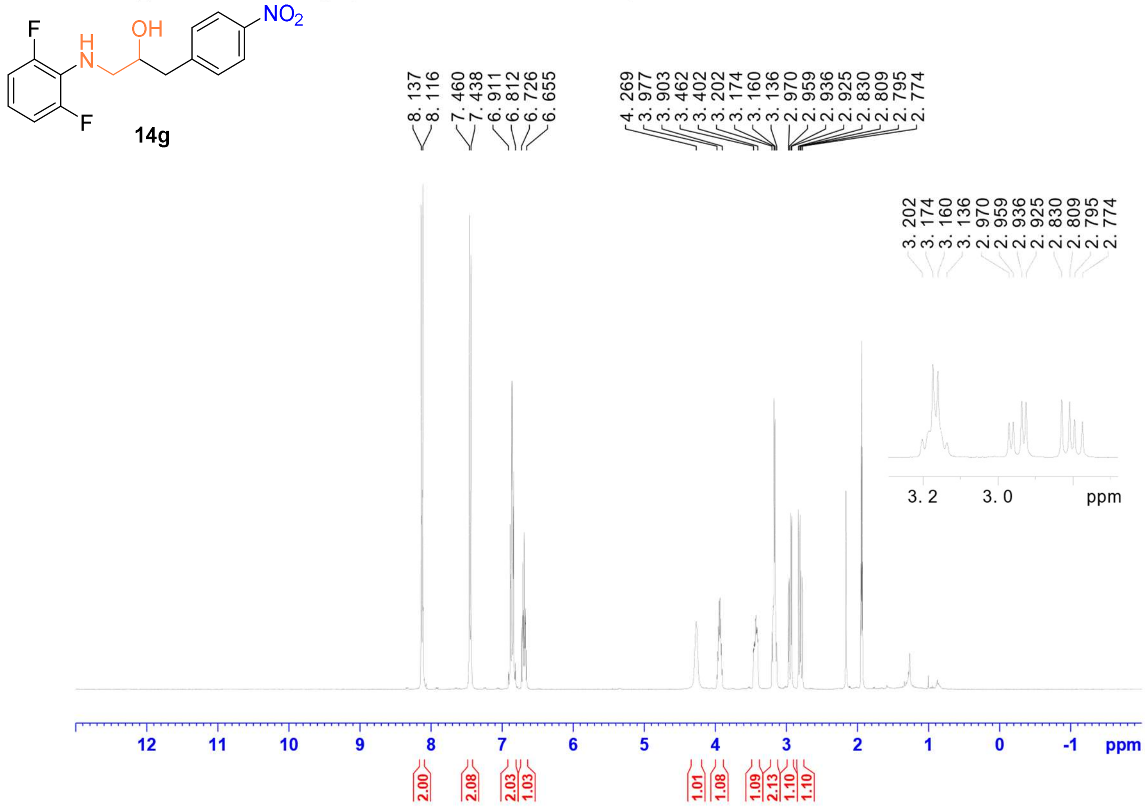

$^{13}C\{^{1}H\}$ NMR spectrum of **14g** (100.61 MHz, CD_3CN)

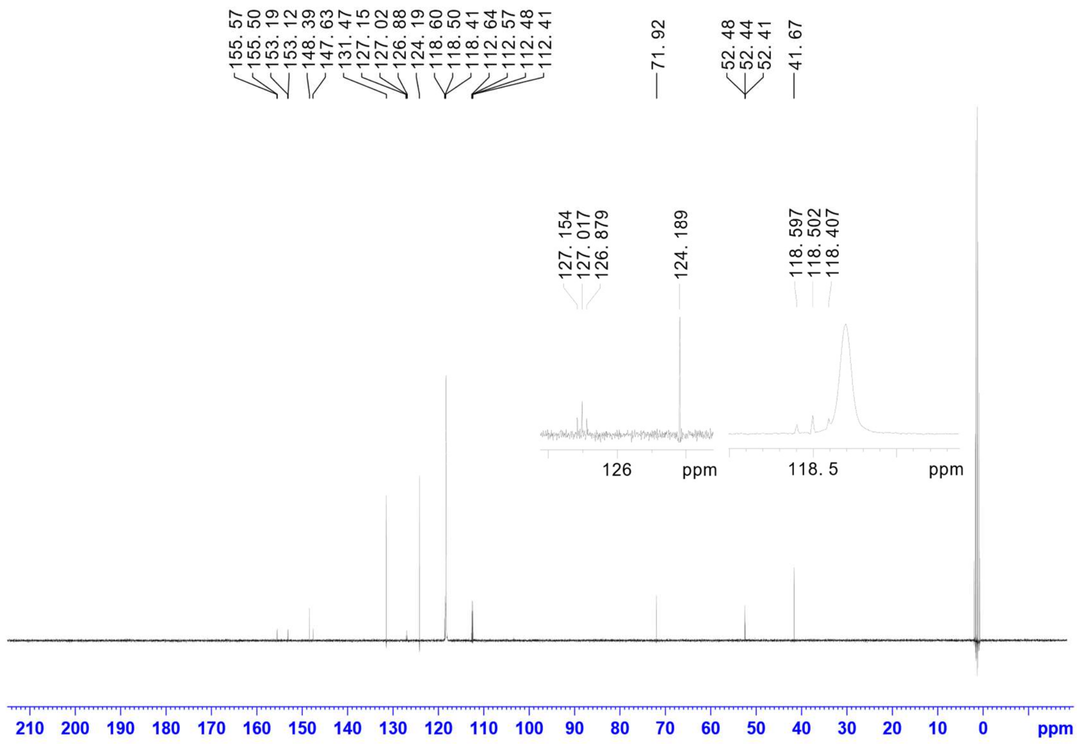

^{19}F NMR spectrum of **14g** (376.46 MHz, CD_3CN)

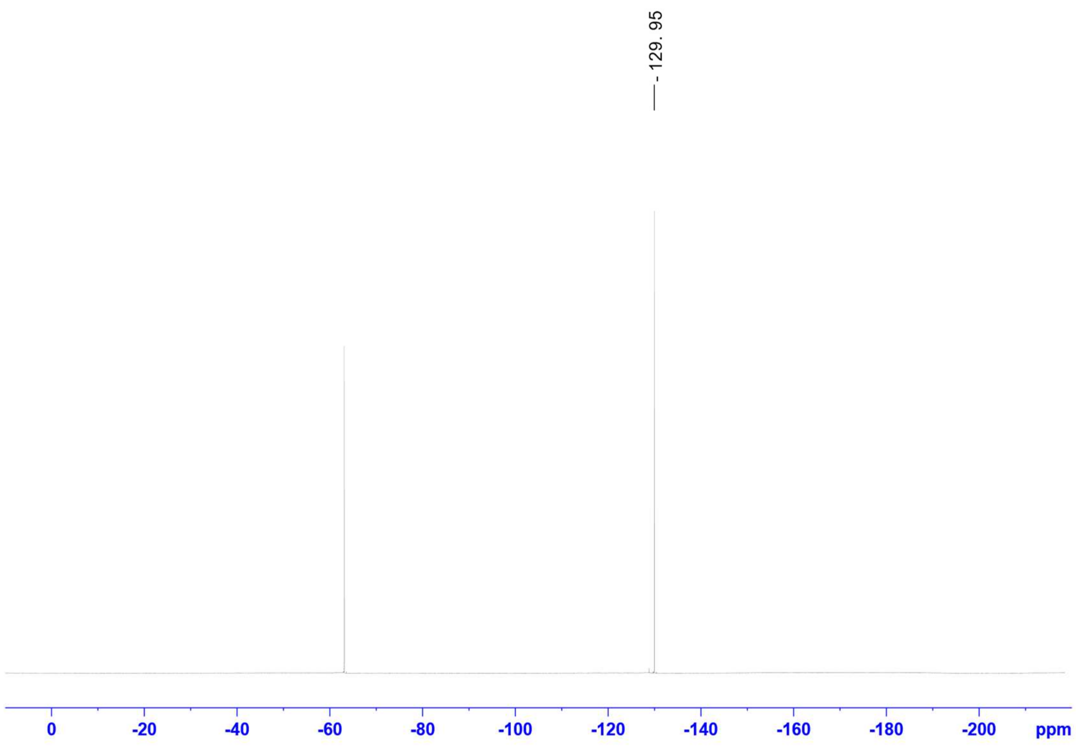

^{1}H NMR spectrum of **14h** (400.13 MHz, CD_3CN)

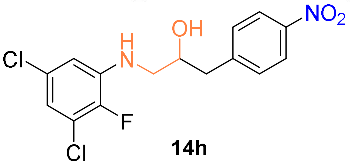

14h

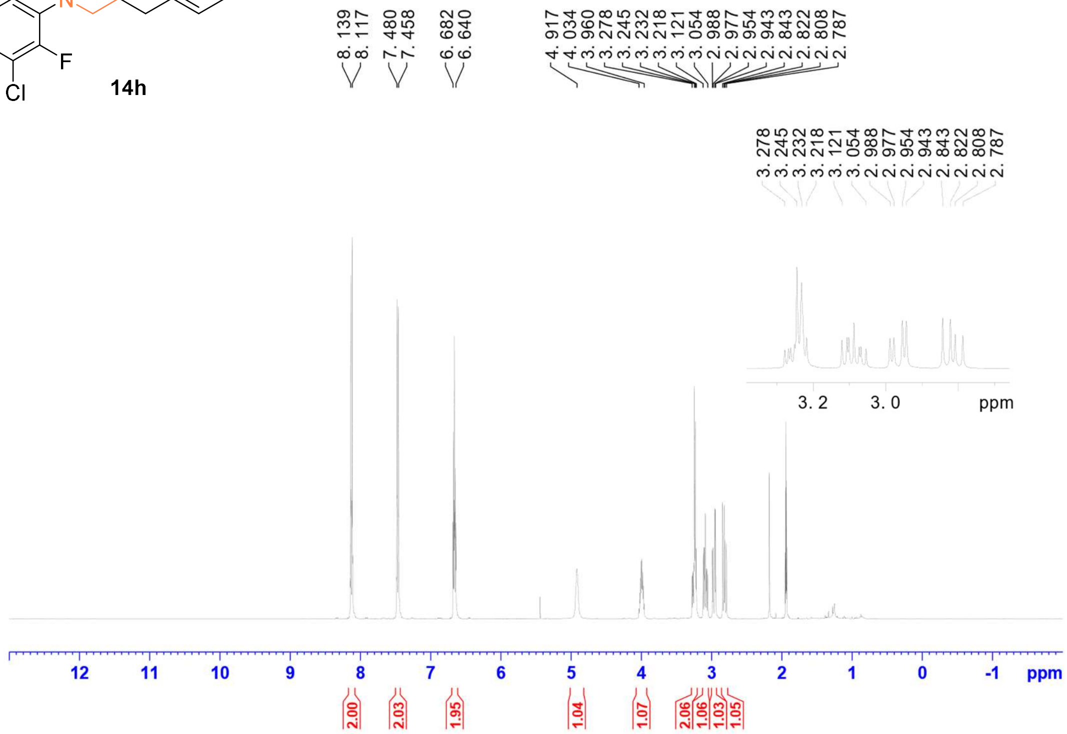

$^{13}C\{^{1}H\}$ NMR spectrum of **14h** (100.61 MHz, CD_3CN)

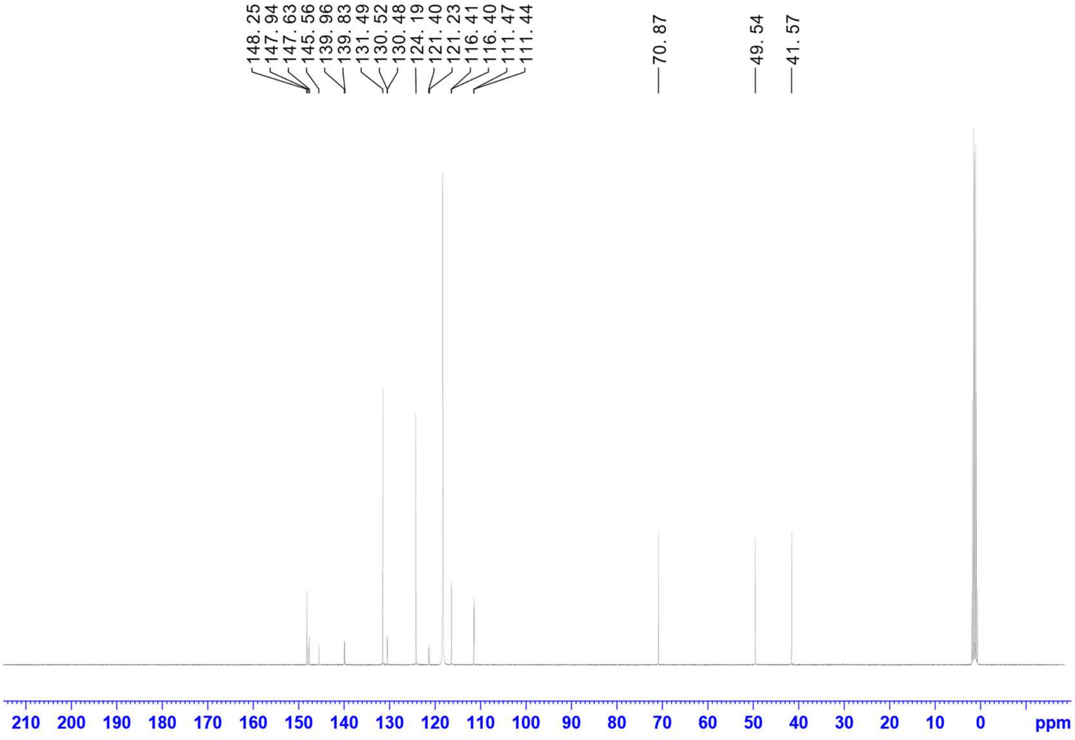

^{19}F NMR spectrum of **14h** (376.46 MHz, CD_3CN)

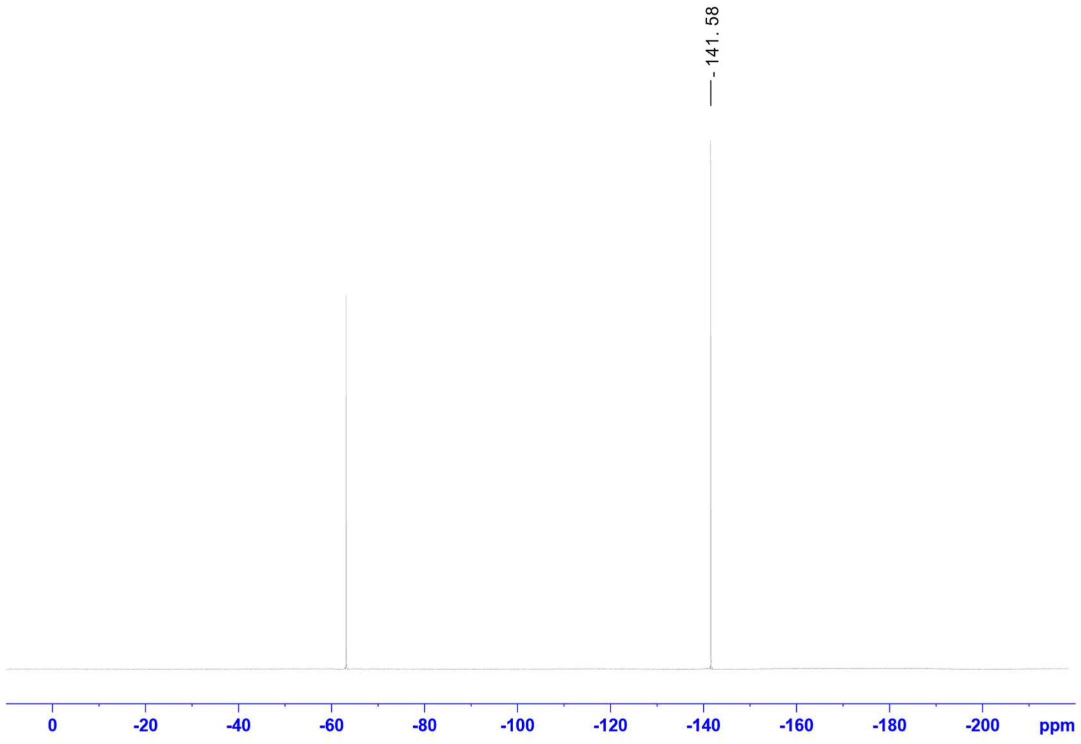

^{1}H NMR spectrum of **14i** (400.13 MHz, CD_3CN)

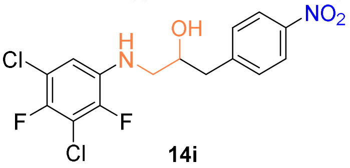

14i

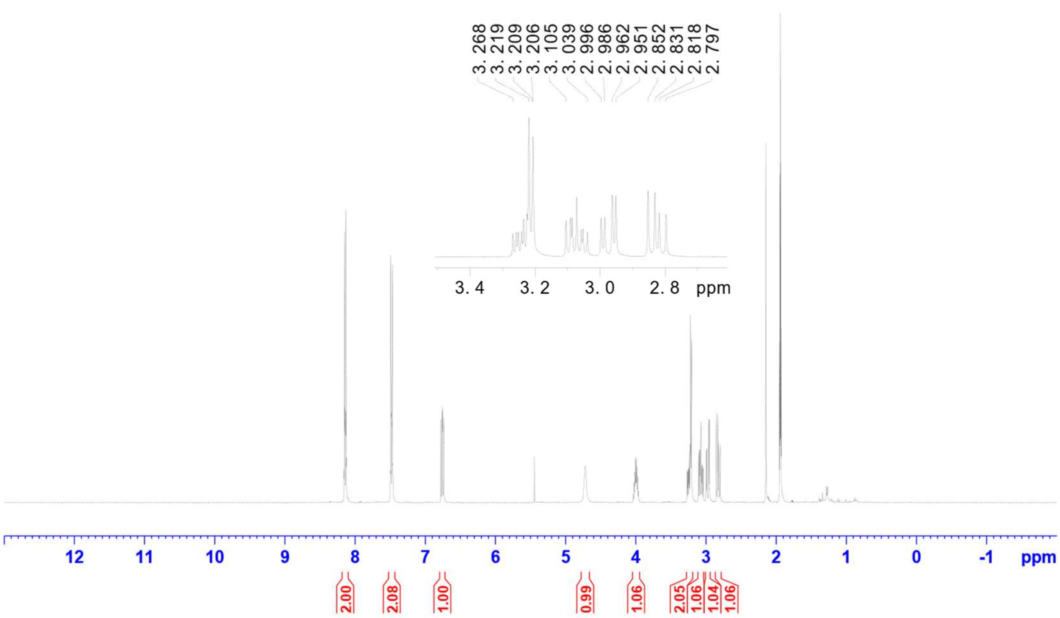

^{13}C{^{1}H} NMR spectrum of **14i** (100.61 MHz, CD_3CN)

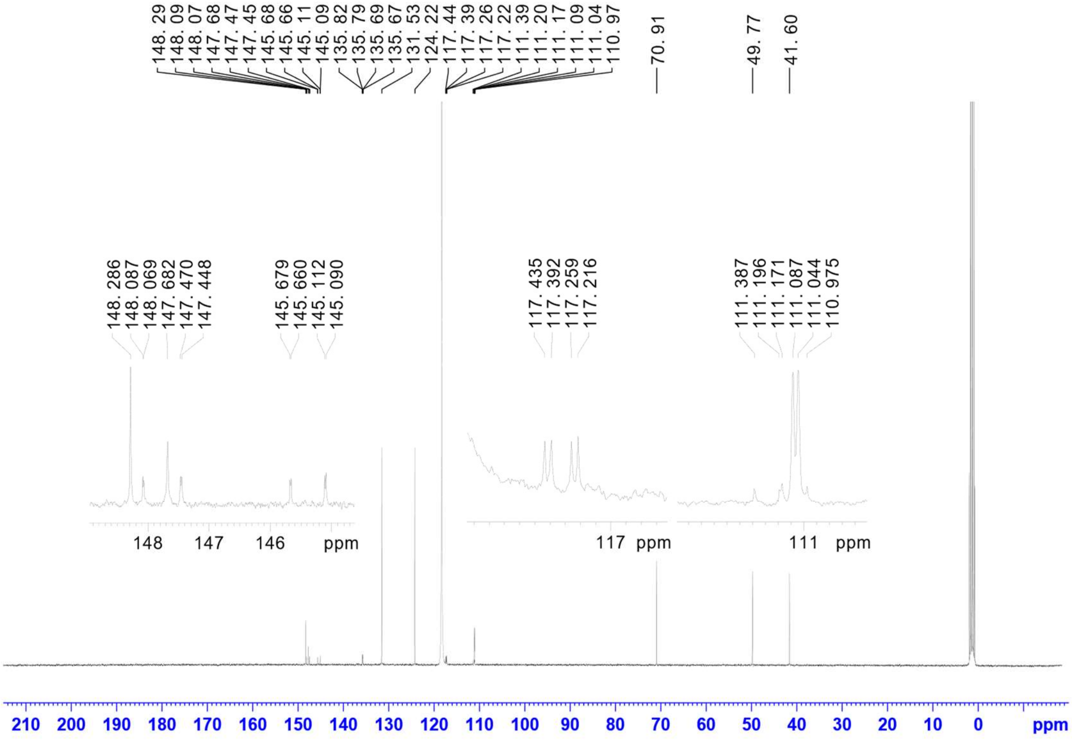

^{19}F NMR spectrum of **14i** (376.46 MHz, CD_3CN)

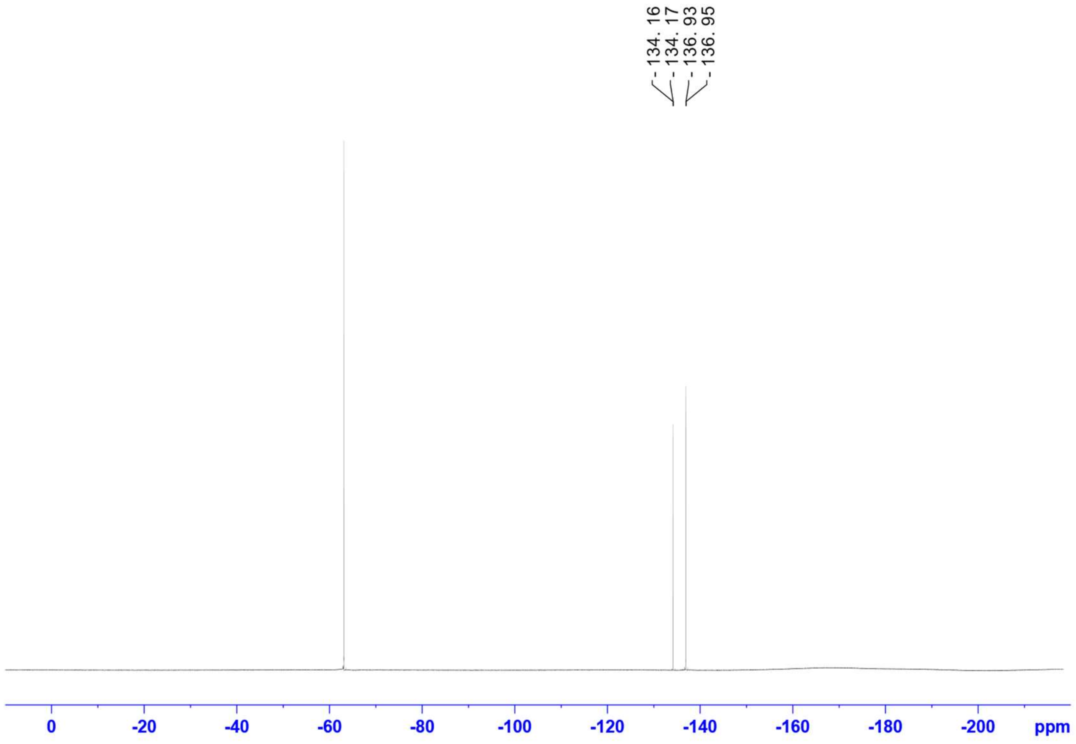

^{1}H NMR spectrum of **14j** (400.13 MHz, CD_3CN)

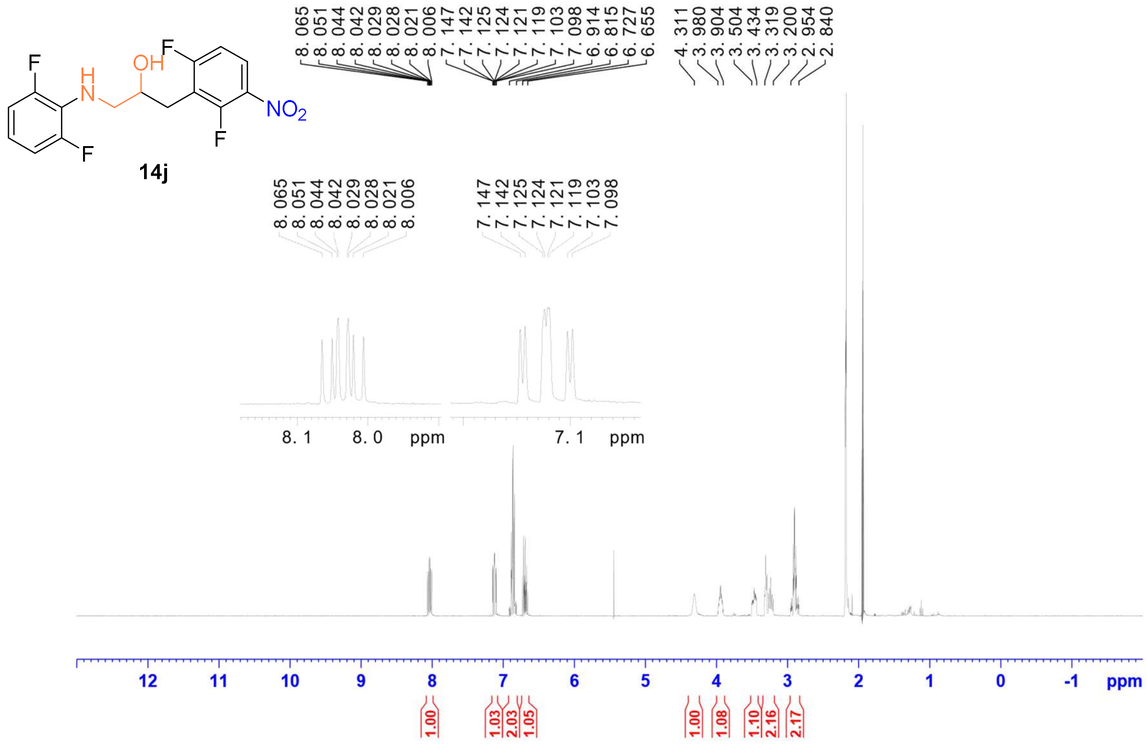

^{13}C{^{1}H} NMR spectrum of **14j** (100.61 MHz, CD_3CN)

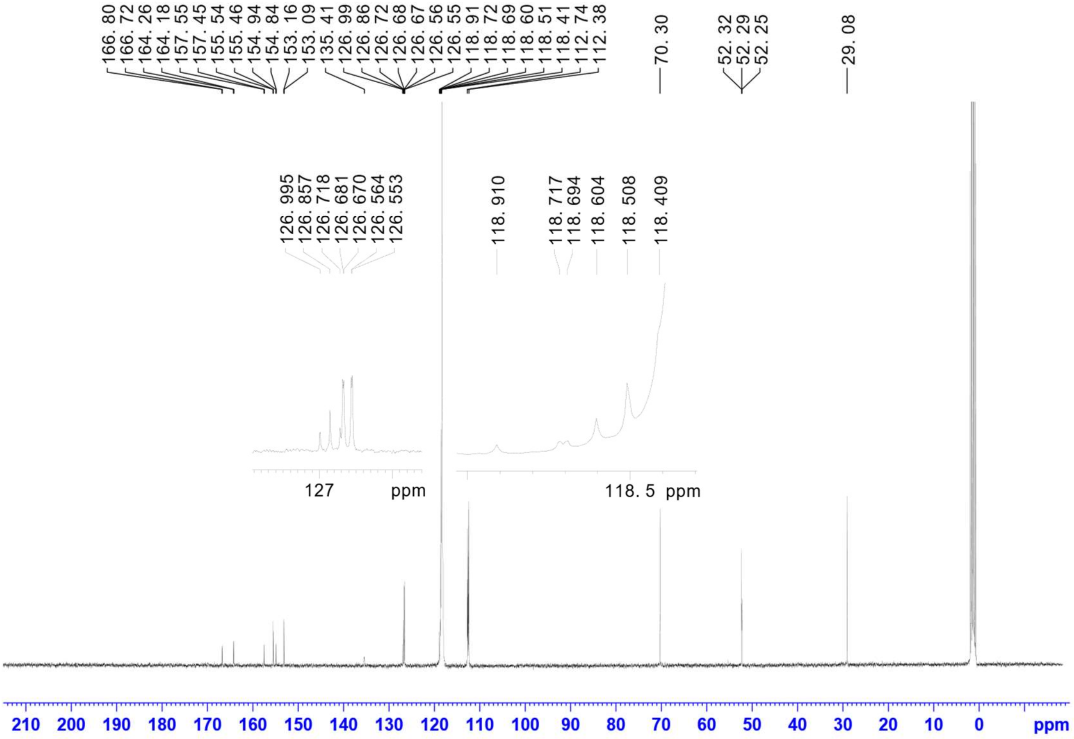

^{19}F NMR spectrum of **14j** (376.46 MHz, CD_3CN)

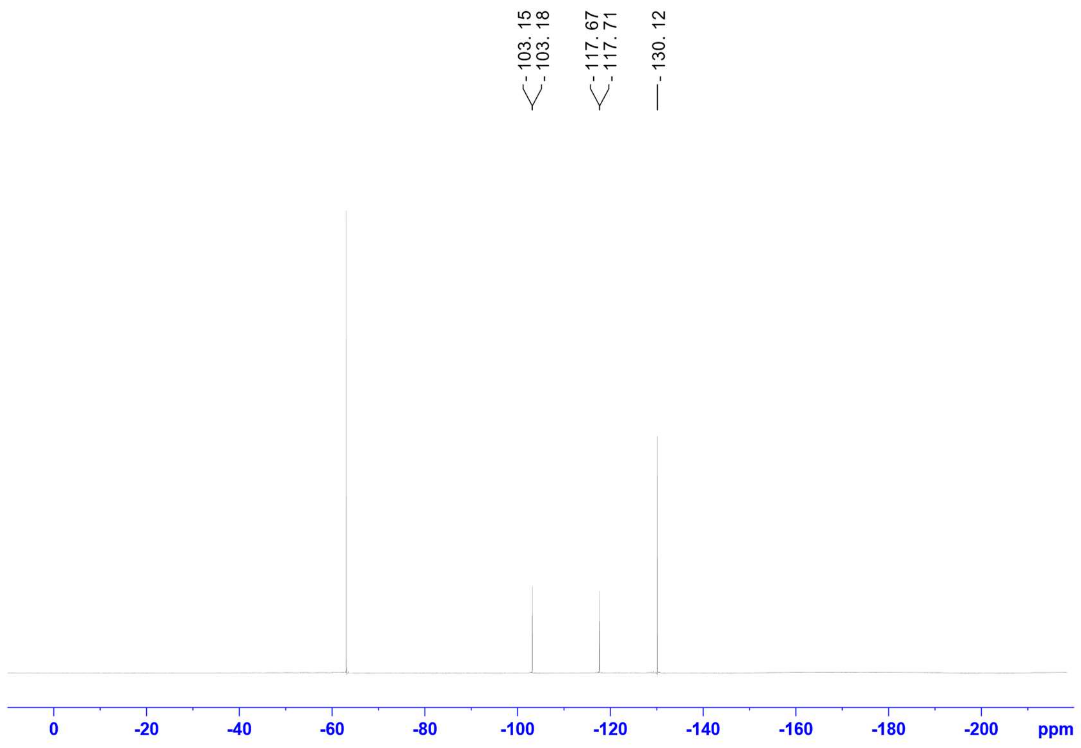

^{1}H NMR spectrum of **14k** (400.13 MHz, $CDCl_3$)

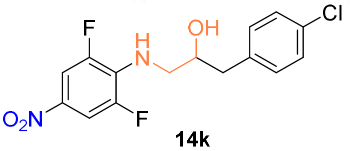

14k

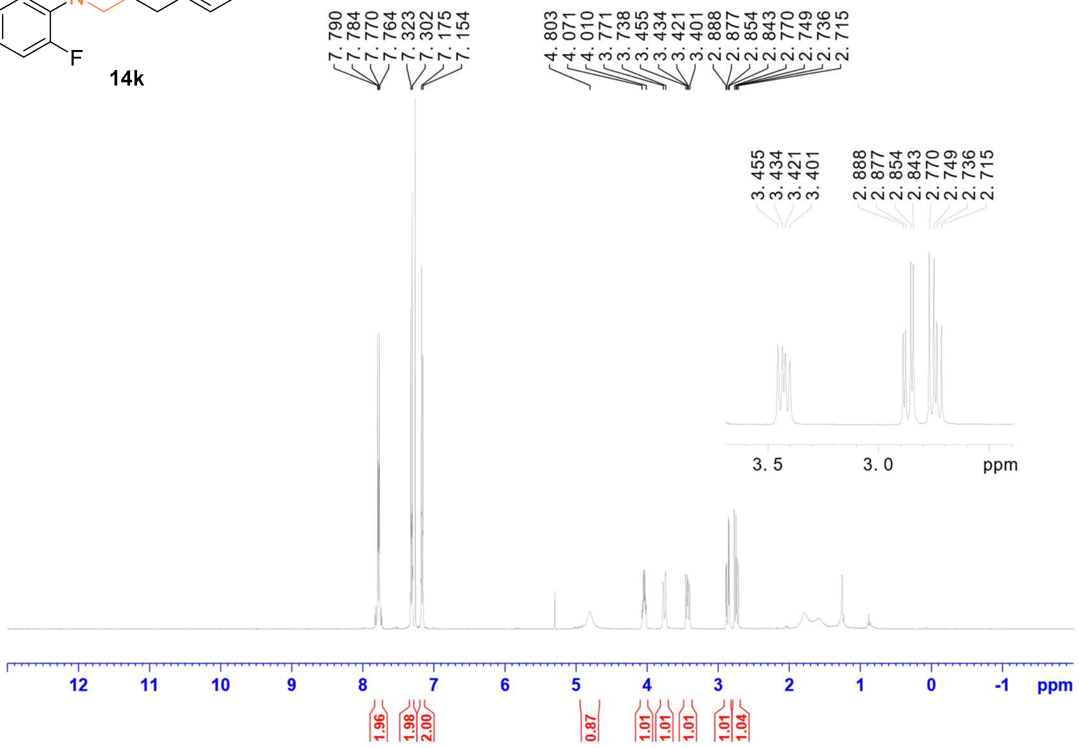

^{13}C{^{1}H} NMR spectrum of **14k** (100.61 MHz, $CDCl_3$)

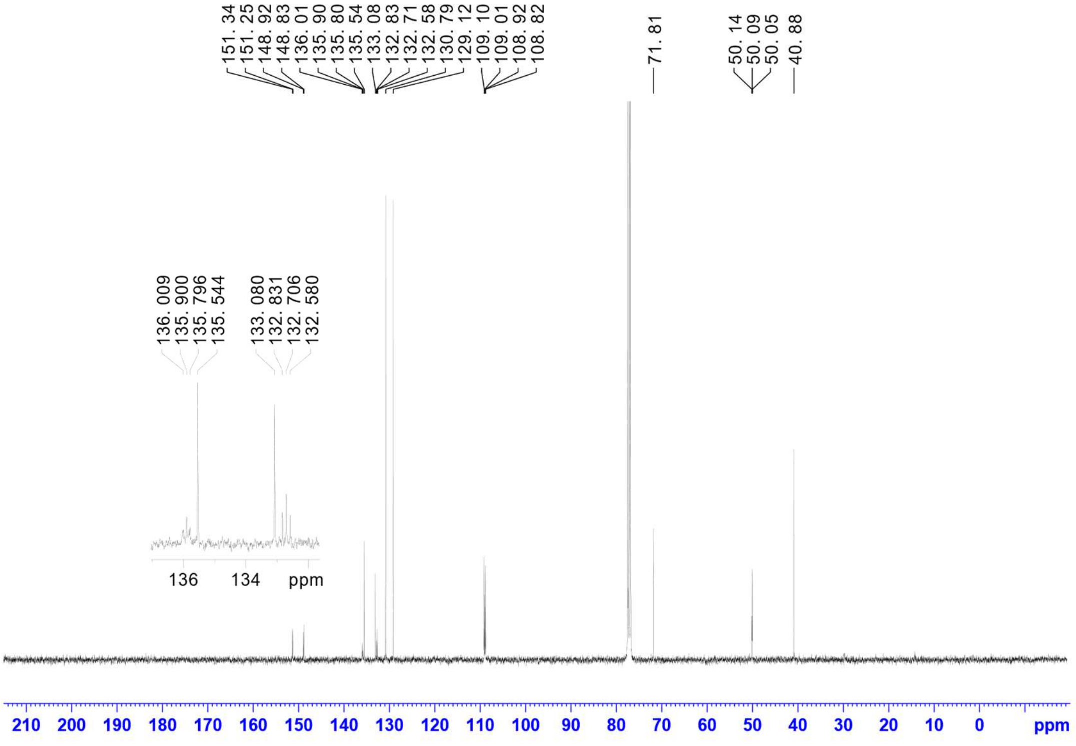

^{19}F NMR spectrum of **14k** (376.46 MHz, $CDCl_3$)

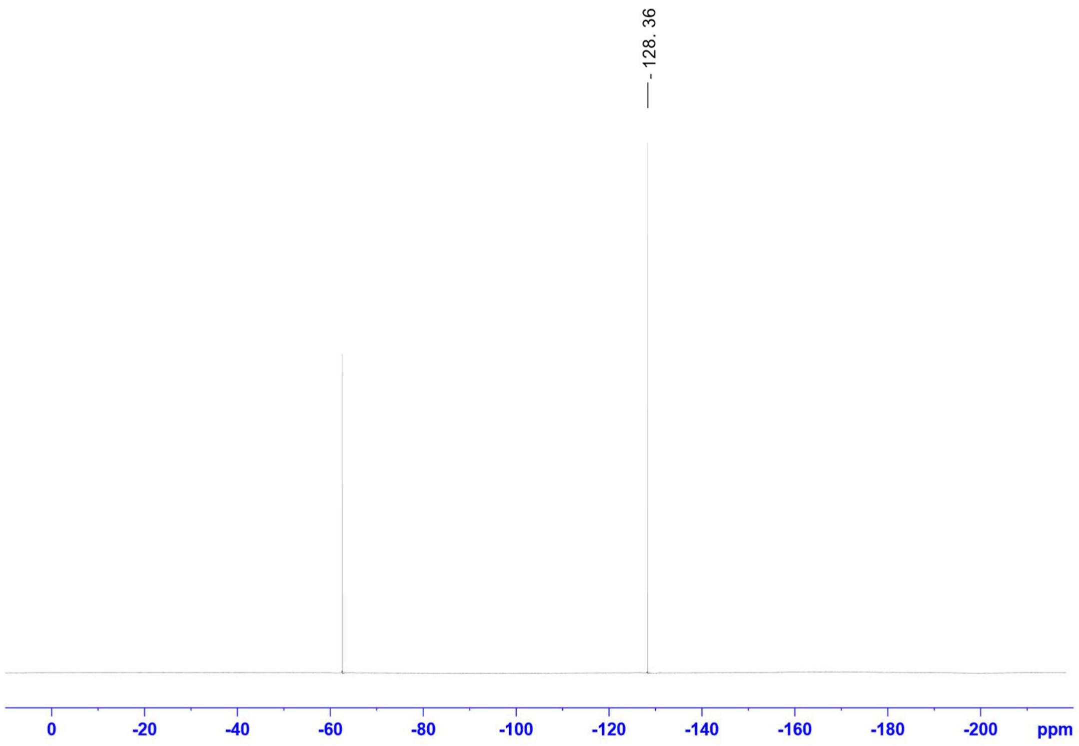

^{1}H NMR spectrum of 1-azido-3-(4-chlorophenyl)propan-2-ol (400.13 MHz, $CDCl_3$)

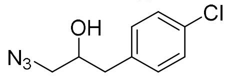

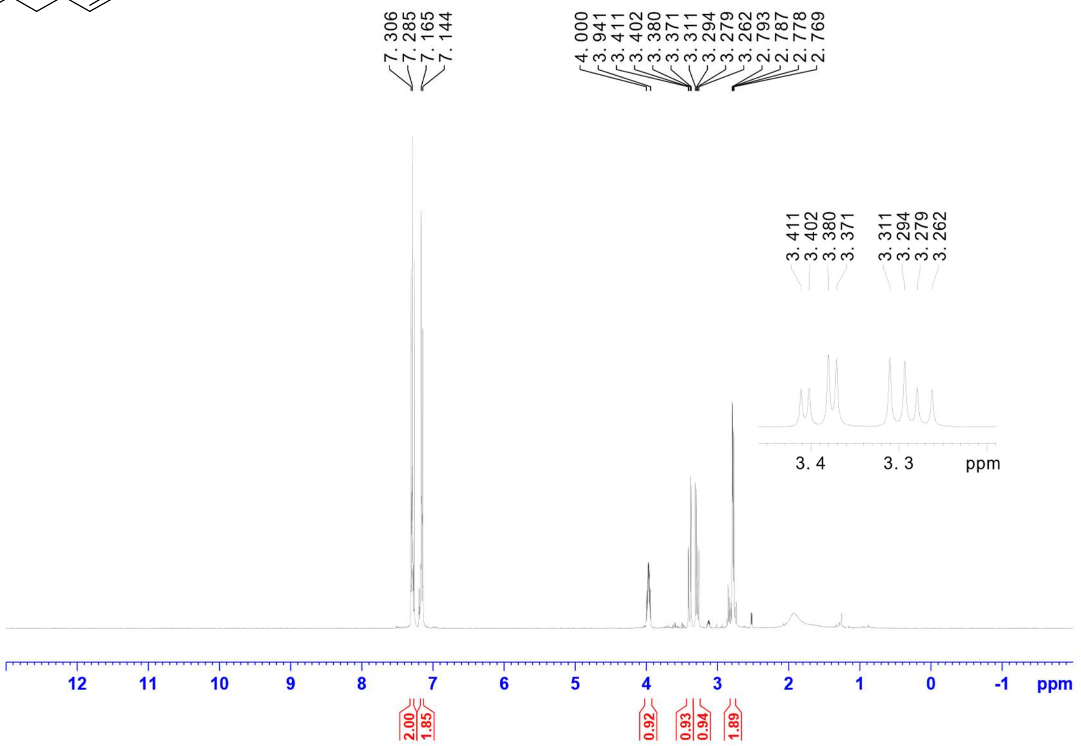

^{13}C{^{1}H} NMR spectrum of 1-azido-3-(4-chlorophenyl)propan-2-ol (100.61 MHz, $CDCl_3$)

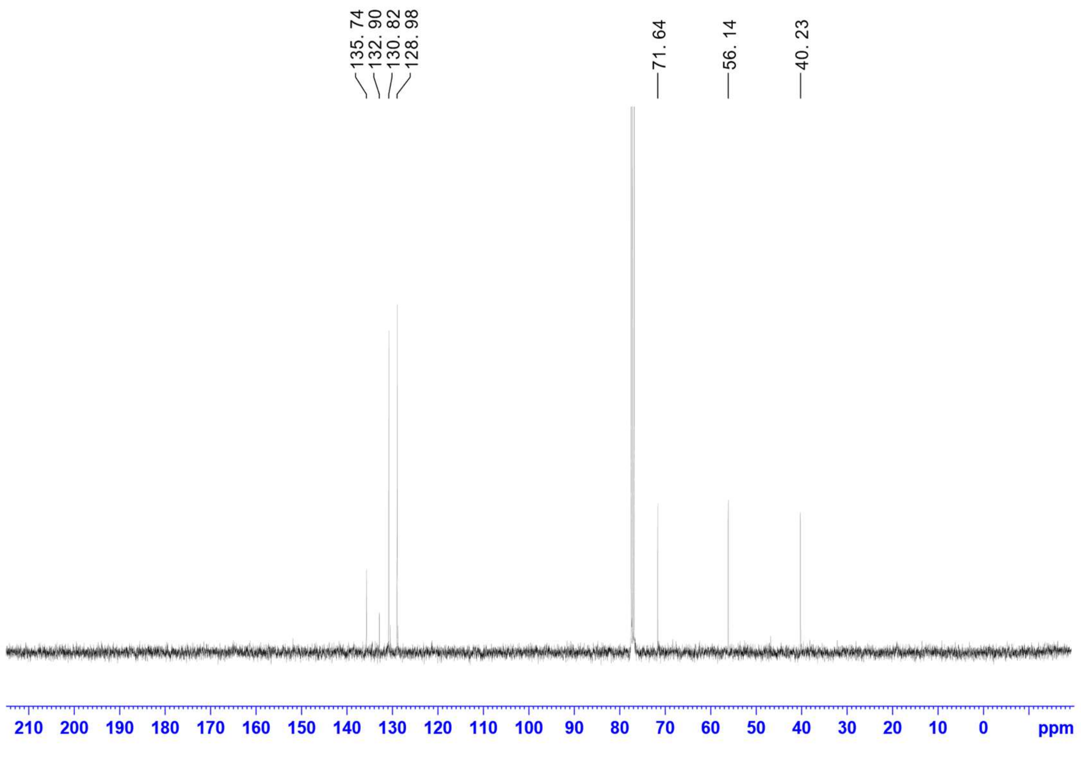

^{1}H NMR spectrum of 1-amino-3-(4-chlorophenyl)propan-2-ol hydrochloride (400.13 MHz, D_2O)

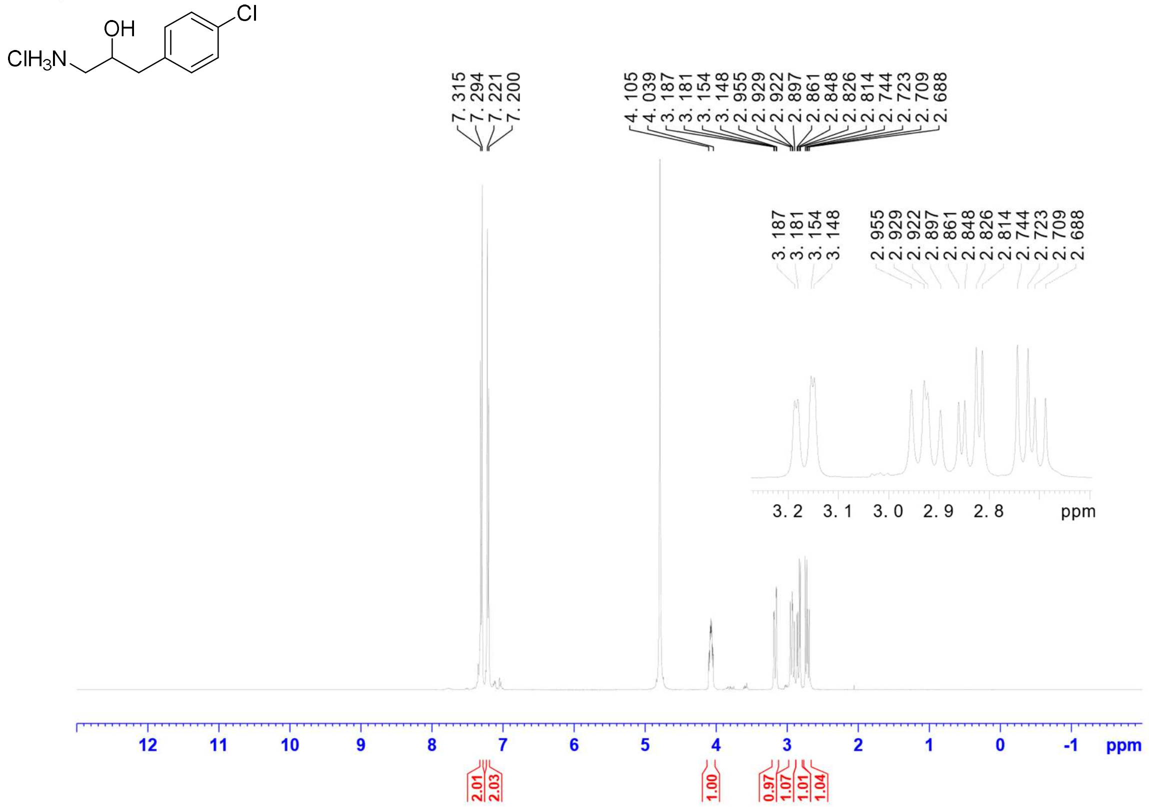

^{13}C{^{1}H} NMR spectrum of 1-amino-3-(4-chlorophenyl)propan-2-ol hydrochloride (100.61 MHz, D_2O)

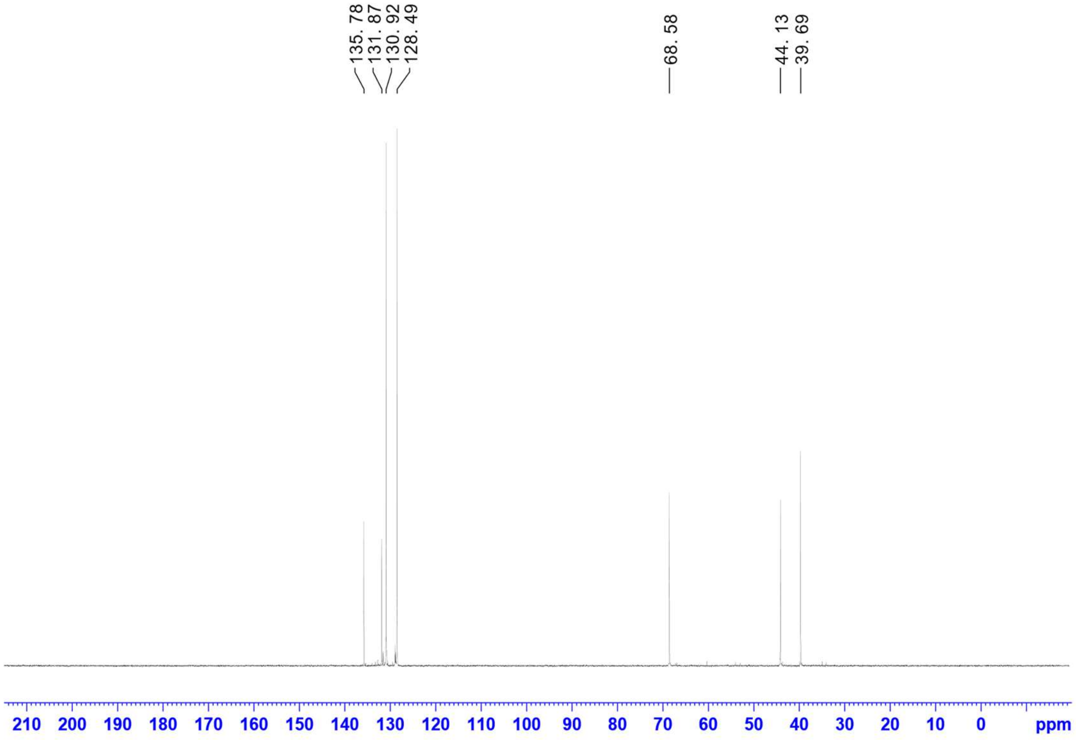

Supplement: Supplementary file 1 — jo2c00681_si_001.pdf [file jo2c00681_si_001.pdf]
